# A tripartite rheostat controls self-regulated host plant resistance to insects

**DOI:** 10.1038/s41586-023-06197-z

**Published:** 2023-06-14

**Authors:** Jianping Guo, Huiying Wang, Wei Guan, Qin Guo, Jing Wang, Jing Yang, Yaxin Peng, Junhan Shan, Mingyang Gao, Shaojie Shi, Xinxin Shangguan, Bingfang Liu, Shengli Jing, Jing Zhang, Chunxue Xu, Jin Huang, Weiwei Rao, Xiaohong Zheng, Di Wu, Cong Zhou, Bo Du, Rongzhi Chen, Lili Zhu, Yuxian Zhu, Linda L. Walling, Qifa Zhang, Guangcun He

**Affiliations:** 1 State Key Laboratory of Hybrid Rice, College of Life Sciences, Wuhan University, Wuhan, China; 2 Hubei Hongshan Laboratory, Wuhan, China; 3 Key Laboratory of Biology and Genetic Improvement of Oil Crops, Oil Crops Research Institute of the Chinese Academy of Agricultural Sciences, Wuhan, China; 4 Institute for Advanced Studies, Wuhan University, Wuhan, China; 5 Department of Botany and Plant Sciences, University of California, Riverside, CA USA; 6 National Key Laboratory of Crop Genetic Improvement, Huazhong Agricultural University, Wuhan, China

**Keywords:** Herbivory, Biotic, Autophagy, Plant signalling

## Abstract

Plants deploy receptor-like kinases and nucleotide-binding leucine-rich repeat receptors to confer host plant resistance (HPR) to herbivores^1^. These gene-for-gene interactions between insects and their hosts have been proposed for more than 50 years^2^. However, the molecular and cellular mechanisms that underlie HPR have been elusive, as the identity and sensing mechanisms of insect avirulence effectors have remained unknown. Here we identify an insect salivary protein perceived by a plant immune receptor. The BPH14-interacting salivary protein (BISP) from the brown planthopper (*Nilaparvata lugens* Stål) is secreted into rice (*Oryza sativa*) during feeding. In susceptible plants, BISP targets *O.* *satvia* RLCK185 (*Os*RLCK185; hereafter *Os* is used to denote *O.* *satvia*-related proteins or genes) to suppress basal defences. In resistant plants, the nucleotide-binding leucine-rich repeat receptor BPH14 directly binds BISP to activate HPR. Constitutive activation of *Bph14*-mediated immunity is detrimental to plant growth and productivity. The fine-tuning of *Bph14*-mediated HPR is achieved through direct binding of BISP and BPH14 to the selective autophagy cargo receptor *Os*NBR1, which delivers BISP to *Os*ATG8 for degradation. Autophagy therefore controls BISP levels. In *Bph14* plants, autophagy restores cellular homeostasis by downregulating HPR when feeding by brown planthoppers ceases. We identify an insect saliva protein sensed by a plant immune receptor and discover a three-way interaction system that offers opportunities for developing high-yield, insect-resistant crops.

## Abstract (web summary)

Insect salivary protein (BISP) targets *Os*RLCK185 to suppress defence in susceptible plants, whereas in resistant plants BISP binds BPH14 to activate host plant resistance. To restore cellular homeostasis, the resistance mechanism is fine-tuned by selective autophagy.

## Main

During the millions of years of plant and insect co-evolution, herbivorous insects have developed diverse feeding strategies to retrieve nutrients from host plants. Such insects can cause up to 18% of annual losses of global crop yield^3^. Because plants are sessile and cannot escape continuous attack by pests, plants have evolved various defence strategies to combat herbivory. Unlike microbial pathogens, insects can move swiftly on or among plants as they feed, making it challenging to understand the mechanisms that underlie their interactions.

Herbivorous insects actively select their feeding sites and secrete saliva during feeding to facilitate nutrient acquisition from host plants^1,4,5^. Some salivary proteins induce host defences^1,5^. In the past decade, plant genes that encode nucleotide-binding leucine-rich repeat (NLR) receptors that confer resistance to piercing and sucking insects have been isolated through map-based cloning^6–12^. NLR receptors function as intracellular immune receptors that directly or indirectly detect cognate effectors^13,14^ and activate effector-triggered immunity (ETI)^6–10^, also known as HPR. The identities of the insect avirulence effectors that trigger ETI have not yet been discovered. Hence, how a host plant recognizes the insect and deploys and modulates resistance remains largely unknown.

The brown planthopper (BPH; *N.* *lugens* Stål) is the most destructive insect pest that threatens rice production globally^15^. Using its stylet mouthparts to probe and penetrate plant cells, it consumes phloem sap and eventually causes plant death and substantial yield losses^16–18^. BPHs migrate long distances, crossing national boundaries in search of better host plants^15–18^, which makes regional control of BPH challenging. For this reason, HPR has been crucial for BPH control. The first BPH-resistance gene isolated in rice, *Bph14*, encodes a coiled-coiled nucleotide-binding leucine-rich repeat (CC-NB-LRR) protein that forms a homomeric complex and interacts with WRKY transcription factors^19^. BPH14 enhances WRKY activity to confer resistance to BPH^19^.

Here we show that the BPH salivary protein BISP is directly recognized by BPH14. Notably, the perception of BISP not only triggers *Bph14*-mediated resistance but also activates NBR1-mediated selective autophagy. This results in the degradation of BISP to restrict hyperactivation of *Bph14*-regulated defences. We elucidate the molecular and cellular interactions that occur after BPH14 binds to the BISP effector to activate and modulate *Bph14*-mediated HPR.

## BPH BISP interacts with rice BPH14

**Fig. 1:**
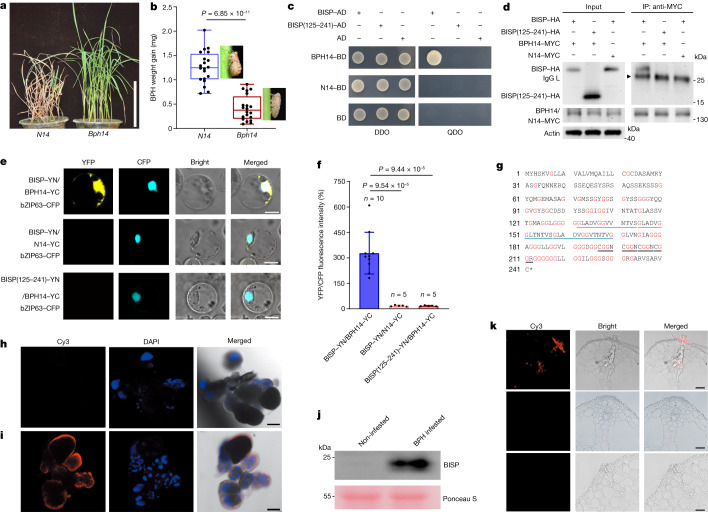
The BPH salivary protein BISP interacts with BPH14 and is delivered into rice. **a**, Phenotypes of rice plants carrying *Bph14* and *N14* after 7 days of BPH infestation. **b**, Weight gain and representative images of BPH insects feeding on *Bph14* and *N14* plants for 2 days (*n* = 20, biologically independent samples). The box limits indicate the 25th and 75th percentiles, the whiskers indicate the full range of the data and the centre line indicates the median. Individual data points are plotted. **c**–**e**, BISP interacted with BPH14 in Y2H (**c**), co-IP (**d**) and BiFC (**e**) assays. N14 and BISP(125–241) served as negative controls. DDO, SD/-Leu-Trp; QDO, SD/-Leu-Trp-His-Ade. bZIP63–CFP, nuclear marker. **f**, Quantification of relative YFP intensities in BiFC assays. Data are the mean ± s.d. (*n* = numbers of biologically independent cells). **g**, Amino acid sequence of BISP. The asterisk indicates the stop codon. Glycine residues are marked in red. The 13-amino acid and 4-amino acid tandem repeats are underlined in blue and black, respectively. **h**,**i**, Immunohistochemical localization of BISP in female BPH salivary glands using pre-immune rabbit serum (**h**) or anti-BISP antibodies (**i**). Red fluorescence (Cy3) and blue fluorescence show the localization of BISP and DAPI-stained nuclei, respectively. **j**, BISP was delivered into rice leaf sheaths during BPH infestation. Leaf sheath proteins were analysed using anti-BISP antibodies. Ponceau S staining served as the loading control. **k**, Immunohistochemical staining showing BISP in BPH-infested rice leaf sheaths. The non-infected (middle) and BPH-infested (bottom) sheaths were detected by anti-BISP antibodies and pre-immune rabbit serum, respectively, served as negative controls. In **b** and **f**, *P* values were derived by one-way analysis of variance (ANOVA). Experiments (**a**–**e**,**h**–**k**) were repeated at least three times, each giving similar results. The results of the other two repeats are presented in Supplementary Fig. 2. Scale bars, 5 μm (**e**), 25 μm (**k**), 100 μm (**h**,**i**) or 10 cm (**a**). Source data

**Extended Data Fig. 1:**
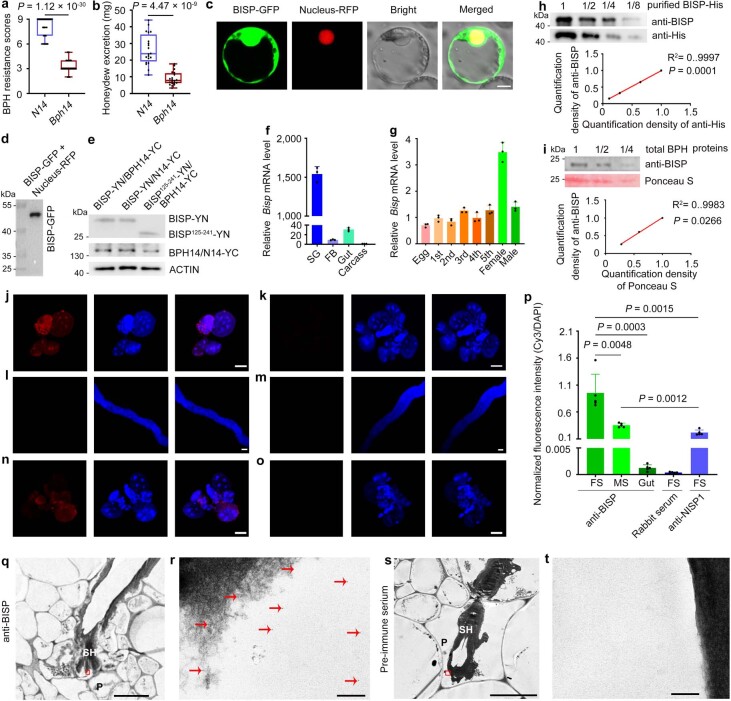
Host-plant resistance evaluation, BISP subcellular localization in rice protoplasts, *Bisp* gene and BISP protein expression profile in BPH, and BISP delivery into rice leaf sheath. **a**, BPH-resistance scores of *Bph14* and *N14* plants after BPH infestation. *Bph14* and *N14* plants express the resistant NLR (BPH14) and the susceptible allele variant (N14), respectively. Lower scores indicate higher resistance to the insects. Data were collected 7 days after BPH infestation (*n* = 36 plants examined over 3 independent experiments). *P* values were derived by one-way ANOVA. **b**, Honeydew excretion of BPHs fed on *Bph14* and *N14* plants for 2 days. *P* values were derived by one-way ANOVA (*n* = 20, biologically independent plants). **c**, BISP-GFP had a nucleocytoplasmic localization in rice protoplasts. bZIP63-RFP was used as a nuclear marker. The confocal images were taken 14–20 h after transformation. Scale bar, 5 μm. **d**, Immunoblot detection of full-length BISP-GFP expression in rice protoplasts. BISP-GFP was detected by anti-GFP antibody. **e**, Immunoblot detection of the expressed proteins in BiFC assays. BISP-YN, BISP^125–241^-YN(BISP(125–241)-YN), and BPH14-YC or N14-YC were co-expressed in rice protoplasts and the total proteins were detected using anti-Myc, anti-HA or anti-ACTIN antibody. **f**, Relative levels of *Bisp* RNA in different BPH tissues. SG, salivary glands; FB, fat body. Data are mean ± s.d. (*n* = 3, biologically independent experiments). **g**, Relative level of *Bisp* RNAs during different developmental stages. 1st to 5th, are first to fifth instars; Female and Male are adults. Data are mean ± s.d. (*n* = 3, biologically independent experiments). **h**, (*Upper*) Determination of the anti-BISP specificity using purified BISP-GST-His. The purified BISP-GST-His protein was serially diluted and detected by anti-BISP or anti-His antisera. (*Bottom*) Correlation between the anti-BISP labeled signal and anti-His labeled signals. The densities of anti-BISP and anti-His labeled signals were quantified using ImageJ with the undiluted sample set as 1. **i**, (*Upper*) Determination of the anti-BISP specificity using a dilution series of total mixed adults BPH proteins. BISP was detected using the anti-BISP antiserum. Ponceau staining (Ponceau S) is a loading control. (*Bottom*) Correlation between the anti-BISP labeled and Ponceau S signals. The density of anti-BISP labeled and Ponceau S signals were quantified using ImageJ with the undiluted sample set as 1. **j**,**k**, Immunohistochemical localization of BISP in male BPH salivary glands. Anti-BISP (**j**) or pre-immune rabbit serum (**k**) was used as the primary antiserum. Red fluorescence (Cy3) shows the localization of BISP and blue fluorescence shows the location of DAPI-stained nuclei. Scale bars, 100 μm. **l**,**m**, Immunohistochemical localization of BISP in mixed adults BPH guts. Anti-BISP (**l**) or pre-immune rabbit serum (**m**) was used as the primary antiserum. Red fluorescence (Cy3) shows the localization of BISP and blue fluorescence shows the location of DAPI-stained nuclei. Scale bars, 100 μm. **n**,**o**, Immunohistochemical localization of *Nl*SP1 in female BPH salivary glands. Either anti-*Nl*SP1 (**n**) or pre-immune rabbit serum (**o**) was used as the primary antiserum. Red fluorescence (Cy3) shows the localization of NlSP1 and blue fluorescence shows the location of DAPI-stained nuclei. Scale bars, 100 μm. **p**, Quantification of Cy3 intensities. The Cy3 fluorescence in samples using anti-BISP, anti-*Nl*SP1 and pre-immune serum was normalized relative to DAPI fluorescence (blue) intensity. *P* values were derived by one-way ANOVA. Data are mean ± s.d. (*n* = 5, biologically independent experiments). **q**-**t**, Immunogold electron microscopy analysis of the distribution of BISP in the rice leaf sheaths during BPH feeding. **q**,**s**, TEM image of the BPH salivary sheaths in the rice phloem tissue. Either anti-BISP (**q**) or pre-immune rabbit serum (**s**) was used as the primary antiserum. Scale bars, 20 μm. **r**,**t**, TEM image of the boxed area in (**q**) and (**s**). Red arrows indicate the anti-BISP-labeled immunogold particles (dark spots). Scale bars, 200 nm. SH, salivary sheaths; P, phloem cells. In box plots in **a**,**b**, the box limits indicate the 25th and 75th percentiles, the whiskers indicate the full range of the data, and the center line indicates the median. Individual data points are plotted. Experiments were independently repeated two (**q**–**t**) or three (**b**–**e**, **h**–**o**) times, each giving similar results. Source data

*Bph14* has been widely used in rice breeding programmes, and many *Bph14*-resistant varieties have been released for rice production (Supplementary Table 1). Rice plants carrying *Bph14* show little damage, whereas susceptible *N14* plants die 7 days after BPH infestation (Fig. 1a and Extended Data Fig. 1a). Insect feeding and growth were inhibited on *Bph14* plants, which resulted in lower weight gain and honeydew excretion than insects feeding on *N14* plants expressing the susceptible BPH14 protein form N14 (Fig. 1b and Extended Data Fig. 1b). N14 shares 83% sequence identity with BPH14 (ref. ^8^). As *Bph14* encodes a typical NLR receptor^8,19^, a BPH effector protein may be recognized by BPH14 to activate *Bph14*-mediated resistance.

Planthoppers secrete salivary proteins into rice plants that may induce or suppress host defence responses during feeding^1,4,5,16^. Yeast two-hybrid (Y2H) screens identified BPH-secreted proteins (effectors) that interacted with BPH14. Of the 12 genes identified, one encoded the *Bisp* transcript that was abundant in the transcriptome of the BPH salivary gland (Supplementary Table 2). BISP interacted with BPH14, but not N14, based on repeated Y2H and co-immunoprecipitation (co-IP) experiments (Fig. 1c,d). Expression of BISP tagged with green fluorescent protein (BISP–GFP) in rice protoplasts showed that it localized to the nucleocytoplasm (Extended Data Fig. 1c,d), which is consistent with the subcellular localization of BPH14 (ref. ^19^). In addition, bimolecular fluorescence complementation (BiFC) confirmed the interaction between BISP and BPH14 and their nucleocytoplasmic co-localization in rice protoplasts (Fig. 1e,f and Extended Data Fig. 1e).

*Bisp* (LOC111051577) resides on chromosome 12 of BPH and encodes a 241-amino acid protein with an amino-terminal signal peptide (amino acids 1–25) and no transmembrane domain (Fig. 1g), which suggests that BISP is a secretory protein. Rich in glycine residues (32%), BISP has three 13-amino acid (GLADVGGLTNTVS) and 4-amino acid (CGGN/R) tandem repeats in its carboxy-terminal region (Fig. 1g). Quantitative PCR with reverse transcription (RT–qPCR) confirmed that *Bisp* is highly expressed in salivary glands and in whole female adults (Extended Data Fig. 1f,g). Immunohistochemical analysis using BISP antiserum revealed that BISP accumulated to higher levels in female salivary glands than in male salivary glands and guts (Fig. 1h,i and Extended Data Fig. 1h–m,p), a result that was consistent with *Bisp* RNA levels. Moreover, BISP signals were higher than the salivary protein NlSP1 (Extended Data Fig. 1n–p).

BPHs were allowed to feed on rice plants to determine whether BISP is secreted into rice leaf sheaths. Immunoblots detected BISP in leaf sheath protein extracts from BPH-infested but not non-infested plants (Fig. 1j). Immunolocalization assays verified the delivery of BISP into rice leaves. BISP was detected along the penetration path of stylets within the leaf sheaths (Fig. 1k). Immunogold electron microscopy further confirmed that BISP was secreted into rice tissues during BPH feeding (Extended Data Fig. 1q–t).

## BISP suppresses plant defence

**Extended Data Fig. 2:**
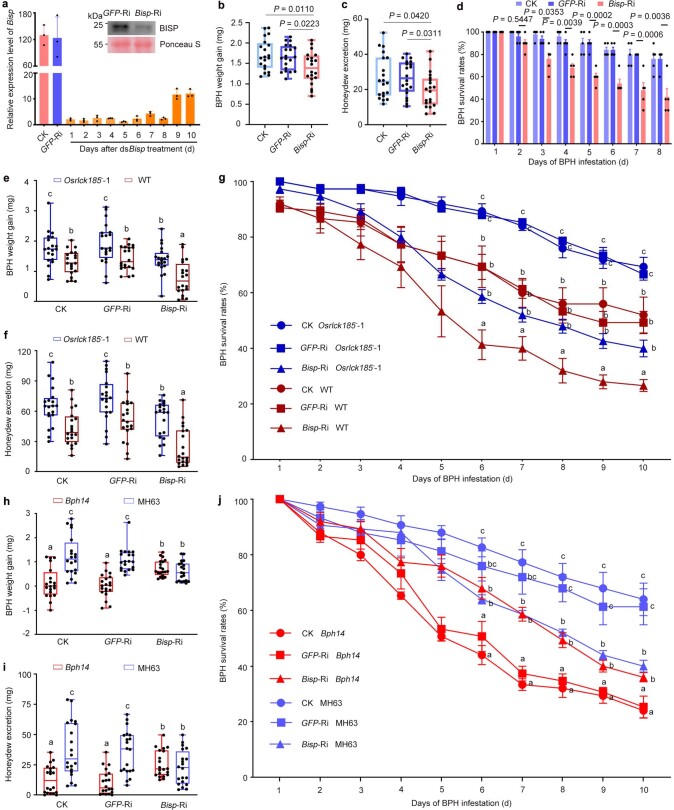
BISP played crucial roles in the feeding and performance of BPH on rice plants. **a**, Effects of dsRNA injection on *Bisp* transcript levels in BPHs. *Bisp*-Ri (*Bisp*-RNAi) insects were assayed daily for 10 days. *GFP-*Ri (*GFP*-RNAi) and CK insects were assayed on day 3. Data are mean ± s.d. (*n* = 3, biologically independent experiments). The inset shows a representative immunoblot demonstrating the relative abundance of BISP in *GFP*-Ri BPHs and *Bisp*-Ri BPHs at 3 days after injection. Total BPH proteins were extracted from injected or control insects and immunoblotted. BISP was detected with anti-BISP antibodies and Ponceau S staining was used as the loading control. **b**,**c**,**d**, Weight gain (**b**), honeydew excretion (**c**) and survival rates (**d**) of dsRNA-injected and non-injected BPHs fed on *N14* plants. **e**,**f**,**g**, Weight gain (**e**), honeydew excretion (**f**) and survival rates (**g**) of dsRNA-injected and non-injected BPHs fed on the *Osrlck185*^*−*^-1 (Os*rlck185*-1) and WT plants. **h**,**i**,**j**, Weight gain (**h**), honeydew excretion (**i**) and survival rates (**j**) of dsRNA-injected BPHs fed on the *Bph14* and MH63 plants. In **a**–**j**, BPHs were microinjected with *Bisp* dsRNA (*Bisp*-Ri) or *GFP* dsRNA (*GFP*-Ri) and non-injected BPHs (CK) served as a control. For **b**–**j**, *P* values were derived by one-way ANOVA. In **b**,**c**,**e**,**f**,**h**,**i**, data are mean ± s.d. (*n* = 20, biologically independent plants); in **d**,**g**,**j**, data are mean ± s.e.m. (*n* = 5, biologically independent experiments). In box plots in **b**,**c**,**e**,**f**,**h**,**i**, the box limits indicate the 25th and 75th percentiles, the whiskers indicate the full range of the data, and the center line indicates the median. Individual data points are plotted. In **e**–**j**, different letters indicate significant differences (*P* < 0.05, one-way ANOVA); exact P values are provided in Supplementary Table 4. All the experiments were repeated three times, each giving similar results. Source data

To determine whether BISP influenced the success of BPH on rice, we disrupted *Bisp* expression by microinjecting *Bisp* double-stranded RNAs (dsRNAs) into individual BPHs to induce RNA-mediated interference (RNAi). *Bisp* transcripts and proteins were significantly reduced in BPHs that received *Bisp*-RNAi compared with those that received *GFP*-RNAi (Extended Data Fig. 2a). *Bisp*-RNAi insects exhibited lower weight gain and honeydew excretion (Extended Data Fig. 2b,c). These insects also experienced significantly higher mortality when fed on the susceptible *N14* rice plants than BPHs that received no injection (controls) or *GFP*-RNAi (Extended Data Fig. 2d). Therefore, *Bisp* plays a crucial role in the feeding and performance of BPHs on susceptible rice plants.

**Fig. 2:**
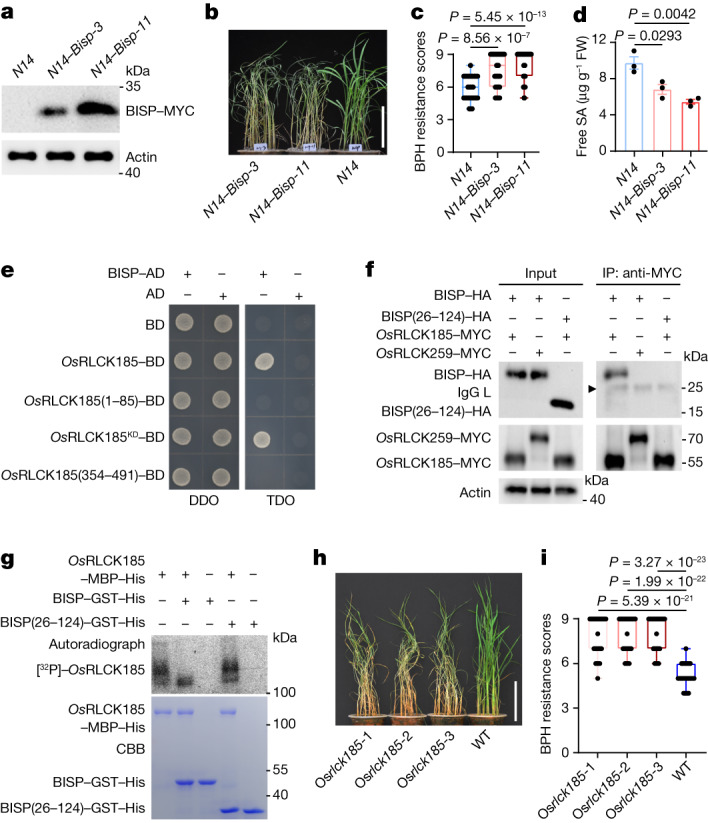
BISP interacts with *Os*RLCK185 and suppresses rice defence responses. **a**, Immunoblot detection of BISP in *N14* and *N14–Bisp* transgenic rice lines. **b**,**c**, Phenotypes (**b**) and BPH resistance scores (**c**) of *N14* and *N14–Bisp* plants after 4 days of BPH infestation. *n* = 36 plants examined over 3 independent experiments. **d**, Endogenous free SA levels in *N14–Bisp* and *N14* plants. FW, fresh weight. Data are the mean ± s.d. (*n* = 3, biologically independent experiments). **e**, BISP interacted with *Os*RLCK185 and its kinase domain in a Y2H assay. *Os*RLCK185(1–85), *Os*RLCK185^KD^, *Os*RLCK185(354–491), *Os*RLCK185 deletion mutants; KD, kinase domain (amino acids 86–353); TDO, SD/-Leu-Trp-His with 1.5 mM 3-AT (3-amino-1,2,4-triazole). **f**, Co-IP showing the interaction between BISP and *Os*RLCK185. BISP(26–124) and *Os*RLCK159 served as negative controls. **g**, *Os*RLCK185 autophosphorylation activity was reduced by BISP. BISP(26–124)–GST–His proteins served as the negative control. Kinase activity was detected by autoradiography and input proteins shown after Coomassie Brilliant Blue staining (CBB). **h**,**i**, Phenotypes (**h**) and BPH-resistance scores (**i**) of Os*rlck185* and WT plants after 4 days of BPH infestation. *n* = 45 plants examined over 3 independent experiments. In box plots in **c** and **i**, the box limits indicate the 25th and 75th percentiles, the whiskers indicate the full range of the data and the centre line indicates the median. Individual data points are plotted. In **c**,**d** and **i**, *P* values were derived by one-way ANOVA. The experiments (**a**,**b**,**d**–**h**) were repeated at least three times, each giving similar results. Scale bars, 10 cm (**b**,**h**). Source data

**Extended Data Fig. 3:**
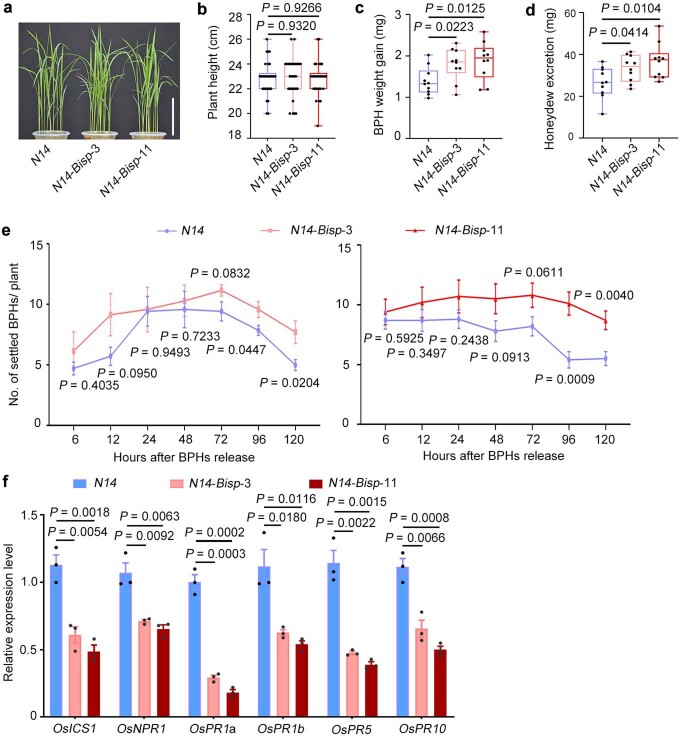
Effects of *Bisp* on BPH resistance in *N14*-*Bisp* transgenic lines. **a**, Photographs of the independent *N14*-*Bisp* homozygous T_2_ transgenic lines (*N14*-*Bisp*-3 and *N14*-*Bisp*-11) and *N14* plants at the 3-leaf stage without BPH feeding. Scale bar, 10 cm. **b**, Plant height of *N14*-*Bisp* lines and *N14* at the 3-leaf stage. *P* values were derived by one-way ANOVA (*n* = 30, biologically independent samples). **c**, **d**, Weight gain (**c**) and honeydew excretion (**d**) of BPHs reared on the *N14*-*Bisp* transgenic and *N14* plants for 2 d. *P* values were derived by one-way ANOVA (*n* = 10, biologically independent samples). **e**, Results of the two-host choice assay examining the settling of BPHs on the *N14*-*Bisp* transgenic and *N14* plants. *P* values were derived by one-way ANOVA. Data are means ± s.d. (for *N14-3* and *N14-Bisp*-11, *n* = 7 and 10, respectively, biologically independent experiments). **f**, Transcript levels analysis of SA biosynthesis- and signaling-related genes *OsICS1* and *OsNPR1* and defense-related genes *OsPR1a*, *OsPR1b*, *OsPR5* and *OsPR10* in two *N14*-*Bisp* transgenic lines and *N14* plants. *P* values were derived by one-way ANOVA. Data are mean ± s.d. (*n* = 3, biologically independent experiments). In box plots in **b**, **c**, **d**, the box limits indicate the 25th and 75th percentiles, the whiskers indicate the full range of the data, and the center line indicates the median. Individual data points are plotted. All the experiments were repeated three times, each giving similar results. Source data

To determine the function of BISP in rice, we ectopically expressed BISP (without its signal peptide) in BPH-susceptible *N14* rice (*N14–Bisp*) plants (Fig. 2a). The stature of *N14–Bisp* plants was similar to control *N14* plants (Extended Data Fig. 3a,b). However, *N14–Bisp* plants were hypersensitive to BPHs, displayed more severe symptoms after BPH feeding and died more quickly than the BPH-infested *N14* plants (Fig. 2b,c). Moreover, the weight gain and honeydew excretion of BPHs feeding on *N14–Bisp* plants were significantly higher than those feeding on *N14* plants (Extended Data Fig. 3c,d). A two-host choice test showed that BPHs preferentially settled on *N14–Bisp* plants rather than *N14* plants (Extended Data Fig. 3e). These results show that BISP increases the susceptibility of rice to BPHs.

We reasoned that the BISP effector was acting as a virulence factor that suppresses rice defence responses, which makes susceptible plants more vulnerable to BPHs. The endogenous levels of free salicylic acid (SA), a plant hormone vital for BPH resistance in rice^10,11,19^, were lower in *N14–Bisp* plants than in *N14* plants (Fig. 2d). Similarly, the levels of SA biosynthesis and signalling genes (Os*ICS1* and Os*NPR1*) and downstream defence-related genes (Os*PR1a*, Os*PR1b*, Os*PR5* and Os*PR10*) transcripts were all significantly lower in *N14–Bisp* plants than in *N14* plants (Extended Data Fig. 3f).

**Extended Data Fig. 4:**
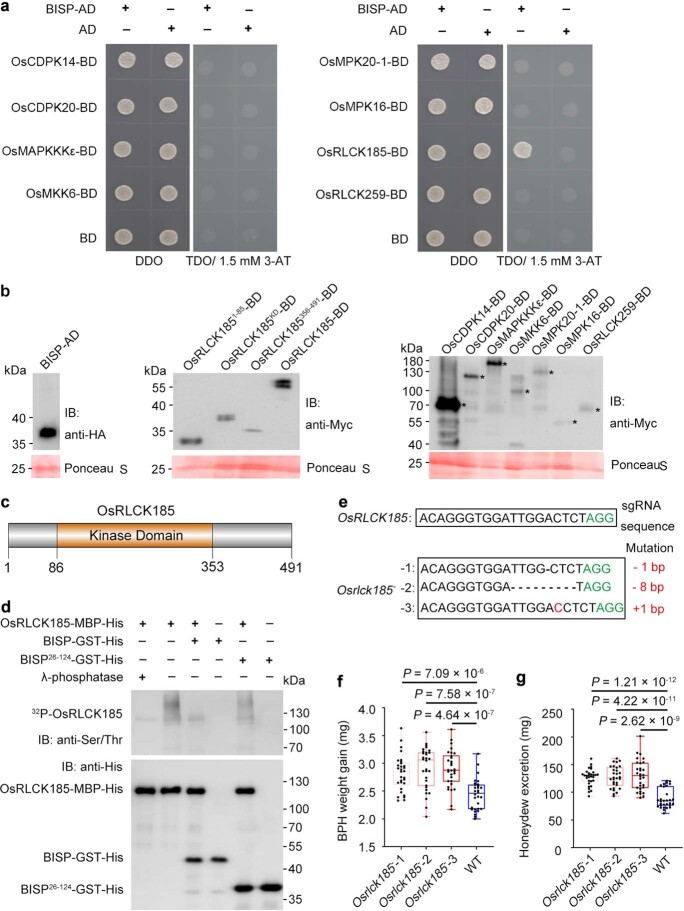
Identification of OsRLCK185 and its role in plant defense. **a**, Y2H screen for BISP-interacting kinases. Yeast strain AH109 was co-transformed with the indicated constructs and grown on DDO (SD/-Leu-Trp) and TDO (SD/-Leu-Trp-His) selective medium containing 1.5 mM 3-amino-1,2,4-triazole (3-AT). **b**, Immunoblot detection of proteins expressed in yeast using anti-HA and anti-Myc (anti-MYC) antibodies, respectively. Ponceau S staining was used as the loading control. Asterisks indicate specific bands detected by immunoblotting analysis. **c**, Structure of *Os*RLCK185. The orange box indicates the kinase domain. **d**, BISP but not BISP^26–124^ (BISP(26–124)) reduced OsRLCK185 autophosphorylation activity at Ser and Thr residues *in vitro*. OsRLCK185 proteins were immunoblotted and phosphorylated OsRLCK185 was detected with anti-phosphoserine/ phosphothreonine antibodies. The input proteins were immunoblotted and detected with anti-His antibodies. **e**, Verification of three independent Os*RLCK185* knockout lines (Os*rlck185*, *Osrlck185*^*−*^-1, −2, and −3) by PCR-based sequencing. **f**,**g**, Weight gain (**f**) and honeydew excretion (**g**) of BPHs on three *Osrlck185*^*−*^ lines and WT plants for 2 d. *P* values were derived by one-way ANOVA (*n* = 30, biologically independent samples). In box plots, the box limits indicate the 25th and 75th percentiles, the whiskers indicate the full range of the data, and the centre line indicates the median. Individual data points are plotted. Experiments were repeated three times (**a**,**b**,**d**,**f**,**g**) or two times (**e**), each giving similar results. Source data

Receptor-like kinases (RLKs) play crucial roles in plant defence against pathogens and herbivores^1,20^. We aimed to identify the target of BISP and to elucidate the molecular mechanism that underlies its ability to suppress defence responses in susceptible plants. To that end, we examined the interactions of RLKs, receptor-like cytoplasmic kinases (RLCKs) and MAP kinases with BISP in Y2H assays (Fig. 2e and Extended Data Fig. 4a–c). Among the eight kinases tested, only *Os*RLCK185 interacted with BISP. BISP interacted with the *Os*RLCK185 kinase domain (86–353 amino acids). The association of *Os*RLCK185 with BISP was confirmed by co-IP in rice protoplasts (Fig. 2f). As *Os*RLCK185 regulates plant immunity through autophosphorylation^21,22^, an in vitro phosphorylation assay and immunoblotting with phosphoserine/phosphothreonine-specific antibodies confirmed that *Os*RLCK185 was an active kinase with autophosphorylation activity (Extended Data Fig. 4d). Furthermore, *Os*RLCK185 autophosphorylation was attenuated by BISP (Fig. 2g and Extended Data Fig. 4d).

We used three *Os*RLCK185 knockout rice lines (Os*rlck185*-1–Os*rlck185*-3 plants) to further explore the roles of *Os*RLCK185 in rice defence against BPHs (Extended Data Fig. 4e). Os*rlck185* lines were more susceptible to BPHs, as they were severely damaged compared with wild-type (WT) plants 4 days after BPH infestation (Fig. 2h,i). BPHs that fed on the Os*rlck185* lines gained significantly more weight and excreted significantly more honeydew than those fed on WT plants (Extended Data Fig. 4f,g), which indicated that *Os*RLCK185 positively regulates basal immunity in BPH-susceptible plants.

To further assess the function of BISP in BPH, we analysed the performance of BPHs treated with *Bisp*-RNAi or *GFP*-RNAi on Os*rlck185*-1 and WT plants. *Bisp*-RNAi insects showed significantly lower weight gain, honeydew excretion and survival rate than *GFP*-RNAi insects on Os*rlck185*-1 plants (Extended Data Fig. 2e–g). This result suggests that BISP has a specific function related to *Os*RLCK185 and may target other defence-related proteins in rice.

## BISP activates BPH14 resistance

**Fig. 3:**
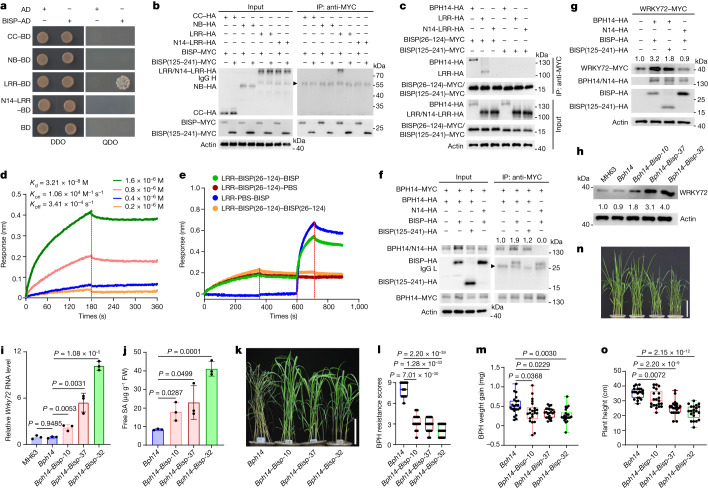
BISP interacts with the BPH14 LRR domain and activates resistance signalling. **a**,**b**, BISP interacted with the LRR domain of BPH14 in Y2H (**a**) and co-IP (**b**) assays. N14–LRR and BISP(125–241) served as negative controls. **c**, BISP(26–124) interacted with BPH14 and its LRR domain in a co-IP assay. N14–LRR served as a negative control. **d**, BLI analysis for binding kinetics between LRR (BPH14–LRR) and BISP. BLI response profile for BISP at different concentrations with sensor-immobilized LRR. *K*_d_, equilibrium dissociation constant; *K*_on_, association rate constant; *K*_off_, dissociation rate constant. **e**, BLI analysis for competitive binding between BISP and BISP(26–124) with LRR. **f**, BISP increased the levels of BPH14 homomeric complex in a co-IP assay in rice protoplasts. BISP(125–241) and N14 served as negative controls. Numbers above the lanes indicate band intensity relative to co-precipitated BPH14–MYC, quantified using ImageJ. **g**, Co-expression of BPH14 and BISP increased WRKY72 levels. **h**,**i**, Protein (**h**) and relative transcript (**i**) levels of *Wrky72* were increased in non-infested *Bph14–Bisp* lines compared with *Bph14* and MH63 plants. **j**, Endogenous free SA levels in non-infested *Bph14–Bisp* and *Bph14* plants. **k**,**l**, Phenotypes (**k**) and BPH-resistance scores (**l**) of *Bph14–Bisp* and *Bph14* plants after BPH infestation for 14 days. *n* = 36 plants examined over 3 independent experiments. **m**, Weight gain of BPHs feeding on *Bph14–Bisp* and *Bph14* plants for 48 h (*n* = 22, biologically independent samples). **n**,**o**, Photographs (**n**) and plant heights (**o**, *n* = 21 plants) of non-infested *Bph14–Bisp* and *Bph14* plants at the 4-leaf stage. In box plots in **l**, **m** and **o**, the box limits indicate the 25th and 75th percentiles, the whiskers indicate the full range of the data and the centre line indicates the median. Individual data points are plotted. In **g** and **h**, numbers above/under the lanes indicate band intensity relative to actin (loading control) quantified using ImageJ. In **h**, **i** and **j**, data are the mean ± s.d. (*n* = 3, biologically independent experiments). In **i**, **j**, **l**, **m** and **o**, *P* values were derived by one-way ANOVA. Similar results were obtained from two (**d**,**e**) or three (**a**–**c**,**f–h**,**k**,**m**–**o**) independently replicated experiments. Scale bars, 10 cm (**k**,**n**). Source data

**Extended Data Fig. 5:**
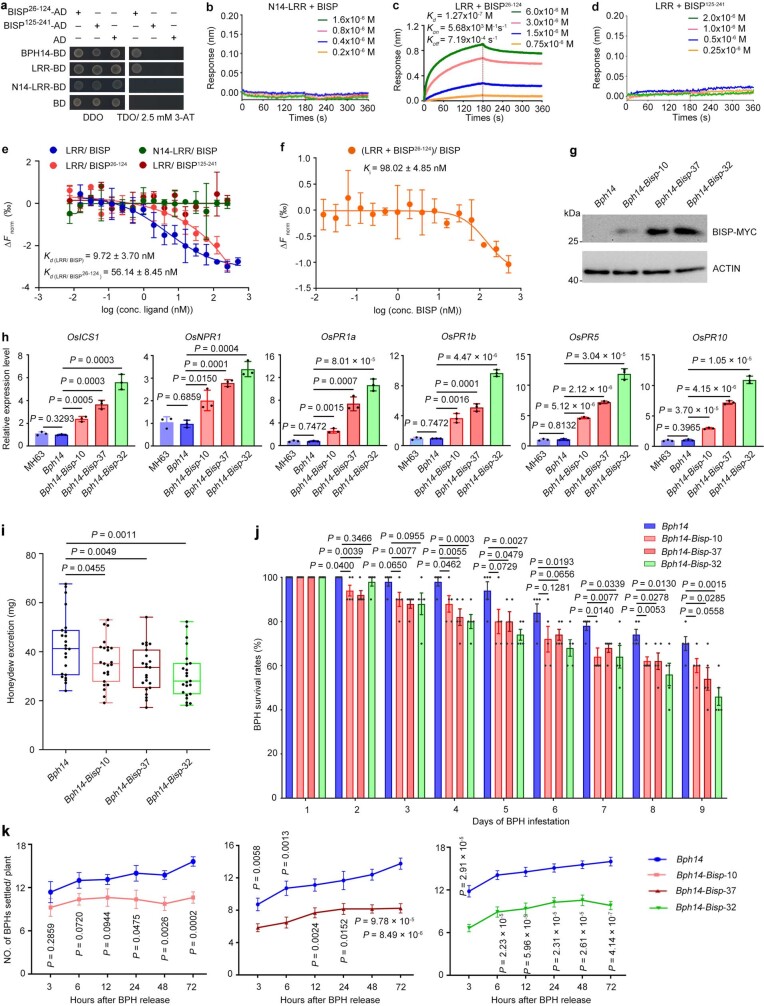
BISP triggered *Bph14*-mediated BPH resistance in *Bph14* plants. **a**, Y2H assays between the full-length BPH14, the BPH14 LRR domain (LRR) and the N14 LRR domain (N14-LRR) and the BISP deletion mutants BISP^26–124^ (BISP(26–124)) and BISP^125–241^(BISP(125–241)). DDO, SD/-Leu-Trp; TDO, SD/-Leu-Trp-His with 2.5 mM 3-AT. **b**, Biolayer interferometry (BLI) determination of the binding kinetics between N14-LRR and BISP. BLI response profile of BISP at different concentrations with the sensor-immobilized N14-LRR. **c**, BLI analysis for the binding kinetics between the BPH14 LRR and BISP^26–124^. BLI response profile of BISP^26–124^ at different concentrations with the sensor-immobilized LRR. *K*_*d*_, equilibrium dissociation constant; *K*_*on*_, association rate constant; *K*_*off*_, dissociation rate constant. **d**, BLI analysis for the binding kinetics between the BPH14 LRR and BISP^125–241^. BLI response profile of BISP^125–241^ at different concentrations with the sensor-immobilized LRR. **e**, MST analysis of binding affinity between fluorescently labeled LRR, N14-LRR and BISP, BISP^26–124^ and BISP^125–241^. The *K*_*d*_ values show the binding between LRR and BISP, BISP^26–124^. Δ*F*_norm_ is the normalized fluorescence minus the relative fluorescence of the unbound state. Data are mean ± s.d. (*n* = 3, biologically independent experiments). **f**, MST analysis of the competitive binding between BISP and BISP^26–124^ with LRR. Δ*F*_norm_ is the normalized fluorescence minus the relative fluorescence of the unbound state. Data are mean ± s.d. (*n* = 3, biologically independent experiments). **g**, BISP protein levels in the independent *Bph14*-*Bisp* homozygous T_2_ transgenic lines and *Bph14* control plants. Total proteins were immunoblotted and detected with anti-Myc and anti-ACTIN antibodies, respectively. **h**, Relative transcript levels of *Bph14*-activated SA biosynthesis- and signaling-related genes *OsICS1* and *OsNPR1*, and defense-related genes *OsPR1a*, *OsPR1b*, *OsPR5* and *OsPR10* in the *Bph14*-*Bisp* transgenic lines and WT *Bph14* plants. *P* values were derived by one-way ANOVA. Data are mean ± s.d. (*n* = 3, biologically independent experiments). **i**, Honeydew excretion by BPHs reared on the *Bph14*-*Bisp* transgenic lines and WT *Bph14* plants for 48 h. *P* values were derived by one-way ANOVA (*n* = 20, biologically independent plants). In box plots, the box limits indicate the 25th and 75th percentiles, the whiskers indicate the full range of the data, and the center line indicates the median. Individual data points are plotted. **j**, Survival rate of BPHs feeding on the *Bph14*-*Bisp* transgenic lines and WT *Bph14* plants for 9 days. *P* values were derived by one-way ANOVA. Data are mean ± s.e.m. (*n* = 5, biologically independent experiments). **k**, Two-host choice test of BPHs on the *Bph14*-*Bisp* transgenic plants and WT *Bph14* plants. *P* values were derived by one-way ANOVA. Data are mean ± s.e.m. (*n* = 8, biologically independent experiments). All the experiments were repeated two (**b**–**d**) or three (**a**,**g**,**i**–**k**) times, each giving similar results. Source data

Pathogen effectors that are directly or indirectly recognized by NLR proteins trigger ETI^13,14^. Y2H and co-IP assays showed that BISP did not interact with either the CC or NB domain, but interacted with the LRR domain of BPH14 (Fig. 3a,b). Furthermore, the N terminus of BISP (amino acids 26–124) interacted with the LRR domain (Fig. 3c and Extended Data Fig. 5a). Biolayer interferometry (BLI) assays using the BPH14–LRR or N14–LRR domains and BISP purified from insect cells showed that BPH14–LRR bound to BISP with a dissociation constant (*K*_d_) of 3.21 × 10^−8^ M (Fig. 3d) and N14–LRR binding to BISP was not detected (Extended Data Fig. 5b). BPH14–LRR also bound to BISP(26–124) with a *K*_d_ of 1.27 × 10^−7^ M (Extended Data Fig. 5c), but not bind to BISP(125–241) (Extended Data Fig. 5d). Binding competition experiments showed that BISP competed with BISP(26–124) for binding to BPH14–LRR (Fig. 3e). Microscale thermophoresis (MST) assays confirmed that BISP directly bound to BPH14 (Extended Data Fig. 5e,f).

BPH14 forms a homomeric complex and interacts with WRKY72 to increase WRKY72 accumulation, which in turn activates downstream defence signalling^19^. To examine the effects of BISP on the formation of BPH14 complexes and on WRKY72 protein levels, we co-expressed BPH14 and BISP in rice protoplasts. The levels of BPH14 self-association (Fig. 3f) and WRKY72 levels (Fig. 3g) were increased when BPH14 and BISP were co-expressed in rice protoplasts, which suggested that BISP stimulated the activity of BPH14.

To examine the activation of BPH14 by BISP, we constructed transgenic *Bph14–Bisp* rice lines in which *Bisp* was constitutively expressed in the *Bph14* background (Extended Data Fig. 5g). WRKY72 protein and transcript levels were higher in three homozygous *Bph14–Bisp* plants relative to *Bph14* plants (Fig. 3h,i). In addition, non-infested *Bph14–Bisp* plants had higher levels of free SA than *Bph14* plants (Fig. 3j). Furthermore, the SA-responsive Os*ICS1* and Os*NPR1* and four rice *PR* genes were significantly upregulated (Extended Data Fig. 5h). The intensity of *Bph14*-mediated resistance responses in the *Bph14–Bisp* plants positively correlated with the levels of BISP protein.

We evaluated BPH resistance in *Bph14–Bisp* plants. Whereas *Bph14* plants showed little damage after 7 days of infestation (Fig. 1a), prolonged BPH feeding (14 days) caused substantial damage. By contrast, prolonged BPH feeding caused little damage to *Bph14–Bisp* plants (Fig. 3k,l), which suggested that ectopic expression of *Bisp* in *Bph14* plants enhances resistance to BPHs. In addition, BPH adults preferred *Bph14* over *Bph14–Bisp* plants in two-host choice assays and had poor performance on *Bph14–Bisp* plants based on BPH weight gain, honeydew excretion and survival (Fig. 3m and Extended Data Fig. 5i–k).

We further tested the performance of *Bisp*-RNAi and *GFP*-RNAi insects on *Bph14* plants and susceptible control MH63 plants (Extended Data Fig. 2h–j). The *GFP*-RNAi and non-injected insects exhibited significantly decreased weight gain, honeydew excretion and survival on *Bph14* plants compared with those on MH63 plants. By contrast, the *Bisp*-RNAi insects showed similar weight gain, honeydew excretion and survival rate when fed on *Bph14* or MH63 plants. Moreover, the *Bisp*-RNAi insects exhibited more weight gain, honeydew excretion and a higher survival rate than *GFP*-RNAi insects on *Bph14* plants. These results show that the knockdown of *Bisp* improved BPH performance on *Bph14* plants.

**Extended Data Fig. 6:**
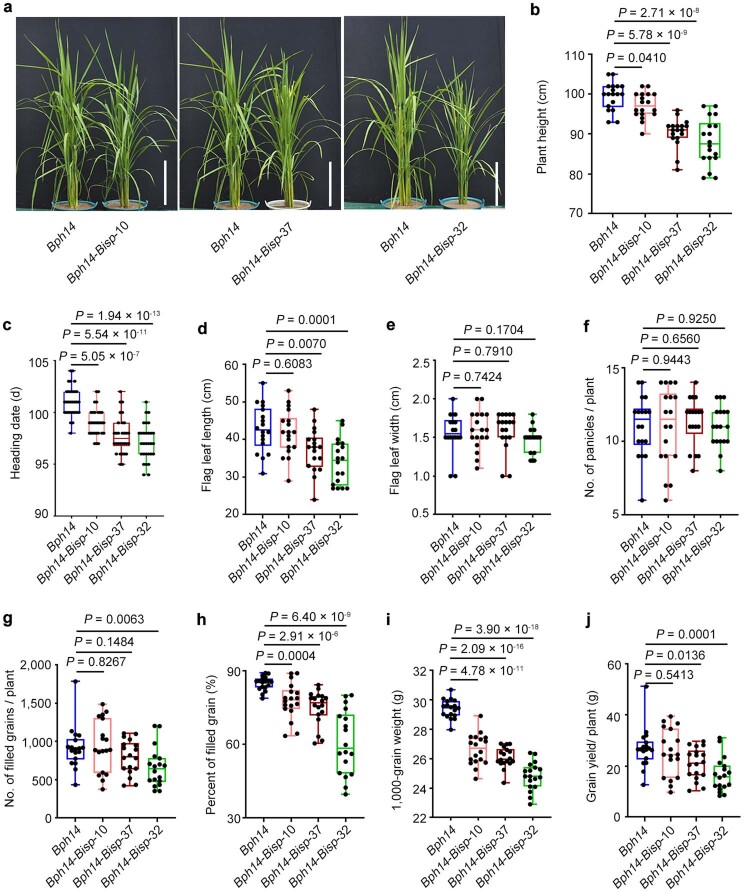
Agronomic traits of the *Bph14*-*Bisp* transgenic lines and WT *Bph14* plants. **a**, Phenotypes of the *Bph14*-*Bisp* transgenic lines and *Bph14* plants at the heading stage. Scale bar, 30 cm. **b**, **c**, Plant height (**b**) and heading date (**c**) of the *Bph14*-*Bisp* transgenic lines and *Bph14* plants at the heading stage. *P* values were derived by one-way ANOVA. For **b**, *n* = 18, biologically independent samples. For **c**, *n* = 30, biologically independent samples. **d**–**j**, Agronomic traits of the *Bph14*-*Bisp* transgenic lines and WT *Bph14* plants at the mature stage. (**d**), Flag leaf length. (**e**), Flag leaf width. (**f**), The number of panicles per plant. (**g**), The number of filled grains per plant. (**h**), Percentage of filled grains. (**i**), 1,000-grain weight. (**j**), Grain yield per plant. One-way ANOVA (*n* = 18, biologically independent samples). In box plots in **b**–**j**, the box limits indicate the 25th and 75th percentiles, the whiskers indicate the full range of the data, and the center line indicates the median. Individual data points are plotted. Source data

Constitutive activation of immune responses to pathogens usually negatively affects plant growth^23,24^. We observed that ectopic expression of *Bisp* in the *Bph14* background had significant fitness costs. *Bph14*–*Bisp* plants were smaller than *Bph14* plants at both the seedling and heading stages (Fig. 3n,o and Extended Data Fig. 6a,b). Furthermore, the heading dates of *Bph14*–*Bisp* plants were advanced (Extended Data Fig. 6c). Finally, *Bph14*–*Bisp* plants exhibited poor agronomic traits, with significantly lower yields than *Bph14* plants (Extended Data Fig. 6d–j). The degree of fitness costs in *Bph14*–*Bisp* plants positively correlated with the levels of BISP and the intensity of *Bph14*-mediated resistance responses (Fig. 3h–m and Extended Data Figs. 5g–k and 6). These results indicate that constitutively expressed BISP induces BPH14-mediated resistance in *Bph14*–*Bisp* plants, which has a substantial fitness cost in rice.

## BPH14 mediates BISP turnover by autophagy

**Fig. 4:**
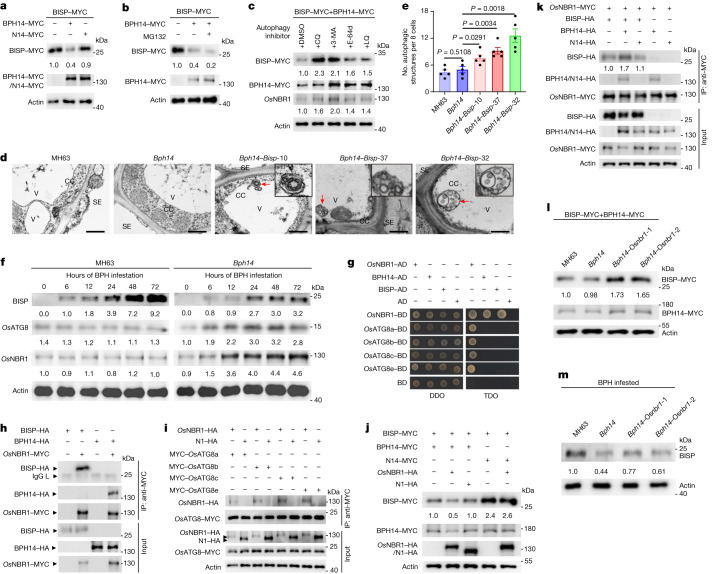
BISP is degraded through *Os*NBR1-mediated autophagy. **a**, Effects of BPH14 and N14 on BISP levels in rice protoplasts. **b**, Effects of the 26S proteasome inhibitor MG132 on BISP levels in rice protoplasts. **c**, Effects of autophagy inhibitors on BISP protein levels in rice protoplasts. 3-MA, 3-methyladenine; CQ, chloroquine; E-64d, aloxistatin; LQ, leupeptin. DMSO was the solvent for all inhibitors. **d**, Transmission electron microscopy images of autophagic structures in the phloem of non-infested *Bph14–Bisp* plants. Insets show enlarged autophagosome image at higher magnification. Red arrows indicate the location of double-membrane autophagosomes. CC, companion cell; SE, sieve element cell; V, vacuole. Scale bars, 500 nm. **e**, Quantification of double-membraned autophagosomes. *P* values were derived by one-way ANOVA. Data are the mean ± s.e.m. (*n* = 5, biologically independent experiments with every 8 cells as a biological replicate). **f**, Immunoblot detection of BISP, *Os*ATG8 and *Os*NBR1 in *Bph14* and MH63 plants. **g**, Y2H assay of the interactions between BISP, BPH14, *Os*NBR1 and four *Os*ATG8 proteins. **h**, Co-IP assays of interactions between *Os*NBR1 and BISP or BPH14 in rice protoplasts. **i**, Co-IP assay of interactions between *Os*NBR1 and four *Os*ATG8 proteins in rice protoplasts. The *Os*NBR1 mutant N1 served as a negative control. **j**, Effects of *Os*NBR1 and BPH14 on BISP levels in rice protoplasts. N14 and N1 served as negative controls. **k**, BPH14 enhanced interactions between *Os*NBR1 and BISP in rice protoplasts. N14 served as a negative control. Numbers under the lanes indicate band intensity relative to co-precipitated *Os*NBR1–MYC, quantified using ImageJ. **l**, Immunoblot detection of BISP in protoplasts of MH63, *Bph14* and *Os*NBR1 knockout (Os*nbr1*) plants. **m**, Immunoblot detection of BISP in MH63, *Bph14* and Os*nbr1* plants after BPH infestation. Numbers under the lanes (**a**–**c**,**f**,**j**,**l**,**m**) indicate protein abundance relative to that of actin (loading control), quantified using ImageJ. Experiments (**a**–**d**,**f**–**m**) were repeated three times and gave similar results. Source data

**Extended Data Fig. 7:**
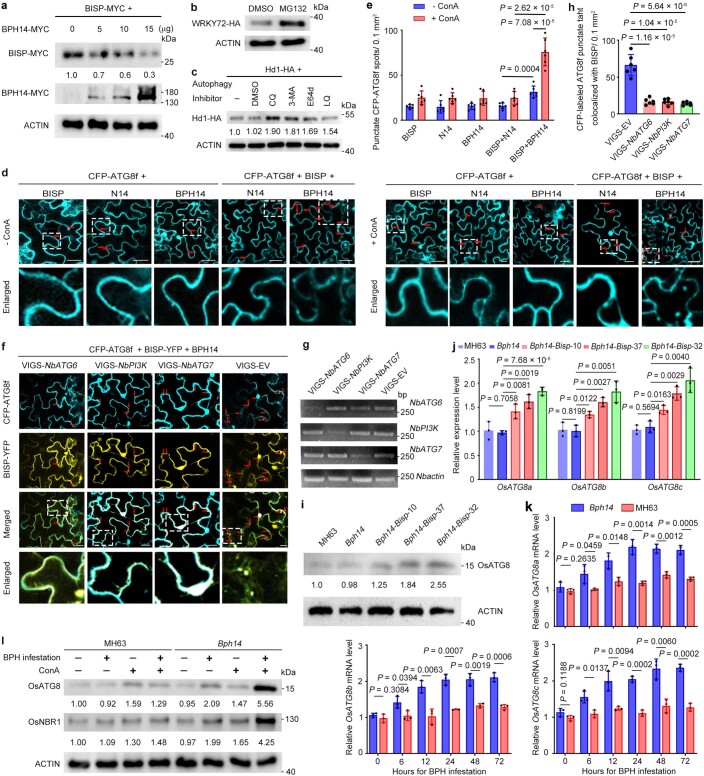
The autophagic degradation of BISP was promoted by BPH14. **a**, Dosage effect of BPH14 on BISP levels in rice protoplasts. BISP-MYC was expressed with or without BPH14-MYC in rice protoplasts. Total proteins were immunoblotted and detected with anti-Myc and anti-ACTIN antibodies, respectively. **b**, Immunoblots showing that proteasome degradation of WRKY72-HA was blocked by MG132 treatment. Total protoplast proteins were immunoblotted and detected with anti-HA and anti-ACTIN antibodies, respectively. **c**, Immunoblots showing that the autophagy degradation of Hd1-HA was blocked by treatments with autophagy inhibitors. Total protoplast proteins were immunoblotted and detected with anti-HA and anti-ACTIN antibodies, respectively. **d**, (*Upper*) Confocal images of CFP-ATG8f-labeled autophagic puncta structures in the absence (*left row*) or presence (*right row*) of ConA. Autophagosomes in cells co-expressing CFP-ATG8f with BISP, N14, or BPH14 (individually) and CFP-ATG8f and BISP with N14 or BPH14 after agroinfiltration of *N. benthamiana* leaves are displayed. Red arrows indicate CFP-ATG8f-labeled autophagic puncta. (*Bottom*) Insets show enlarged autophagosome image at higher magnification. Scale bars, 25 μm. **e**, Average number of CFP-NbATG8-labeled autophagic puncta in the presence or absence ConA. *P* values were derived by one-way ANOVA. Data are mean ± s.d. (*n* = 6, biologically independent experiments). **f**,**g**,**h**, Silencing of *NbATG6*, *NbPI3K* or *NbATG7* suppressed the production of CFP-ATG8f-labeled autophagic puncta. (**f**), Confocal images of CFP-ATG8f-labeled autophagic puncta structures co-localized with BISP-YFP. CFP-ATG8f, BISP-YFP and BPH14 were transiently co-expressed in the silenced leaves (VIGS-*NbATG6*, VIGS-*NbPI3K* and VIGS-*NbATG7*) or the non-silenced control leaves (VIGS-EV). Red arrows indicate CFP-ATG8f-labeled autophagic puncta co-localized with BISP-YFP. (*Bottom*) Insets show enlarged autophagosome image at higher magnification. Scale bars, 25 μm. (**g**), The transcript levels of *NbATG6*, *NbPI3K* and *NbATG7* by semi-quantitative RT-PCR. The transcript levels of *NbACTB* were used as a loading control. (**h**), Average number of CFP-NbATG8-labeled autophagic puncta in the silenced and control leaves. *P* values were derived by one-way ANOVA. Data are mean ± s.d. (*n* = 6, biologically independent experiments). **i**, Immunoblot detection of OsATG8 in the non-infested *Bph14*-*Bisp* transgenic lines, *Bph14* and MH63 plants. Total proteins were immunoblotted and detected with anti-AtATG8A and anti-ACTIN antibodies, respectively. **j**, Relative transcript levels of *OsATG8s* (*OsATG8a, OsATG8b, OsATG8c*) in the non-infested *Bph14*-*Bisp* transgenic lines, *Bph14* and MH63 plants. *P* values were derived by one-way ANOVA. Data are mean ± s.d. (*n* = 3, biologically independent experiments). **k**, Relative transcript levels of *OsATG8s* (*OsATG8a, OsATG8b, OsATG8c*) in *Bph14* and MH63 plants after BPH feeding for different durations. *P* values were derived by one-way ANOVA. Data are mean ± s.d. (*n* = 3, biologically independent experiments). **l**, Immunoblot detection of OsATG8 and OsNBR1 in the non-infested and BPH-infested MH63 and *Bph14* plants in the absence or presence of ConA treatment. Leaf sheaths total proteins were immunoblotted and detected with anti-AtATG8A, anti-AtNBR1 and anti-ACTIN antibodies, respectively. Numbers under the lanes (**a**,**c**,**i**,**l**) indicate protein abundance relative to that of ACTIN, quantified by ImageJ. Experiments (**a**–**d**,**f**,**g**,**i**,**l**) were repeated three times, each giving similar results. Source data

The fitness costs imposed by ectopic expression of BISP in *Bph14* plants suggested that the activation of *Bph14*-mediated resistance should be tightly controlled in the natural habitats of rice. Several lines of evidence support this hypothesis. First, when BISP is expressed in rice protoplasts, BISP levels were lower when BISP was co-expressed with BPH14 (Fig. 4a), and BISP levels negatively correlated with BPH14 levels (Extended Data Fig. 7a). Indicative of the specificity of the BISP–BPH14 interaction, BISP levels were not reduced when BISP was co-expressed with N14 (Fig. 4a). These results indicate that BISP is degraded in a BPH14-dependent manner.

The ubiquitin–proteasome system and autophagy are the main protein-degradation pathways in plant cells^25^. The ubiquitin–proteasome system is a key post-translational mechanism for controlling plant immune responses to pathogens^26^. To test whether the ubiquitin–proteasome system is involved in the modulation of BISP–BPH14 immune activation, the 26S proteasome inhibitor MG132 was added to protoplasts expressing BISP and BPH14. MG132 blocked the proteasome pathway degradation of WRKY72 (Extended Data Fig. 7b), but did not inhibit the degradation of BISP (Fig. 4b). By contrast, treatment with four autophagy inhibitors blocked the degradation of BISP (Fig. 4c and Extended Data Fig. 7c), which suggested that the autophagy pathway is involved in the degradation of BISP.

To further assess the role of autophagy in BISP turnover, we used cyan fluorescent protein-tagged ATG8F (CFP–ATG8F) as a marker^27,28^ to monitor autophagy (Extended Data Fig. 7d,e). When CFP–ATG8F was expressed with BISP or BPH14 individually in *Nicotiana benthamiana* leaves (hereafter *Nb* is used to denote *N.* *benthamiana*-related proteins or genes), few punctate CFP structures, corresponding to pre-autophagosomes and autophagosomes, were detected. However, when CFP–ATG8F was expressed with BISP and BPH14, the number of punctate CFP structures significantly increased, which indicated that BISP and BPH14 co-expression induced autophagy. This interaction was specific, as expressing CFP–ATG8F with BISP and N14 did not increase the number of autophagosomes. Concanamycin A (ConA) blocks autophagic flux^28^, and the numbers of autophagic bodies were substantially increased in cells that expressed both BISP and BPH14 and treated with ConA. When we silenced the core autophagy genes Nb*ATG6*, Nb*PI3K* and Nb*ATG7*, the numbers of autophagic bodies were markedly decreased in the silenced leaves that expressed CFP–ATG8F with BISP–yellow fluorescent protein (YFP) and BPH14 (Extended Data Fig. 7f–h). Furthermore, using transmission electron microscopy, we observed more double-membraned autophagosome structures in the phloem companion cells of non-infested *Bph14–Bisp* plants than in *Bph14* and MH63 plants (Fig. 4d,e). Accordingly, *Os*ATG8 protein levels and Os*ATG8a*, Os*ATG8b* and Os*ATG8c* transcript levels were significantly higher in non-infested *Bph14*–*Bisp* plants than in *Bph14* and MH63 plants (Extended Data Fig. 7i,j). These observations indicate that BISP is degraded through autophagy in a BPH14-dependent manner.

As BPHs secrete saliva containing BISP into rice tissues when feeding (Fig. 1j,k and Extended Data Fig. 1q–t), we monitored BISP levels in infested BPH-susceptible (MH63) or BPH-resistant (*Bph14*) plants. In MH63 plants, BISP was first detected at 6 h and increased continuously for 72 h of BPH feeding (Fig. 4f). By contrast, BISP levels were lower in *Bph14* plants, with levels sustained after 24 h of feeding (Fig. 4f). *Os*ATG8 levels did not significantly change in BPH-infested susceptible plants. By contrast, in *Bph14* plants, *Os*ATG8 levels increased by 6 h after feeding and were maintained for 72 h (Fig. 4f). These results suggest that autophagy is activated in *Bph14* plants but not in BPH-susceptible rice. These data correlated well with the upregulation of three Os*ATG8* genes in *Bph14* plants compared with MH63 plants following BPH infestation (Extended Data Fig. 7k). ConA treatment substantially increased levels of ATG8 proteins in BPH-infested *Bph14* plants (Extended Data Fig. 7l). These results indicate that BPH feeding activates autophagy and that BISP levels are tightly controlled in *Bph14* plants.

## *Os*NBR1-mediated autophagy controls BISP levels

**Extended Data Fig. 8:**
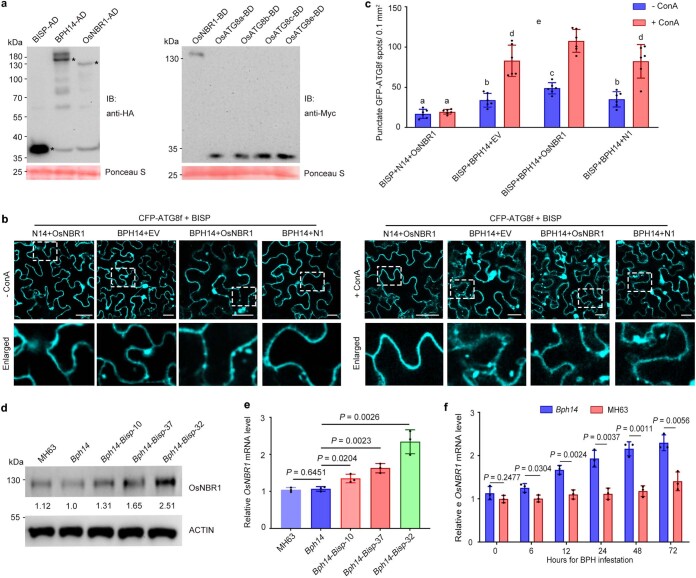
OsNBR1 played a role in the autophagic degradation of BISP by BPH14. **a**, Immunoblot detection of proteins expressed in yeast using anti-HA and anti-Myc antibodies, respectively. Ponceau S staining was used as the loading control. Asterisks indicate specific bands detected by immunoblotting analysis. **b**, Confocal images of CFP-NbATG8f-labeled autophagic puncta structures in the absence (*left*) or presence (*right*) of ConA. The photos showing cells co-expressing CFP-NbATG8f and BISP with N14 and OsNBR1, BPH14, BPH14 and OsNBR1, or BPH14 and OsNBR1 mutant N1 after agroinfiltration of *N. benthamiana* leaves. Red arrows indicate CFP-NbATG8f-labeled autophagic puncta. (*Bottom Row*) Insets show enlarged autophagosome image at higher magnification. EV, Empty Vector. Scale bars, 25 μm. **c**, Average number of CFP-NbATG8-labeled autophagic puncta in the absence or presence of ConA. Data are mean ± s.d. (*n* = 10, biologically independent samples). Different letters indicate significant differences (*P* < 0.05, one-way ANOVA); exact P values are provided in Supplementary Table 4. **d**, Immunoblot detection of OsNBR1 levels in the non-infested *Bph14*-*Bisp* transgenic lines, *Bph14* and MH63 plants. Total proteins were immunoblotted and detected with anti-AtNBR1 and anti-ACTIN antibodies, respectively. Numbers under the lanes indicate protein abundance relative to that of ACTIN, quantified by ImageJ. **e**, Relative *OsNBR1* transcript levels in the non-infested *Bph14*-*Bisp* transgenic lines, *Bph14* and MH63 plants. *P* values were derived by one-way ANOVA. Data are means ± s.d. (*n* = 3, biologically independent experiments). **f**, Relative *OsNBR1* transcript levels in *Bph14* and MH63 plants after BPH infestation. *P* values were derived by one-way ANOVA. Data are mean ± s.d. (*n* = 3, biologically independent experiments). Experiments (**a**,**b**,**d**) were repeated at least three times, each giving similar results. Source data

Mechanisms of autophagy are conserved in yeast, plants and metazoans^29^. In rice and other plants, multiple ATG8 proteins mediate autophagy^30–32^. To clarify the molecular mechanisms that underlie the BPH14-dependent degradation of BISP, we examined the interactions of four *Os*ATG8 proteins with BISP or BPH14 using Y2H assays. Neither BISP nor BPH14 interacted with the *Os*ATG8 proteins (Fig. 4g and Extended Data Fig. 8a).

In addition to canonical autophagy, selective autophagy targets specific cargoes, such as intracellular pathogens and defective proteins, and plays pivotal roles in eukaryotic organisms^33–35^. NBR1 is a selective autophagy cargo receptor and interacts with ATG8 proteins to facilitate the autophagy-dependent degradation of target proteins^33–37^. We therefore examined the interaction of BISP or BPH14 with *Os*NBR1 in Y2H assays. Both BISP and BPH14 interacted with *Os*NBR1 (Fig. 4g and Extended Data Fig. 8a). Co-IP assays in rice protoplasts confirmed these interactions (Fig. 4h). Furthermore, *Os*NBR1 interacted with all four *Os*ATG8 proteins in Y2H assays (Fig. 4g and Extended Data Fig. 8a) and in rice protoplasts (Fig. 4i), which suggested that *Os*NBR1 might mediate the degradation of BISP.

**Extended Data Fig. 9:**
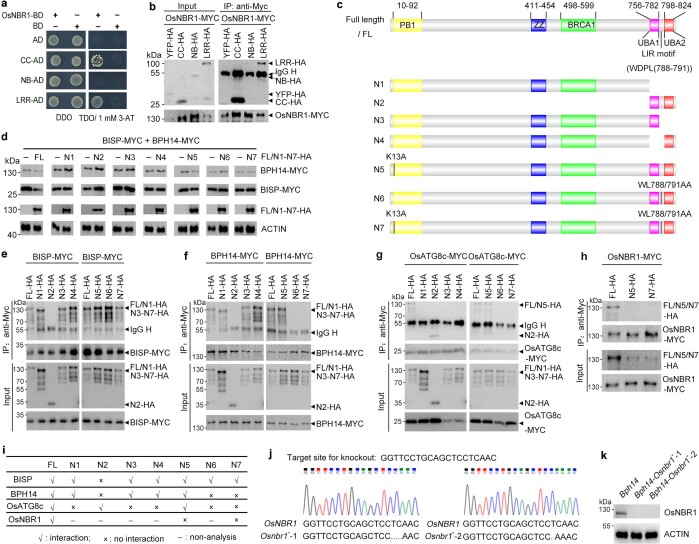
The PB1 domain, UBA domain and LIR motif of OsNBR1 played essential roles in BPH14-promoted autophagic degradation of BISP. **a**, Y2H assay of the interactions between OsNBR1 and the CC, NB, and LRR domains of BPH14. DDO, SD/-Leu-Trp; TDO, SD/-Leu-Trp-His with 1 mM 3-AT. **b**, Co-IP assay of the interactions between OsNBR1 and the CC, NB, and LRR domains of BPH14 in rice protoplasts. OsNBR1-MYC and CC-HA, NB-HA or LRR-HA were co-expressed in rice protoplasts, and used in the co-IP assay with anti-Myc agarose; the input and immunoprecipitated proteins were detected with anti-HA, anti-Myc or anti-ACTIN antibodies. **c**, Schematic illustration of OsNBR1 and the N1-N7 mutant constructs used in this study. **d**, OsNBR1 mutants were unable to degrade BISP in rice protoplasts. BISP-MYC was co-expressed with BPH14-MYC and the full-length OsNBR1 (FL-HA) or its HA-tagged mutants (N1-N7-HA) in rice protoplasts. The levels of BISP-MYC protein were detected with anti-Myc antibody. **e**–**h**, Co-IP assays of the interactions between the OsNBR1 mutants and BISP (**e**), BPH14 (**f**), OsATG8c **(g**) and OsNBR1 (**h**) in rice protoplasts. BISP-MYC, BPH14-MYC, MYC- OsATG8c, or OsNBR1-MYC was co-expressed with OsNBR1(FL-HA) and its HA-tagged mutants (N1-N7-HA) in rice protoplasts. Proteins interacting with OsNBR1-HA or its mutants were identified in a co-IP assay with anti-MYC agarose; the input and immunoprecipitated proteins were detected with anti-HA, anti-Myc or anti-ACTIN antibodies. **i**, Summary of interactions between the OsNBR1 mutants and BISP, BPH14, OsATG8c, and OsNBR1, respectively. **j**, Verification of the mutations in the independent homozygous *OsNBR1* knockout (*Bph14*-Os*nbr1*, *Osnbr1*^*−*^) lines (*Bph14*-*Osnbr1*^*−*^-1 and *Bph14*-*Osnbr1*^*−*^-2) by PCR-based sequencing. **k**, Immunoblot detection of OsNBR1 proteins in *Bph14* and two *OsNBR1* knockout lines (*Bph14*-*Osnbr1*^*−*^-1 and *Bph14*-*Osnbr1*^*−*^-2). Total proteins were immunoblotted and detected with anti-AtNBR1 and anti-ACTIN antibodies, respectively. In **c**-**i**, FL, full-length of OsNBR1. N1-N7, the seven OsNBR1 mutants. Experiments were repeated three times (**a**,**b**,**d**–**h**,**k**) or two times (**j**) with similar results.

The roles of *Os*NBR1 in the autophagic degradation of BISP were examined by immunoblotting. The expression of BISP with *Os*NBR1 in rice protoplasts did not influence BISP levels. By contrast, the expression of BISP, *Os*NBR1 and BPH14 reduced the level of BISP (Fig. 4j), which suggested that *Os*NBR1 facilitated BPH14-dependent degradation of BISP. We used CFP–ATG8F to determine the effects of *Os*NBR1 on the numbers of autophagic vesicles. The expression of *Os*NBR1 with BISP and BPH14 induced more punctuate CFP–ATG8F structures in *N.* *benthamiana* leaves than expression of only BISP and BPH14 (Extended Data Fig. 8b,c). Additionally, *Os*NBR1 protein and transcript levels were significantly higher in non-infested *Bph14–Bisp* plants than in *Bph14* and MH63 plants (Extended Data Fig. 8d,e). Following BPH infestation, *Os*NBR1 protein and RNA levels were increased in *Bph14* plants, but not in BPH-susceptible MH63 plants (Fig. 4f and Extended Data Fig. 8f). Moreover, ConA treatment significantly increased *Os*NBR1 levels in *Bph14* plants following BPH infestation (Extended Data Fig. 7l). Y2H and co-IP assays showed that the CC and LRR domains of BPH14 interacted with *Os*NBR1 (Extended Data Fig. 9a,b). Co-IP assays also showed that BPH14 enhanced the interaction of *Os*NBR1 with BISP (Fig. 4k).

NBR1 homopolymerizes through its PB1 domain, binds to ubiquitin through its UBA domain and interacts with ATG8 through a conserved LIR motif to promote selective autophagy^37^. *Os*NBR1 contains these interaction domains, as well as conserved ZZ-type zinc finger and BRCA1 domains. To determine which *Os*NBR1 domain (or domains) participates in the degradation of BISP, we generated four truncated forms (N1 to N4) and three site-directed mutants (N5 to N7) of *Os*NBR1 (Extended Data Fig. 9c). Co-expression assays showed that none of mutants were able to degrade BISP (Extended Data Fig. 9d). Whereas the Os*nbr1* N5 mutant lost the ability to form homopolymers (Extended Data Fig. 9h), the other Os*nbr1* mutants retained the ability to form homopolymers. However, these mutants failed to interact with BPH14, BISP or *Os*ATG8C (Extended Data Fig. 9e–i). These results suggest that *Os*NBR1 is involved in BISP degradation through autophagy and that domains outside the UBA in *Os*NBR1 are important for interacting with its target BISP.

We generated *Os*NBR1 knockout mutants in the *Bph14* background (*Bph14–*Os*nbr1*) to further analyse the roles of *Os*NBR1 in the degradation of BISP (Extended Data Fig. 9j). *Os*NBR1 was undetectable in the *Bph14–*Os*nbr1* mutants (Extended Data Fig. 9k). We then expressed BISP and BPH14 in *Bph14–*Os*nbr1*, *Bph14* and MH63 protoplasts. Consistent with a role for *Os*NBR1 in BISP turnover, BISP levels were higher in *Bph14–*Os*nbr1* protoplasts than in *Bph14* and MH63 protoplasts (Fig. 4l). BISP was more abundant in infested *Bph14–*Os*nbr1* plants than in *Bph14* plants, but less abundant than in MH63 plants (Fig. 4m). These results indicate that *Os*NBR1-mediated autophagic degradation controls BISP levels.

## BISP degradation attenuates resistance

On susceptible plants, BPHs consume phloem sap for prolonged times^17^. By contrast, on BPH-resistant plants, various resistance factors prevent sustained phloem ingestion^11,12,16,17,38^. BPHs have a higher frequency of stylet probing, penetrating numerous sites in an attempt to find an acceptable feeding site^17,39^.

**Fig. 5:**
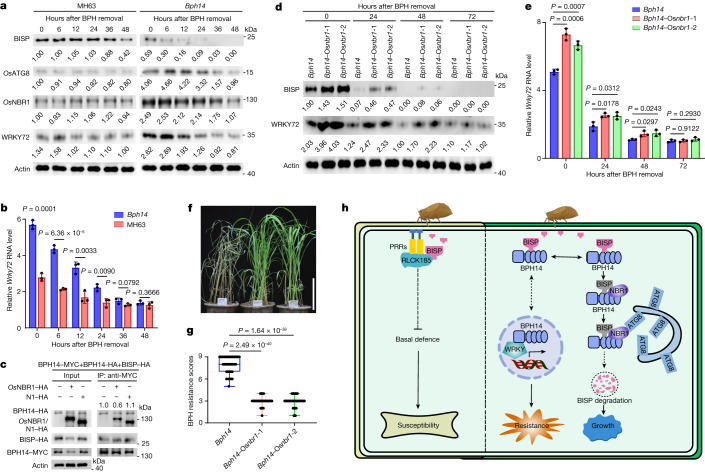
BPH14-mediated BISP degradation contributes to the termination of BPH14 activation. **a**, Immunoblot detection of BISP, *Os*ATG8, *Os*NBR1, and WRKY72 in *Bph14* and MH63 plants after cessation of BPH feeding. **b**, Relative *Wrky72* transcript levels in *Bph14* and MH63 plants after cessation of BPH feeding. **c**, Co-IP detection of levels of BPH14 homomeric complex formation. N1 served as a negative control. Numbers above the lanes indicate band intensity relative to co-precipitated BPH14–MYC, quantified using ImageJ. **d**, Immunoblot detection of BISP and WRKY72 in *Bph14* and *Bph14–*Os*nbr1* plants after cessation of BPH feeding. **e**, Relative *Wrky72* transcript levels in *Bph14* and *Bph14–*Os*nbr1* plants after cessation of BPH feeding. **f**,**g**, Photographs (**f**) and resistance scores (**g**) of *Bph14* and *Bph14–*Os*nbr1* plants after 14 days of BPH infestation. Scale bar, 10 cm. The box limits indicate the 25th and 75th percentiles, the whiskers indicate the full range of the data and the centre line indicates the median. Individual data points are plotted (*n* = 42 plants examined over 3 independent experiments). **h**, A working model for BISP-induced immunity regulation. In BPH-susceptible rice, BISP interacts with *Os*RLCK185 and suppresses its phosphorylation activity, thereby inhibiting basal defence and promoting BPH feeding. In rice carrying the BPH resistance gene *Bph14*, BISP binds directly to the LRR domain of BPH14 and activates HPR. Continuous activation of HPR is detrimental to plant growth and reproduction. Therefore, following activation of BPH14-mediated immunity, BISP–BPH14 binds to *Os*NBR1, which mediates the autophagic degradation of BISP. The degradation of BISP leads to the termination of BPH14-mediated immunity, which restores cellular homeostasis and prevents the fitness costs associated with prolonged activation of BPH14-mediated immunity. The timing of dissociation of the BISP–BPH14 complex after association with *Os*NBR1 and the fate of BPH14 are yet to be determined. Numbers under lanes (**a**,**d**) indicate protein abundance relative to that of actin (loading control, **a**,**c**,**d**), quantified using ImageJ. In **b**, **c**, **e** and **g**, *P* values were derived by one-way ANOVA. In **b** and **e**, data are the mean ± s.d. (*n* = 3, biologically independent experiments). Experiments (**a**,**c**,**d**,**f**) were repeated three times, each giving similar results. PRRs, pattern recognition receptors. Source data

**Extended Data Fig. 10:**
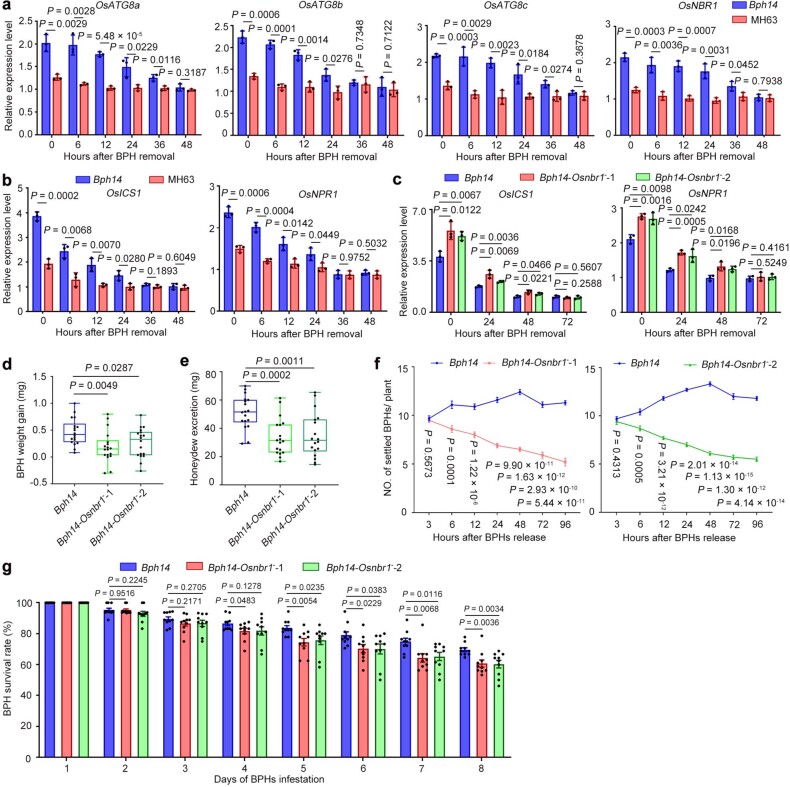
Effects of BPH infestation cessation in BPH14 activation and termination and the role OsNBR1 played in BPH resistance. **a**, Relative transcript levels of *OsATG8s* and *OsNBR1* in *Bph14* and MH63 plants upon the cessation of BPH feeding. **b**, Relative transcript levels of *Bph14*-mediated resistance signaling genes, *OsICS1* and *OsNPR1*, in *Bph14* and MH63 plants upon the cessation of BPH feeding. **c**, Relative transcript levels of *Bph14*-mediated resistance signaling genes, *OsICS1* and *OsNPR1*, in the *Bph14*-*Osnbr1*^*−*^ (*Bph14-*Os*nbr1*) lines (*Bph14*-*Osnbr1*^*−*^-1 and *Bph14*-*Osnbr1*^*−*^-2) and *Bph14* plants upon the cessation of BPH feeding. **d**, BPH weight gain on the *Bph14*-*Osnbr1*^*−*^ lines (*Bph14*-*Osnbr1*^*−*^-1 and *Bph14*-*Osnbr1*^*−*^-2) and *Bph14* plants after 48 h of feeding. **e**, Honeydew excretion by BPHs reared on the *Bph14*-*Osnbr1*^*−*^ lines and *Bph14* plants after 48 h of feeding. **f**, Two-host choice test of BPHs feeding on the *Bph14*-*Osnbr1*^*−*^ lines and *Bph14* plants. **g**, Survival rates of BPHs feeding on the *Bph14*-*Osnbr1*^*−*^ lines and *Bph14* plants for 8 days. In **a**,**b**,**c**, *P* values were derived by one-way ANOVA. Data are mean ± s.d. (*n* = 3, biologically independent experiments). In **d**,**e**, *P* values were derived by one-way ANOVA (*n* = 18, biologically independent samples). In **f**,**g**,*P* values were derived by one-way ANOVA. Data are mean ± s.e.m. (*n* = 10, biologically independent samples). In box plots in **d**,**e**, the box limits indicate the 25th and 75th percentiles, the whiskers indicate the full range of the data, and the center line indicates the median. Individual data points are plotted. Source data

To provide insights into the duration of *Bph14*-mediated immunity, we assessed the longevity of BISP and the immune status of rice plants after BPH feeding had ceased. To this end, BPH-resistant *Bph14* plants and BPH-susceptible MH63 plants were infested with BPHs for 24 h. At this time, insects were removed, and BISP, *Os*ATG8, *Os*NBR1 and WRKY72 levels were monitored for 48 h. In MH63 plants, BISP levels were similar at 0 and 36 h after BPH removal. By contrast, in *Bph14* plants, BISP levels decreased markedly at 6 h and were barely detected at 36 h (Fig. 5a). Correlating well with the decreased BISP levels, the levels of *Os*ATG8 and *Os*NBR1 proteins and their transcripts were more abundant in *Bph14* plants than in MH63 plants (Fig. 5a and Extended Data Fig. 10a). These results suggest that after BPH feeding and secretion of saliva have ceased, BISP is stable for prolonged times in susceptible rice but is rapidly eliminated through autophagy in *Bph14* plants.

Moreover, the degradation of BISP was accompanied by a mitigation of the signal outputs of BPH14-activated resistance (Fig. 5a,b and Extended Data Fig. 10b). For example, WRKY72 protein and transcript levels rapidly reduced (within 12 h) in *Bph14* plants and were slower in susceptible plants after BPH feeding had ceased (Fig. 5a,b). In addition, Os*ICS1* and Os*NPR1* transcripts showed a similar regulation pattern (Extended Data Fig. 10b). The rapid dissipation of BPH14-regulated immunity based on BISP depletion was supported by the dynamics of formation of the BPH14 homomeric complex, which was reduced when BPH14 was expressed with BISP and *Os*NBR1 to induce autophagy (Fig. 5c). These results suggest that autophagy contributes to the elimination of BISP and the attenuation of resistance in *Bph14* plants after BPH feeding is terminated.

Further support for this theory was garnered from examining the fate of BISP and defence-signalling components in *Bph14–*Os*nbr1* plants and WT *Bph14* plants after BPH feeding was terminated. BISP levels were significantly higher in *Bph14–*Os*nbr1* plants than in *Bph14* plants at 0, 24 and 48 h after removing the insects (Fig. 5d), which confirmed that *Os*NBR1 deficiency slowed the degradation of BISP. WRKY72 protein and transcript levels, as well as Os*ics*1 and Os*npr*1 transcripts, were significantly higher at 0–48 h in the *Bph14–*Os*nbr1* plants than the *Bph14* plants (Fig. 5d,e and Extended Data Fig. 10c). This result suggests that the time span of BPH14-activated signalling is extended in *Bph14–*Os*nbr1* plants.

Finally, we examined the impact of *Os*NBR1 on rice resistance to BPHs. Whereas *Bph14* plants were damaged by prolonged BPH infestation (14 days), *Bph14–*Os*nbr1* plants had limited symptoms and displayed a higher level of BPH resistance (Fig. 5f,g). BPH adults preferred *Bph14* plants to *Bph14–*Os*nbr1* plants in a two-host choice test. On the basis of honeydew excretion, weight gain and survival, BPHs had poorer performance when feeding on *Bph14–*Os*nbr1* plants (Extended Data Fig. 10d–g). These results indicate that *Os*NBR1 deficiency enhances BPH resistance in *Bph14* plants.

## Discussion

HPR conferred by NLR proteins is widely used for controlling insect pests^40^. Here we provide molecular and cellular insights into the long-standing question of how plant NLR receptors perceive insect-feeding signals to deploy and modulate ETI resistance mediated by these proteins. Notably, we discovered that the tripartite interactions of BISP, its cognate NLR receptor BPH14 and the selective-autophagy receptor NBR1 enable a self-regulated resistance mechanism to BPHs (Fig. 5h).

In this tripartite interaction system, the BISP effector is secreted into rice leaf sheaths to bind to one of its targets, *Os*RLCK185. By interfering with *Os*RLCK185 autophosphorylation, BISP suppresses rice immune responses to promote BPH feeding and success. However, in resistant rice, the NLR receptor BPH14 directly binds to BISP. Following recognition, resistance is activated, which stops BPHs from ingesting phloem sap^8,17,19^. Constitutive activation of BISP-triggered resistance in *Bph14* plants is detrimental to plant fitness, reducing plant stature and yields. Therefore, mechanisms to temper the resistance response must be deployed to promote plant vitality. The importance of the ubiquitin–proteasome system in effector and NLR protein turnover in pathogen–plant ETI is known^26,41^. Here we revealed that the governor of *Bph14*-mediated resistance is *Os*NBR1-regulated autophagy, which controls BISP turnover. Both BISP and BPH14 physically interact with *Os*NBR1. In turn, *Os*NBR1 interacts with *Os*ATG8 to promote the degradation of BISP and, thereby, fine-tune the level of *Bph14*-mediated resistance. Whether the BISP–BPH14 complex dissociates before BISP turnover by autophagy or whether BPH14 is also degraded by autophagy is an essential topic of research in the future. Finally, although autophagy can be induced by transient reprogramming in response to plant hormones^42^, the role of SA, which is induced in the BISP–BPH14 interaction, remains to be elucidated.

The BISP–BPH14–*Os*NBR1 resistance response adapts to changes in BPH feeding pressure. BPHs terminate feeding when a host becomes undesirable, which can induce individual insects or entire BPH populations to migrate long distances in search of better host plants^15–18^. When BPHs feed on *Bph14* plants, BISP triggers resistance, which makes *Bph14* plants unpalatable and forces the insects to stop feeding. With the cessation of feeding and the departure of insects from these sites, the activated autophagy pathway rapidly degrades BISP to restore cellular homeostasis. Immune systems resume their ‘off’ status, thereby allowing rice to reallocate resources to growth and reproduction. The newly described BISP–BPH14–*Os*NBR1 tripartite interaction system enables rice to resist BPHs without compromising yield performance in the natural habitats^43^. By leveraging the autoregulatory mechanisms used in *Bph14* gene-for-gene resistance in new ways, we have the ability to create a new generation of resistant rice that can feed the increasing world population in a sustainable manner and, significantly, to control insect pests without relying on insecticide sprays.

## Methods

### Plant materials and growth conditions

Nipponbare (designated as *N14*) is a model *O.* *sativa japonica* rice variety that contains the *N14*-susceptible allele of *Bph14*. *N14* was used in early experiments to establish the principles of BISP–BPH14 interactions. The RI35 line (designated as *Bph14*) is a recombinant inbred line that contains the BPH resistance gene *Bph14* (ref. ^8^). Minghui 63 (MH63) is the BPH-susceptible parent of RI35 (ref. ^44^) and is a model variety for *O.* *sativa* *indica* rice breeding and genomics. MH63 and BPH14 were used for most studies to elucidate BISP–BPH14–NBR1 interactions owing to the agronomic importance of MH63. The following transgenic lines were developed in this study: *N14–Bisp* (*pUBI::Bisp-Myc*, *N14* recipient), *Bph14–Bisp* (*pUBI::Bisp–Myc*, *Bph14* recipient), Os*rlck185* (*Os*RLCK185–Cas9, ZH11 recipient), *Bph14–*Os*nbr1* (*Os*NBR1–Cas9, *Bph14* recipient). The Os*rlck185* mutants were generated in the ZH11 background^45^; ZH11 is a readily transformable *O.* *sativa* *japonica* rice that BPH-susceptible genotype.

### Insect materials and growth conditions

The BPH insects were maintained on susceptible cultivar Taichung Native 1 at 28 ± 2 °C with 60–80% relative humidity and a photoperiod of 16 h of light–8 h of dark. RNAi of BPH by dsRNA injection was developed as part of this study: *GFP*-RNAi (microinjected with ds*GFP*) and *Bisp*-RNAi (microinjected with ds*Bisp*).

### BPH bioassays on rice plants

To evaluate BPH resistance of transgenic and WT rice plants, sets of around 15 or 20 seeds from the same plant were sown in a plastic cup (10 cm in diameter, 20 cm in height). At the three-leaf stage, all plants were placed under a large gauze cover, and each seedling was infested with ten second- or third-instar BPH nymphs. When all of the susceptible plants had died (scored as 9), each seedling of the other cultivars or lines was given a score of 0, 1, 3, 5, 7, or 9 according to the degree of damage, as previously described^8,46,47^, and the BPH resistance score was calculated. At least three replicates were used for each cultivar or line.

To measure the weight gain and honeydew excretion of BPHs, a newly emerged short-winged female adult and a Parafilm sachet were weighed using a 1/100,000 Shimadzu AUW120D electronic balance^11,12^. The insect was placed in the pre-weighed sachet, which was then attached to the leaf sheath near the lower part of a rice plant. After 48 h, the insect and Parafilm sachet were weighed again. The difference between the first and second measurements of the weight of the insect was recorded as the BPH weight gain; the difference between the first and second measurements of the weight of the sachet was recorded as amount of honeydew excreted. At least ten replicates were used for each cultivar or line.

For the two-host choice tests, a transgenic plant and a WT plant were grown in a plastic cup as described elsewhere^10–12^. At the four-leaf stage, the cup was covered with gauze, and 20 or 30 second- or third-instar nymphs were released into the cup (the exact number depended on the BPH resistance of the WT plant). The number of nymphs that settled on each plant was recorded at 6, 12, 24, 48, 72, 96, and 120 h after release (the moment of release was considered time 0). Ten cups were analysed for each cultivar or line examined.

To study BPH nymph survival on rice, second- or third-stage BPH nymphs were released (ten insects per plant), and the cups were covered with a light-transmitting mesh. The nymphs on each plant were counted 8 or 9 days after release. Survival rates were calculated as the number of surviving nymphs divided by the total number of nymphs released at the start of the experiment. Ten cups were analysed for each cultivar or line.

### Plasmid construction

Routine molecular cloning techniques were used to prepare the constructs. The plasmids and primers used in this work are listed in Supplementary Table 3. All of the resulting recombinant vectors were sequenced.

To construct the plasmid for Y2H screening, the full-length *Bph14* sequence was amplified from *Bph14* leaf sheath cDNA and cloned into the *Xma* I site of the pGBKT7 vector, which produced the BPH14–BD vector.

To construct the plasmids for the Y2H assays, the coding sequences of *Bisp* (ORF sequence without its 1–25 amino acid residue signal peptide; amino acids 26–241), *Bisp*^26–124^(amino acids 26–124) and *Bisp*^125–241^ (amino acids 125–241) were amplified and cloned into the *Xma* I site of the pGADT7 vector to produce BISP–AD, BISP(26–124)–AD and BISP(125–241)–AD, respectively. The coding sequences of the CC, NB and LRR domains and full-length *Bph14* were amplified from the BPH14-BD vector and cloned into the *Xma* I sites of the pGADT7 and pGBKT7 vector to produce CC–AD, NB–AD, LRR–AD, BPH14–AD, CC–BD, NB–BD, and LRR–BD, respectively. The coding sequence of *N14* (*Bph14* allele in the rice variety Nipponbare) was amplified from *N14* leaf sheath cDNA and cloned into the *Xma* I site of the pGBKT7 vector to produce N14–BD.

Eight kinases that were differentially phosphorylated during BPH-infested and non-infested plants were chosen for this study. The coding sequences for the Os*RLCK185*, Os*RLCK185*^1–85^ (amino acids 1–85), Os*RLCK185*^KD^ (kinase domain, amino acids 86–353), Os*RLCK185*^354–491^ (amino acids 354–491), Os*MAPKKK*ε, Os*MKK6*, Os*MPK20*, Os*MPK16*, Os*CDPK14*, Os*CDPK20*, and Os*RLCK259* fragments were amplified from *N14* leaf sheath cDNA and cloned into the pGBKT7 vector to produce *Os*RLCK185–BD, *Os*RLCK185(1–85)–BD, *Os*RLCK185^KD^–BD, *Os*RLCK185(354–491)–BD, *Os*MAPKKKε–BD, *Os*MKK6–BD, *Os*MPK20–BD, *Os*MPK16–BD, *Os*CDPK14–BD, *Os*CDPK20–BD, and *Os*RLCK259–BD, respectively. The coding sequences of Os*NBR1*, Os*ATG8a*, Os*ATG8b*, Os*ATG8c*, and Os*ATG8e* were amplified from *Bph14* leaf sheath cDNA and cloned into pGBKT7 and pGADT7 to produce *Os*NBR1–BD, *Os*ATG8A–BD, *Os*ATG8B–BD, *Os*ATG8C–BD, and *Os*ATG8E–BD, respectively.

Constructs used for rice protoplast transfection were generated using pCXUN-4×HA and pCXUN-4×Myc^19^. The coding sequences of the CC, NB and LRR domains, *Bph14*, *N14*, the N14 LRR domain (N14–LRR), *Wrky72, Bisp* (ORF sequence without its signal peptide), *Bisp*^26–124^, *Bisp*^125–241^, Os*RLCK185*, Os*RLCK259*, Os*NBR1*, Os*NBR1* mutant N1, Os*ATG8a*, Os*ATG8b*, Os*ATG8c*, and Os*ATG8e* were amplified and cloned into the pCXUN-4×Myc vector to produce CC–MYC, NB–MYC, LRR–MYC, BPH14–MYC, N14–MYC, N14–LRR–MYC, WRKY72–MYC, BISP–MYC, BISP(26–124)–MYC, BISP(125–241)–MYC, *Os*RLCK185–MYC, *Os*RLCK259–MYC, N1–MYC, *Os*NBR1–MYC, MYC–*Os*ATG8A, MYC–*Os*ATG8B, MYC–*Os*ATG8C, and MYC–*Os*ATG8E, respectively. Meanwhile, the fragments for the GFP, CC, NB, LRR domains, *Bph14*, *N14*, the N14 LRR domain (N14–LRR), *Bisp*, *Bisp*^26–124^, *Bisp*^125–241^, Os*RLCK185*, and Os*NBR1* were amplified and cloned into the pCXUN-4×HA vector to produce GFP–HA, CC–HA, NB–HA, LRR–HA, BPH14–HA, N14–HA, N14–LRR–HA, BISP–HA, BISP(26–124)–HA, BISP(125–241)–HA, *Os*RLCK185–HA, and *Os*NBR1–HA, respectively. Additionally, we generated a series of Os*nbr1* fragments, including the isolated N1 (amino acids 1–755), N2 (amino acids 756–832), N3 (amino acids 1–782), N4 (amino acids 1–755 and 798–832), and mutant N5 (K13A), N6 (WL788/791AA) and N7 (K13A and WL788/791AA) fragments and cloned them into the pCXUN-4×HA vector to produce N1–HA, N2–HA, N3–HA, N4–HA, N5–HA, N6–HA, and N7–HA, respectively.

To analyse the subcellular localization of BISP, the coding sequence of *Bisp* (ORF sequence without its signal peptide) was cloned downstream of the maize (*Zea mays*) *Ubiquitin 1* promoter, in-frame with *GFP* in the binary vector pCAMBIA1300, which produced the BISP–GFP construct.

For BiFC analysis, the coding sequences of *Bph14*, *N14*, *Bisp* (ORF sequence without its signal peptide), and *Bisp*^125–241^ were cloned in-frame into the *Xma* I sites of the pUSYNE and pUSYCE vectors^19^, which produced the constructs BISP–YN, BISP(125–241)–YN, BPH14–YC, and N14–YC, respectively.

The *Escherichia coli* recombinant protein vectors used for the expression and purification of proteins in the phosphorylation activity assays, designated pET-MBP-His and pET-GST-His, were created by adding a C-terminal MBP or GST tag, respectively, to the pET-28a expression vector (EMD Biosciences, Novagen). The coding sequences of *Bisp* (ORF sequence without its signal peptide), *Bisp*^26–124^ and Os*RLCK185* were amplified and cloned into the pET-MBP-His and pET-GST-His vector, which produced *Os*RLCK185–MBP–His, BISP(26–124)–GST–His and BISP–GST–His constructs, respectively.

For the *N.* *benthamiana* leaf agroinfiltration experiments, the coding sequences of *Bph14*, *N14*, Os*NBR1,* and N1 (an *Os*NBR1 mutant) were cloned into the pEarleyGate 203 vector^48^ to produce BPH14-203, N14-203, *Os*NBR1-203, and N1-203, respectively. The coding sequence of *Bisp* (ORF sequence without its signal peptide) was cloned into the pEarleyGate 201 and pEarleyGate 101 vector^48^ to produce BISP-201 and BISP-101 (BISP–YFP), respectively.

To generate the CRISPR–Cas9 construct, the target sites for CRISPR–Cas9 were designed using the web-based tool CRISPR-P (v.2.0; http://crispr.hzau.edu.cn/CRISPR2/). The CRISPR–Cas9 binary construct was prepared as previously described^49^, which produced the construct *Os*NBR1–Cas9.

### Y2H screening

The Matchmaker Gold Yeast Two-Hybrid System (Clontech, 630489) was used to screen for BPH14-interacting proteins. Y2H Gold cells carrying BPH14–BD were mixed with 1 ml of a BPH salivary gland cDNA library and incubated overnight before plating on TDO (SD/-Leu-Trp-His)-selective medium. Candidate clones growing on TDO medium were confirmed following the manufacturer’s protocol (Clontech, 630489). The BPH cDNA library was constructed using the Make Your Own “Mate & Plate” Library System (Clontech, 630490) with a simple and highly efficient protocol. Total RNA was isolated from BPH salivary glands using TRIzol reagent (Takara, 9109), and cDNA was synthesized from the isolated mRNA using SMART cDNA synthesis technology (Clontech, 634926).

For the Y2H assay of protein interactions, the yeast strain AH109 was transformed with the indicated bait and prey plasmids according to the manufacturer’s instructions. Co-transformants were simultaneously plated on selection medium containing DDO (SD/-Leu-Trp), TDO (SD/-Leu-Trp-His) with the appropriate concentration of 3-amino-1,2,4-triazole (3-AT) or QDO (SD/-Leu-Trp-His-Ade) and incubated at 30 °C.

### Cloning and sequencing of *Bisp* cDNA

The cDNA sequences of *Bisp* were obtained from BPH salivary gland transcriptomes^5^. To obtain the full-length counterparts of the truncated sequences in the transcriptomes, 5′- and 3′-RACE amplification was performed using a SMARTer RACE cDNA Amplification kit (Clontech, 634923) following the manufacturer’s instructions. *Bisp*-specific primers were obtained based on sequencing data. The RACE products were amplified, and the purified products were ligated into the pMD18-T vector (Takara, 6011) and sequenced.

### Protein extraction, immunoblot analysis and in vivo co-IP assays

Proteins were extracted from the leaf sheaths of rice seedlings at the four-leaf stage. The leaf sheaths were wiped with a cotton ball soaked in alcohol to remove honeydew and ground in liquid nitrogen. Total protein was extracted from the leaf tissue in rice protein extraction buffer (50 mM Tris-HCl pH 7.5, 150 mM NaCl, 10% glycerol, 0.1% NP-40, plant protease inhibitor cocktail (Roche)). Equal amounts of total protein were analysed by SDS–PAGE and detected by immunoblotting using anti-actin (Abbkine, ABL1050; 1:3,000), anti-BISP (1:500), anti-*Os*WRKY72 (Beijing Protein Innovation, AbP80456-A-SE; 1:500) and anti-*At*NBR1 (Agrisera, AS194281; 1:1,000) antibodies. For *Os*ATG8 analysis, equal amounts of proteins were subjected to SDS–PAGE with 6 M urea^50^ and detected by immunoblotting using anti-*At*ATG8A antibodies (Abcam, ab77003; 1:1,000). The anti-BISP and anti-NISP1 antibodies were prepared by expressing BISP and NISP1 (cloned into pET28a) in *E.* *coli* strain BL21 (DE3), respectively. The expressed recombinant proteins were collected and injected into two rabbits (DIA·AA Biotech). Ponceau S solution (Sigma-Aldrich, P7170) was used to stain the PVDF membrane and was used as a loading control. The intensities of protein signals were quantified using ImageJ.

Protein samples from rice protoplasts were prepared in rice protein extraction buffer (100 mM Tris-HCl pH 7.5, 1 mM EDTA, 5 mM MgCl_2_, 0.5% (w/v) Triton X-100, with a plant protease inhibitor cocktail (Roche)). Total soluble proteins were extracted from rice protoplast samples, each in 100 µl of rice protoplast protein extraction buffer. Next, 10 µl of the extract was separated by SDS–PAGE and subjected to immunoblotting using anti-HA (MBL, M180-3; 1:1,000), anti-MYC (MBL, M192-3; 1:1,000) or anti-actin (Abbkine, ABL1050; 1:3,000) antibodies.

For the co-IP assays, rice protoplasts were incubated at 28 °C for 14–20 h after transfection. Total protein extracts were collected from the protoplasts in the rice protein extraction buffer as described above. The supernatants were incubated with 10 μl Protein G Agarose beads (Millipore, 16–266) and 1 μl anti-HA (MBL, M180-3) or 1 μl anti-MYC (MBL, M192-3) antibodies at 4 °C for 5 h, and detected using anti-HA or anti-HA mAb-HRP-DirecT (MBL, M180-7, clone: TANA2; 1:1,000), anti-actin and anti-MYC or anti-MYC mAb-HRP-DirecT (MBL, M192-7, clone: My3, 1:1,000) antibodies, respectively. The intensities of protein signals were quantified using ImageJ.

For the yeast protein immunoblots, total yeast protein was extracted as previously described^51^. In brief, single yeast colonies were resuspended in 4 ml YPDA liquid medium and grown to OD_600_ = 0.6 at 30 °C. The pelleted yeast cells were resuspended in 1 ml distilled water, combined with 100 μl 0.2 M NaOH, incubated for 5 min at room temperature and pelleted and resuspended in 50 μl loading buffer. The proteins were boiled for 10 min at 95 °C and detected by immunoblotting with anti-HA or anti-MYC antibodies. Ponceau S solution was used to stain the PVDF membrane for loading control.

### Rice protoplast isolation and transfection

Rice protoplasts were prepared from etiolated seedlings and transfected as previously described^11,19^. About 3–10 μg of plasmid DNA was used to transfect around 2 × 10^5^ protoplasts using the PEG-mediated method. The fluorescent or epitope-tagged proteins were detected at 14–20 h after transfection.

### Subcellular localization and BiFC analysis

For subcellular localization, the BISP–GFP plasmid and the nuclear marker bZIP63–RFP^11,19^ were transformed into rice protoplasts. Following incubation at 28 °C for 14–20 h, the protoplasts were imaged using a confocal microscope (Leica, DMi8).

For BiFC analysis, protoplasts were transfected with the indicated expression vectors. Following incubation at 28 °C for 14–20 h, the protoplasts were imaged by confocal microscopy. BiFC fluorescent signals were quantified using previously described methods^52^. The intensity of YFP and CFP signals in individual protoplasts was determined using ImageJ software. The average YFP/CFP intensity ratio (per cent) of the whole cell was measured, and the means were calculated from five to ten independent cells. The fluorescent or epitope-tagged proteins were detected with anti-GFP (Roche, 11814460001; 1:1,000), anti-HA or anti-MYC antibodies.

### RNA isolation, real-time and semi-quantitative RT–PCR analysis

Total RNA was isolated from rice leaf sheaths, BPHs or *N.* *benthamiana* leaves using TRIzol reagent (Takara, 9109). First-strand cDNA was synthesized using a PrimeScript RT Reagent kit with gDNA Eraser (Takara, RR047A) following the manufacturer’s instructions.

Real-time RT–qPCR was carried out using a CFX96 Real-Time system (Bio-Rad) with SYBR Green Supermix (Bio-Rad, 172–5274) following the manufacturer’s instructions. For RT–qPCR in rice, three housekeeping genes, Os*ubiquitin*, Os*tbp* and Os*hsp*^53^, were used as reference genes for calibration of real-time RT–PCR data. For RT–qPCR in BPH, Nl*RPS18*, Nl*ubiquitin* and Nl*ACTB* (which encodes β-actin) were used as reference genes. The sequences of the primers used are listed in Supplementary Table 3.

For the semi-quantitative RT–PCR assays, first-strand cDNA (5 μl) was diluted to 50 μl final volume with TE buffer, and 1 μl of the dilution was used as template for PCR amplification using gene-specific primers. The internal standard Nb*ACTB* was used. The number of cycles used for amplification with each primer pair was adjusted to ensure that the amplification was in the linear range. The PCR products were sequenced to ensure that they were derived from the targeted genes. The sequences of the primers used are listed in Supplementary Table 3.

### Immunohistochemistry

For immunolocalization of BISP in rice leaf-sheath sections, *N14* plants at the two-leaf stage were infested with ten fourth- or fifth-instar nymphs for 2 days or served as non-infested controls. The outer leaf sheaths were collected, fixed, dehydrated, and embedded in paraffin (Paraplast Plus, Sigma-Aldrich, P3683) as previously described^11,17^. The sections were incubated in dimethylbenzene and an alcohol gradient to remove the paraffin and to gradually rehydrate. The sections were incubated with anti-BISP antibodies or pre-immune rabbit serum (prepared as described above, 1:100) at 4 °C in the dark overnight. The next day, the sections were washed 3 times (15 min each time) with PBST (PBS with 0.1% (v/v) Triton X-100), blocked with 5% bovine serum albumin in PBST at room temperature for 1 h, washed 3 times at 15-min intervals with PBST, and incubated with the secondary Cy3-conjugated goat anti-rabbit antibodies (Jackson ImmunoResearch, 111-165-003, 1:500) at 4 °C overnight. The sections were washed extensively with PBST at 15-min intervals and imaged under a confocal microscope (Leica, DMi8).

For immunolocalization in BPH salivary glands and guts, female or male insects were anaesthetized with carbon dioxide (CO_2_). The salivary glands and guts were dissected in 0.65% NaCl, washed 3 times in PBST and fixed in 4% (v/v) formaldehyde in PBST at 4 °C overnight. The next day, the organs were extensively washed with PBST, incubated with anti-BISP antibodies, pre-immune serum or anti-NlSP1 antibodies^54^ (1:100) at 4 °C in the dark overnight, and treated as described above. Nuclei were stained with 100 nM 4,6-diamidino-2-phenylindole (DAPI) at room temperature in the dark for 5 min. The salivary glands and guts were extensively washed with PBST and placed on a glass slide. Photographs were taken under a confocal microscope (Leica, DMi8). For the quantification of Cy3, the Cy3 and DAPI fluorescence signals in individual cells were quantified using ImageJ. The average Cy3/DAPI intensity ratio of each cell was measured, and mean ratios were calculated from five to ten independent cells.

### RNAi and bioassay of BPHs after dsRNA injection

A 506-bp fragment of *Bisp* was amplified with the primers listed in Supplementary Table 3, including the T7 promoter sequence in the forward primer. After cloning the *Bisp* fragment into the pMD18-T vector (Takara, 6011), dsRNA was synthesized from PCR-generated DNA templates using a MEGAscript T7 Transcription kit (Ambion, AM1354). BPH nymphs in their third, fourth or fifth stage were injected with *Bisp-*RNAi or *GFP-*RNAi using a Nanoliter 2010 injector (World Precision Instruments) as previously described^5^. The efficiency of gene silencing was determined by RT–qPCR at 1–10 days after dsRNA treatment.

After the injected BPHs were placed and reared on rice plants for 12 h, the survival rate, weight gain and honeydew excretion of *GFP*-RNAi BPHs, *Bisp*-RNAi BPHs and non-injected control insects were measured as described above. Five replicates were set up for each treatment to measure BPH survival rates, and 20 replicates were performed for each treatment group to measure weight gain and honeydew excretion.

### Plant transformation

To constitutively express *Bisp* in rice plants, the BISP–MYC plasmids were transformed into *N14* or *Bph14* plants through *Agrobacterium*-mediated transformation^8^. To knockout *Os*NBR1 by genome editing, the CRISPR–Cas9 plasmids *Os*NBR1*–*Cas9 were introduced into *Agrobacterium tumefaciens* strain EHA105 and transformed into *Bph14* as described elsewhere^8^. Genomic DNA was extracted from these transformants, and PCR amplification was performed using primer pairs flanking the designed target site. The PCR products were sequenced to determine whether the gene editing was successful.

### Quantification of free SA

Endogenous free SA levels were determined in rice leaf sheaths. Fifteen leaf sheaths from each plant or line were ground to a fine powder in liquid nitrogen as a replicate. Three biological replicates of each frozen sample were prepared. Each sample (100 mg) was added to 750 μl precooled extraction buffer (methanol:water:acetic acid, 80:19:1, v/v/v) supplemented with 3 µg naphthaleneacetic acid (Sigma-Aldrich, 35745) as an internal standard and shaken at 4 °C overnight. After centrifugation, the supernatant was filtered through a 0.22-μm nylon membrane and dried under a nitrogen flow at room temperature for at least 4 h. Finally, 200 μl methanol was added to dissolve the extracts. Endogenous free SA measurements were performed as described elsewhere^55^.

### Recombinant protein expression and purification in *E.coli* and BISP antibody specificity analysis

The *Os*RLCK185–MBP–His, BISP–GST–His and BISP(26–124)–GST–His constructs were expressed in *E.* *coli* BL21 (DE3) cells. GST-tagged or MBP-tagged recombinant proteins were purified using Glutathione Sepharose beads (GE Healthcare, 17075601) and Amylose Resin (New England Biolabs, E8022S), respectively, according to the manufacturer’s instructions. Protein concentrations were determined by BCA protein assay (Beyotime, P0010S).

For the BISP antibody specificity analysis, the purified BISP–GST–His protein and total protein from whole insect and a twofold dilution series were separated on SDS–PAGE gels. BISP–GST–His was detected in immunoblots using anti-BISP and anti-His (GenScript, A00186; 1:2,000) antibodies, respectively. Ponceau S solution was used to stain the PVDF membrane and used as the total BPH protein loading control.

### In vitro phosphorylation assays

To test the autophosphorylation of *Os*RLCK185, 1 µg purified recombinant *Os*RLCK185–MBP–His, BISP(26–124)–GST–His or BISP–GST–His was incubated in 25 µl kinase reaction buffer containing 50 mM HEPES (pH 7.5), 10 mM MgCl_2_, 1 mM DTT, 1 mM ATP, and 2.5 µCi [γ-^32^P]ATP at 30 °C for 10–30 min. The kinase reaction was subsequently analysed using a 10% SDS–PAGE gel for autoradiography. A similar gel was subjected to Coomassie Brilliant Blue staining as a control.

To test the phosphorylation of *Os*RLCK185 at the serine and threonine residues, phosphorylated *Os*RLCK185 was detected by immunoblotting with anti-phosphoserine/phosphothreonine antibodies (Millipore, 05-368, 1:1000) and anti-His antibody (GenScript, A00186).

### Recombinant protein expression and purification in insect cells and BLI analyses

LRR, N14–LRR, BISP, BISP(26–124), and BISP(125–241) were cloned into pFastBac 1 with an N-terminal 6×His-SUMO tag and expressed in SF9 insect cells (Thermo Fisher Scientific, 11496015) at 27 °C. SF9 cells (500 ml) cultured in Sf-900 II SFM medium (Invitrogen, 10902088) were infected with 25 ml recombinant baculovirus and were cultured for 4 days at 27 °C. Cells were collected and re-suspended in PBS solution (2 mM KH_2_PO_4_, 8 mM Na_2_HPO_4_, 136 mM NaCl, and 2.6 mM KCl, pH 7.4). After sonication and centrifugation, recombinant LRR, N14–LRR, BISP, BISP(26–124), and BISP(125–241) proteins in the supernatant were purified with Ni-NTA beads (Novagen,70666-4) and then were dialysed in PBS solution.

The binding kinetics and affinities of LRR and N14–LRR with BISP, BISP(26–124) or BISP(125–241) was determined using an Octet RED96 system (FortéBio) as previously described^56,57^. Purified LRR and N14–LRR were biotinylated with NHS-biotin (Sangon Biotech, C100212) according to the manufacturer’s instructions. All streptavidin-coated biosensors (FortéBio) were hydrated in PBS buffer (2 mM KH_2_PO_4_, 8 mM Na_2_HPO_4_, 136 mM NaCl, 2.6 mM KCl, 0.05% Tween 20, and 0.5% bovine serum albumin, pH 7.4) for 10 min. Biotinylated LRR and N14–LRR were diluted in PBS buffer to a final concentration of 20 μg ml^−1^ and immobilized onto a streptavidin-coated biosensor. Biosensors with immobilized LRR and N14–LRR were diluted in binding buffer containing different concentrations of BISP, BISP(26–124) or BISP(125–241) for the binding kinetics analysis (association step). Subsequently, the streptavidin-coated biosensor was returned to PBS buffer for 180 s (dissociate step). PBS buffer was used in all BLI experiments to reduce nonspecific binding to the biosensors except for experiments in the presence of BISP, BISP(26–124) or BISP(125–241). The experiments included the following steps at 25 °C: (1) loading (60 s); (2) baseline (120 s); (3) immobilization of proteins onto sensors (60 s); (4) association (180 s); and (5) disassociation (180 s). The data were analysed, and sensor-grams were step-corrected and reference-corrected. Global fitting of the kinetic rates was performed, then the equilibrium dissociation constant (*K*_d_), association (*K*_on_) and dissociation (*K*_off_) rate constants and their standard errors were analysed using FortéBio data analysis software (v.1.1.0.16, FortéBio). The assays were repeated two times for each affinity measurement.

For the competition assay, an Octet RED96 system (FortéBio) was used as previously described^58^. Biotinylated LRR was diluted in PBS buffer to a final concentration of 20 μg ml^−1^ and immobilized onto a streptavidin-coated biosensor. The association of BISP(26–124) or PBS was measured for 360 s at 25 °C. Next, the competitors (BISP, PBS or BISP(26–124)) were added to the BISP(26–124)-associated or PBS-associated samples. The experiments included the following steps at 25 °C: (1) loading (60 s); (2) baseline (120 s); (3) immobilization of protein onto sensors (60 s); (4) baseline (240 s); (5) association with BISP(26–124) or PBS (360 s); (6) disassociation (240 s); (7) association with the competitors BISP, PBS or BISP(26–124) (120 s); and (8) dissociation (180 s). The data were analysed using FortéBio data analysis software (v.1.1.0.16, FortéBio). The assays were repeated two times for each measurement.

### MST assay

The MST assay was performed as previously described^59,60^. The affinity of purified LRR or N14–LRR with BISP, BISP(26–124) or BISP(125–241) was measured using a Monolith NT.115 (Nanotemper Technologies). Proteins were fluorescently labelled using a Monolith Protein Labelling kit (RED-NHS 2nd Generation; Nanotemper Technologies, MO-L011) according to the manufacturer’s protocol, and the labelled protein used for each assay was about 20 nM. A solution of unlabelled BISP, BISP(26–124) or BISP(125–241) protein was diluted for an appropriate serial concentration gradient. The samples were loaded into capillaries (Nanotemper Technologies, MO-K022) after incubation at room temperature for 5 min. Measurements were performed in 20 μl of PBS buffer containing 0.05% Tween 20. The assays were repeated three times for each affinity measurement. Data analyses were performed using Nanotemper Analysis software provided by the manufacturer.

For the competition assays between LRR with BISP and BISP(26–124) by MST, 50 nM LRR proteins and 200 nM BISP(26–124) were combined in PBS buffer with 0.05% Tween 20 and incubated for 10 min to enable protein interactions. A series of different concentrations of BISP was then added to the LRR–BISP(26–124) mixture before measurement. The assays were repeated three times for each measurement. The dissociation constant (*K*_i_) from the MST assays was calculated using the *K*_i_ Finder software from the Nanotemper website (https://nanotemper.my.site.com/explore/s/article/Can-competition-assays-be-performed-with-MST).

### Inhibition of protein degradation in rice protoplasts

After PEG-mediated transformation, the rice protoplasts were placed in 1 ml W5 buffer containing 10 μM 3-MA (3-methyladenine, Selleck, S2767), 100 μM chloroquine (MCE, HY-17589A), 100 μM leupeptin (MCE, HY-18234A), or 20 μM E-64d (Aloxistatin, Sigma-Aldrich, E8640) to inhibit autophagy or 50 μM MG132 (Selleck, S2619) to inhibit the 26S proteasome. The treated protoplasts were incubated at 28 °C for 14–20 h before protein extraction. Anti-actin (Abbkine, ABL1050) was used as a reference to quantify total protein levels. Rice WRKY72 is degraded by the 26S proteasome pathway^19^ and was used as a positive control to ensure that the proteasome inhibitor MG132 was active. The rice HD1 protein is degraded in the dark by the autophagy pathway^61^, and *Os*NBR1 was used as a positive control to assure autophagy inhibitors were active. The intensity of protein signals in immunoblots was quantified by ImageJ.

### Live-cell imaging of autophagic structures and virus-induced gene silencing in *N.benthamiana* leaves

To observe autophagic structures in *N.* *benthamiana* leaves by confocal microscopy, *A.* *tumefaciens* strain GV3101 cells harbouring CFP–*Nb*ATG8F (designed as CFP–ATG8F)^27^ and the constructed expression vectors, BISP-201, BPH14-203, N14-203, *Os*NBR1-203, and N1-203 were infiltrated into *N.* *benthamiana* leaves for 60 h. The samples were then infiltrated with or without the autophagy inhibitor ConA (first made in DMSO at 0.5 M, then 1:1,000 added) for 10 h before being examined under a confocal microscope (Leica, DMi8).

For virus-induced gene silencing (VIGS) assays, pTRV1 and pTRV2 or its derivatives were introduced into *Agrobacterium* strain GV3101. VIGS assays were performed as previously described^62^. In brief, the *Agrobacterium* cultures with pTRV1 and pTRV2 or its derivatives were mixed at a 1:1 ratio and were infiltrated into the leaves of six-leaf stage *N.* *benthamiana* plants. Silenced phenotypes appeared in the upper leaves about 10 days after infiltration. *A.* *tumefaciens* strain GV3101 cells harbouring CFP–*Nb*ATG8F and the constructed expression vectors BISP-101 (BISP–YFP) and BPH14-203 were infiltrated into the *N.* *benthamiana* upper leaves.

### Transmission electron microscopy

For immunogold electron microscopy, *N14* leaf sheaths infested with BPH for 24 h were cut into 0.1-mm pieces, fixed in precooled 3% paraformaldehyde with 0.1% glutaraldehyde (in 100 mM PBS, pH 7.4) and immediately placed under a vacuum until the samples were completely submerged in the liquid. The samples were transferred to fresh 0.1% glutaraldehyde and incubated at 4 °C overnight. The next day, the samples were washed 3 times with PBS (pH 7.4) for 30 min. The samples were cleared by successive washes with 30% and 50% ethanol for 60 min each at 4 °C, then 70%, 80%, 85%, 90%, and 95% ethanol for 30 min each at −20 °C, and finally 100% ethanol 3 times for 60 min each at −20 °C. After ethanol washes, the samples were embedded in Lowicry K4M resin stepwise, according to the manufacturer’s instructions. Ultrathin sections (70 nm) of the embedded tissues were cut with a diamond knife using an Ultracut E Ultramicrotome (Reichart-Jung) and collected on 3-mm copper (mesh) grids. Immunolabelling of leaf sections was done using standard procedures as previously described^59^ with anti-BISP antibodies or pre-immune serum at 100 µg ml^–1^ and anti-rabbit IgG gold-coupled secondary antibodies (Sigma-Aldrich, G7277- 4ML) at 1:50 dilution. The sections were then stained with 2% uranyl acetate (in 70% ethanol) for 15 min, washed in distilled water 3 times, stained with lead citrate for 12 min, washed in NaOH buffer, washed in distilled water 3 times, and examined under a JEM-1230 electron microscope.

For transmission electron microscopy observations, 2-week-old *Bph14*–*Bisp* transgenic lines, *Bph14* and MH63 leaf sheaths were cut into 0.1-mm pieces, fixed in precooled 4% paraformaldehyde with 2% glutaraldehyde (in 100 mM PBS, pH 7.4) and immediately placed under a vacuum until the samples were completely submerged in the liquid. The samples were transferred to fresh 2% glutaraldehyde and incubated at 4 °C overnight. The next day, the samples were washed 5 times with PBS (pH 7.4) for 20 min and submerged in a 1% solution of osmium tetroxide at room temperature for 1–2 h until they turned completely black because of oxidation. The samples were washed 5 times with PBS at 20-min intervals. The samples were cleared by successive washes with 15%, 30%, 50%, and 70% ethanol for 30 min each, then 80%, 85%, 90%, and 95% ethanol for 20 min each, and finally 100% ethanol twice for 45 min each. After ethanol washing, the samples were embedded in Spurr resin stepwise as instructed by the manufacturer. Ultrathin sections (70 nm) of the embedded tissues were cut with a diamond knife using an Ultracut E Ultramicrotome (Reichart-Jung), collected on 3-mm copper (mesh) grids, stained with 2% uranyl acetate (in 70% ethanol) for 10 min, washed in distilled water 3 times, stained with lead citrate for 15 min, washed in NaOH buffer, and examined under a JEM-1230 electron microscope. To quantify autophagosomes, the double-membrane autophagosomes in every eight transmission electron microscope images were counted and served as one biological replicate; each genotype had five replicates.

### BPH infestation

Seeds were sown in 10-cm diameter plastic cups and grown in a plant growth chamber (CONVIRON PGC2000) as described elsewhere^11^. To analyse the relationship between BISP levels and autophagy, 4-leaf rice plants were infested with second- or third-instar BPH nymphs (10 per plant) at the selected time points (6, 12, 24, 48, and 72 h before the end of the experiment). Control groups were maintained in parallel but without BPH infestation. At the end of the treatments, leaf sheaths were wiped with cotton wool balls soaked in alcohol to remove honeydew, leaf sheaths were excised, frozen, and ground to a fine powder in liquid nitrogen, and proteins isolated. Immunoblotting was performed following the procedures described above in the section ‘Protein extraction, immunoblotting and in vivo co-IP assays’. All treatments were repeated three times.

To measure autophagy and BISP levels following BPH infestation, 4-leaf rice plants were infested with second- or third-instar BPH nymphs (10 per plant) for 24 h. The insects were then removed, and the plants were allowed to grow under standard conditions (described above). At the selected time points after BPH removal, honeydew was removed from leaf sheaths, leaves were excised, frozen, and ground to a fine powder in liquid nitrogen, and proteins isolated. Immunoblotting was performed following the procedures described above. All treatments were repeated three times.

For the autophagy inhibitor ConA treatment, the BPH-infested and non-infested rice plants were treated with or without ConA (10 μM) for 12 h before samples were collected. Leaf sheath total protein extraction and immunoblotting were performed according to the procedures described above. All treatments were repeated three times.

### Agronomic performance test

To investigate the agronomic performance of *Bph14* and *Bph14*–*Bisp* plants, plants were grown in a field in Wuhan, China, under routine management. At harvest, the middle plants in the central row of each plot were assessed for the following nine agronomic traits: plant height, sword leaf length, sword leaf width, number of panicles per plant, number of filled grains per panicle, number of filled grains per plant, percentage of filled grain, 1,000-grain weight, and grain yield per plant^63^.

### Statistical analysis

No statistical methods were used to predetermine the sample sizes. The experiments were not randomized, and investigators were not blinded to allocation during the experiments and outcome assessment. Quantification analyses of protein abundance were conducted using ImageJ software. All values are presented as the mean ± s.d. or mean ± s.e.m. as indicated. Data points are plotted on the graphs, and the number of samples is indicated in the corresponding figure legends. In all cases, sampling for replicates and between time points was performed by collecting material from new plants. All statistical analyses were performed by one-way analysis of variance with MS Excel and GraphPad Prism (v.8.0.1, GraphPad Software). Detailed information about statistical analysis values for all experiments is provided in Supplementary Table 4.

### Reporting summary

Further information on research design is available in the Nature Portfolio Reporting Summary linked to this article.

## Online content

Any methods, additional references, Nature Portfolio reporting summaries, source data, extended data, supplementary information, acknowledgements, peer review information; details of author contributions and competing interests; and statements of data and code availability are available at 10.1038/s41586-023-06197-z.

## Supplementary information

Supplementary Fig. 1 Uncropped blots and gel images.

Reporting Summary

Supplementary Fig. 2 The other two repeats of Fig. 1a–e,h–k.

Supplementary Table 1 New rice varieties introgressed with *Bph14* and authorized for release into production.

Supplementary Table 2 BPH14-interacting proteins from BPH identified in a Y2H assay.

Supplementary Table 3 List of primers used in this study.

Supplementary Table 4 Statistical summary—a summary of all statistical analysis is provided. Source data for all of the statistical analysis can be found in the source data files.

## Data Availability

All data are available within this article and its Supplementary Information. Original gel blots are shown in Supplementary Fig. 1. Original data points in graphs are shown in the source data files. Statistical analyses of this study are provided in Supplementary Table 4. The sequences of *Bisp* have been deposited and made publicly available in GenBank with accession number MH885414. Source data are provided with this paper.

## Source data

Source Data Fig. 1
Source Data Fig. 2
Source Data Fig. 3
Source Data Fig. 4
Source Data Fig. 5
Source Data Extended Data Fig. 1
Source Data Extended Data Fig. 2
Source Data Extended Data Fig. 3
Source Data Extended Data Fig. 4
Source Data Extended Data Fig. 5
Source Data Extended Data Fig. 6
Source Data Extended Data Fig. 7
Source Data Extended Data Fig. 8
Source Data Extended Data Fig. 10
