# Supplementary material for: A tripartite rheostat controls self-regulated host plant resistance to insects
Source: Nature. 2023 Jun 14;618(7966):799–807. doi: 10.1038/s41586-023-06197-z (PMC10284691; doi:10.1038/s41586-023-06197-z)

nature portfolio

https://doi.org/10.1038/s41586-023-06197-z

Supplementary information

A tripartite rheostat controls self-regulated host plant resistance to insects

In the format provided by the authors and unedited

Figure 1d

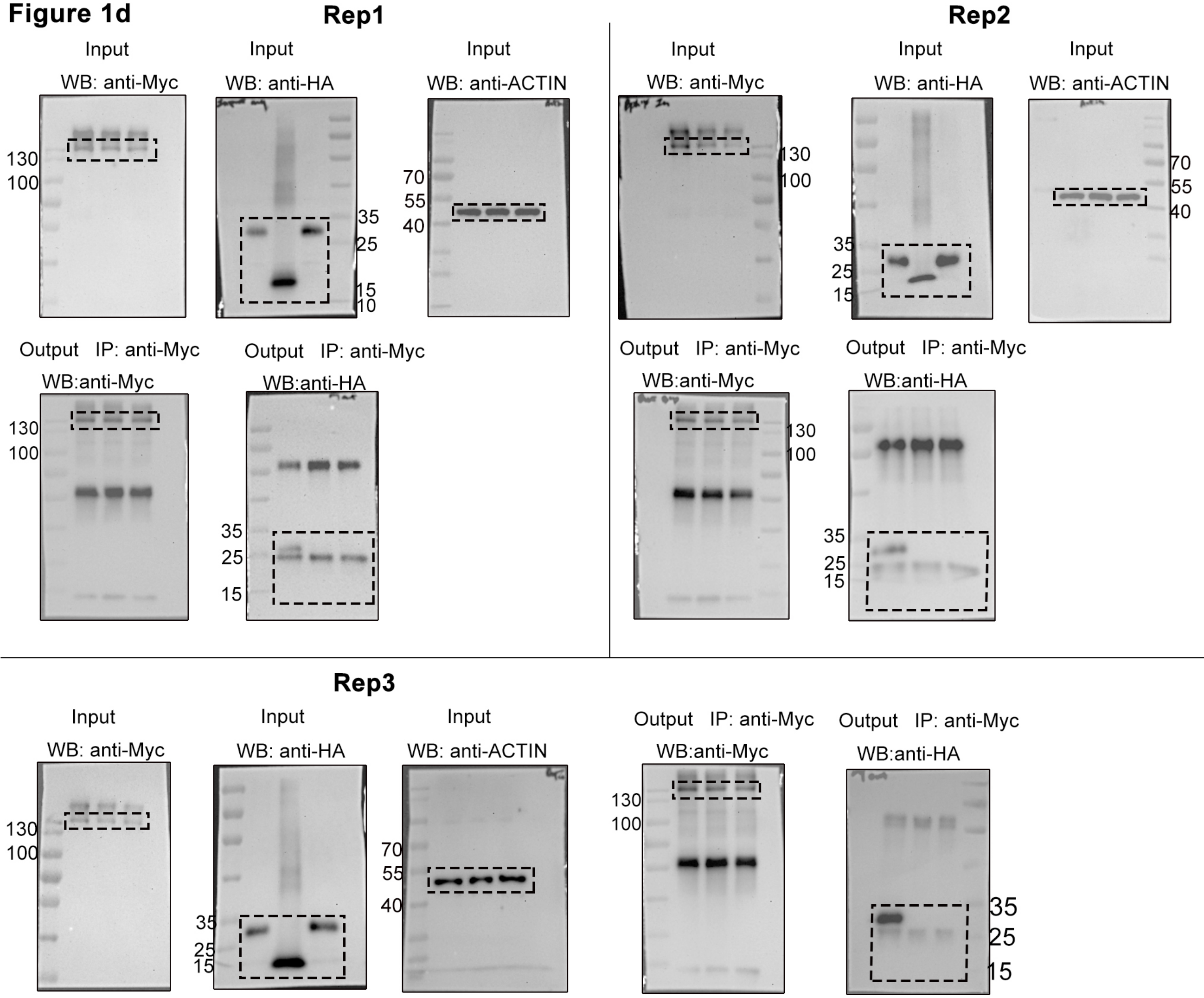

Figure 1i

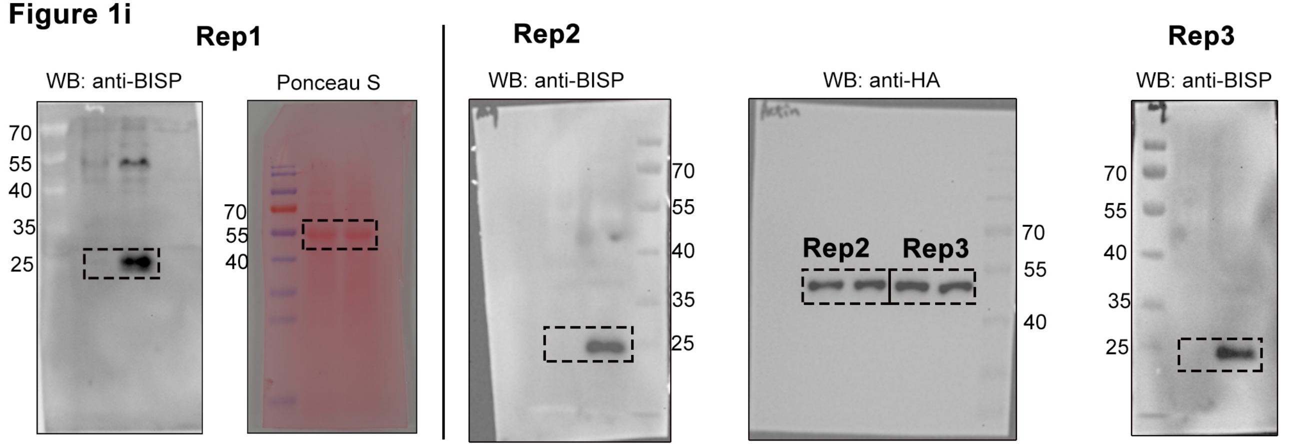

Figure 2a

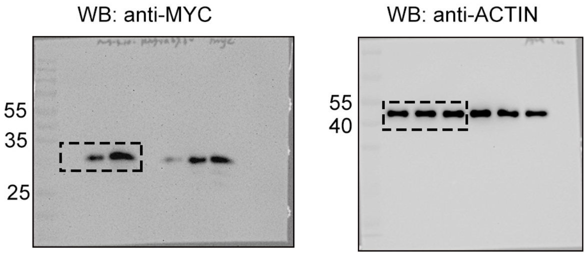

Figure 2f

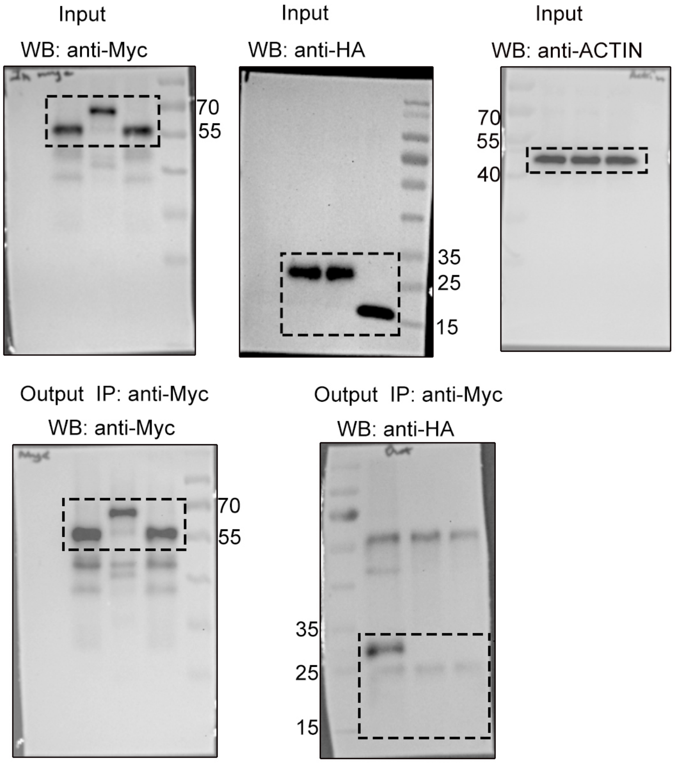

Figure 2g

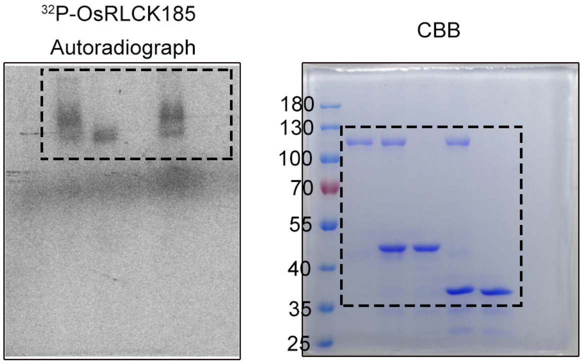

Figure 3b

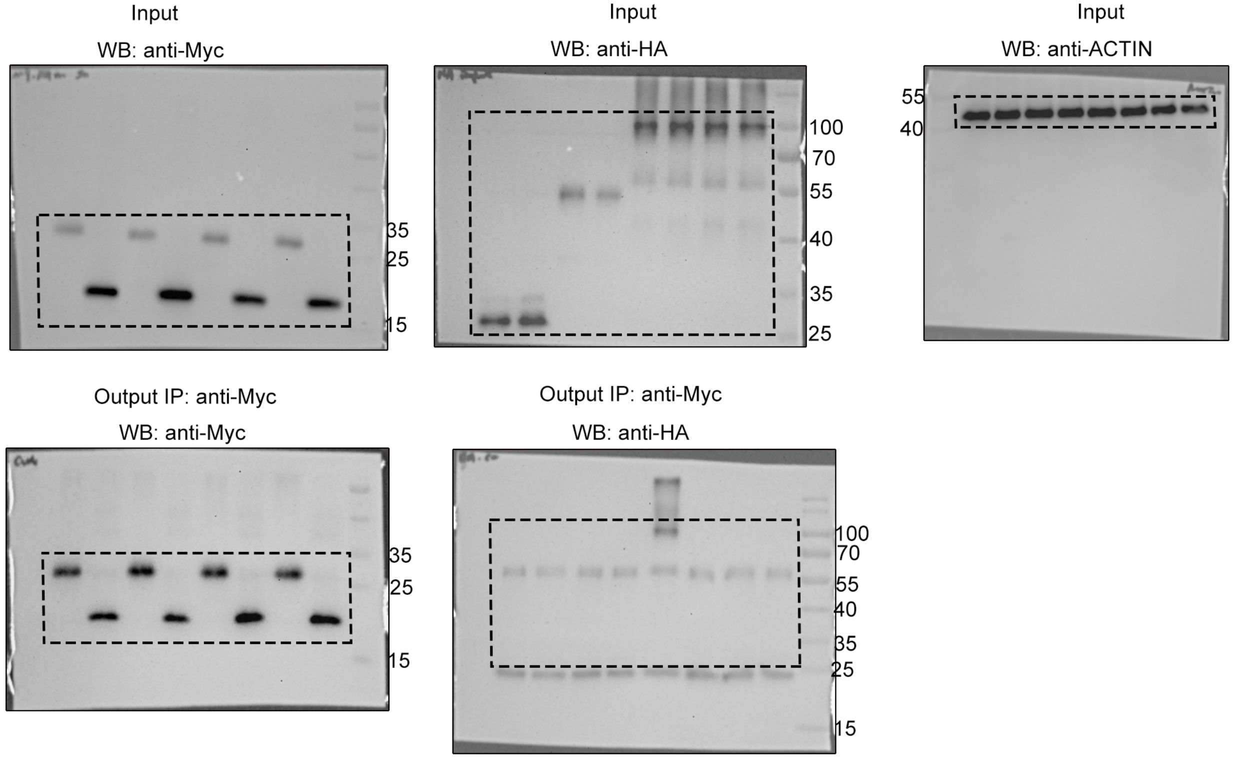

Figure 3c

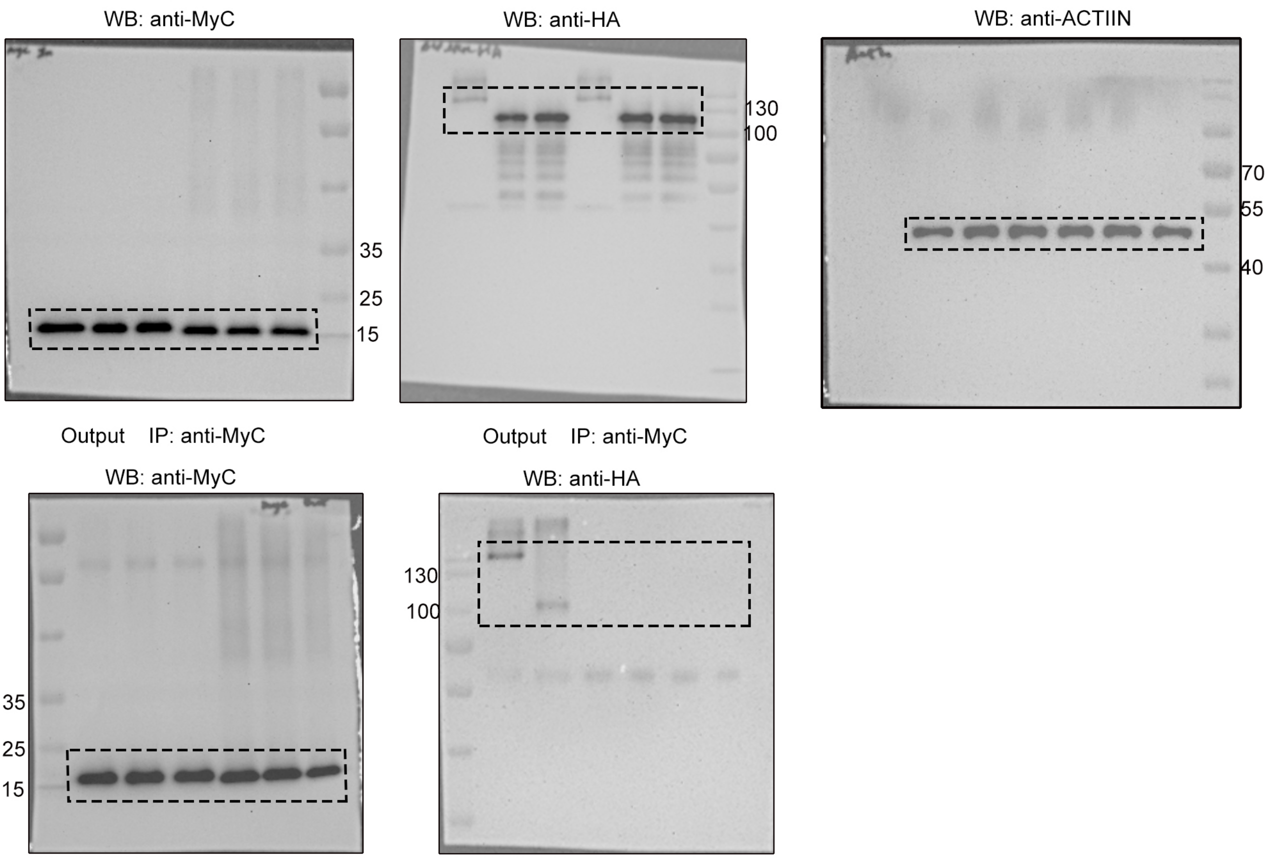

Figure 3f

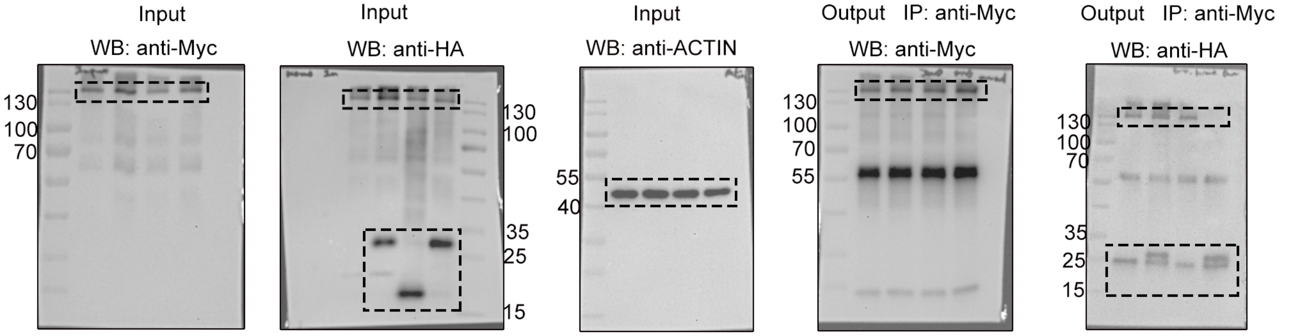

Figure 3g

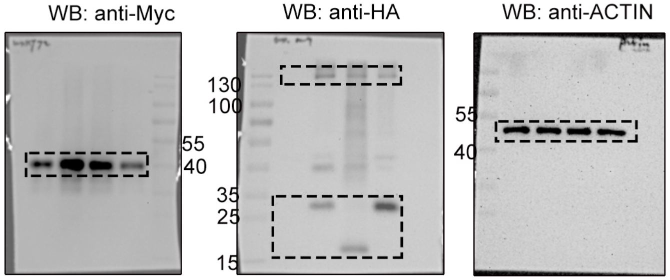

Figure 3h

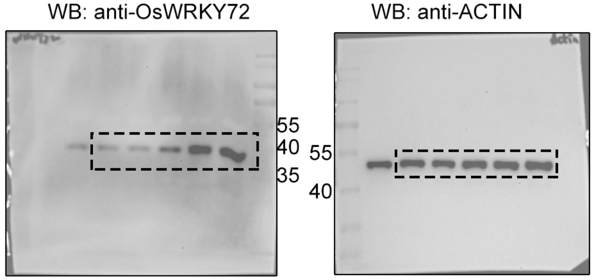

Figure 4a

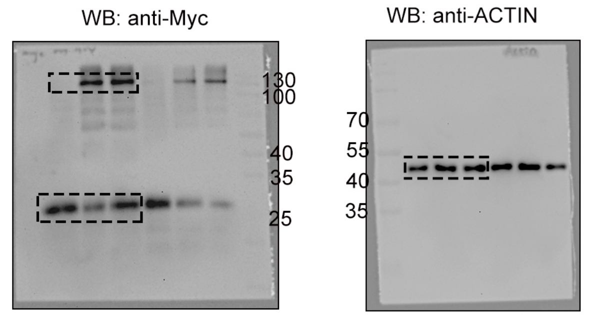

Figure 4b

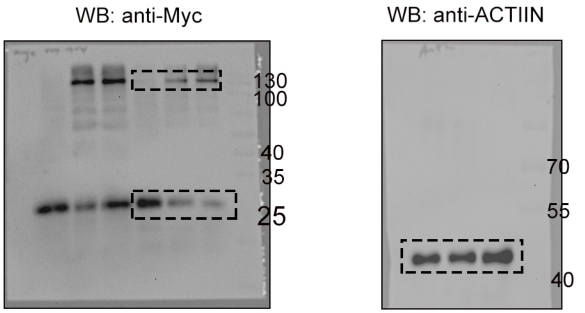

Figure 4c

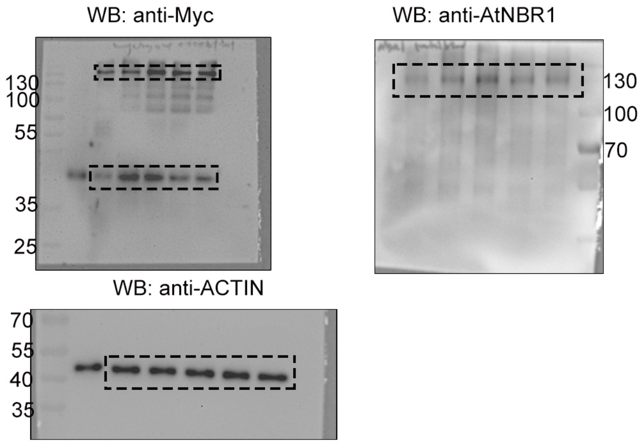

Figure 4f

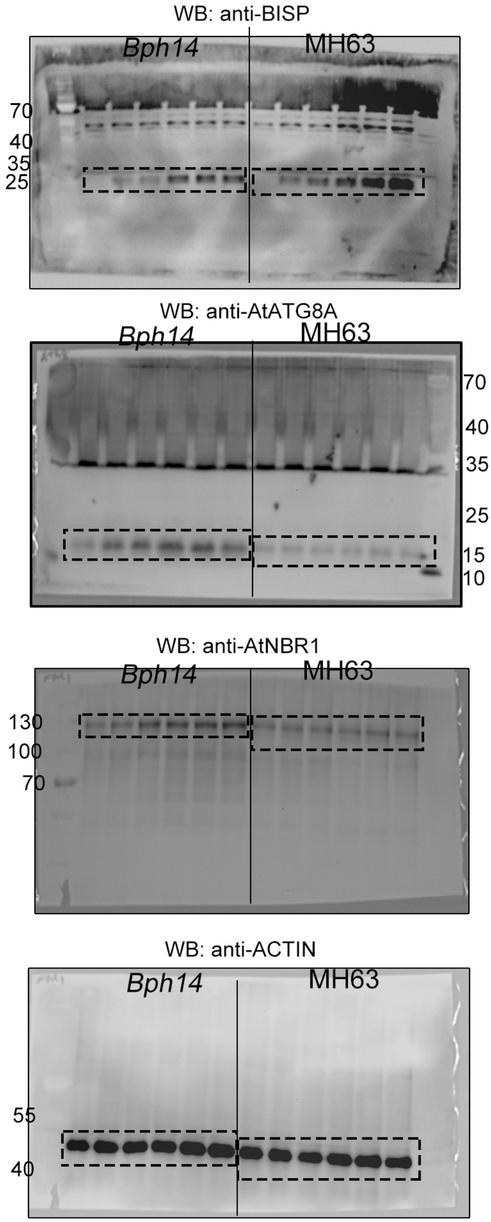

Figure 4h

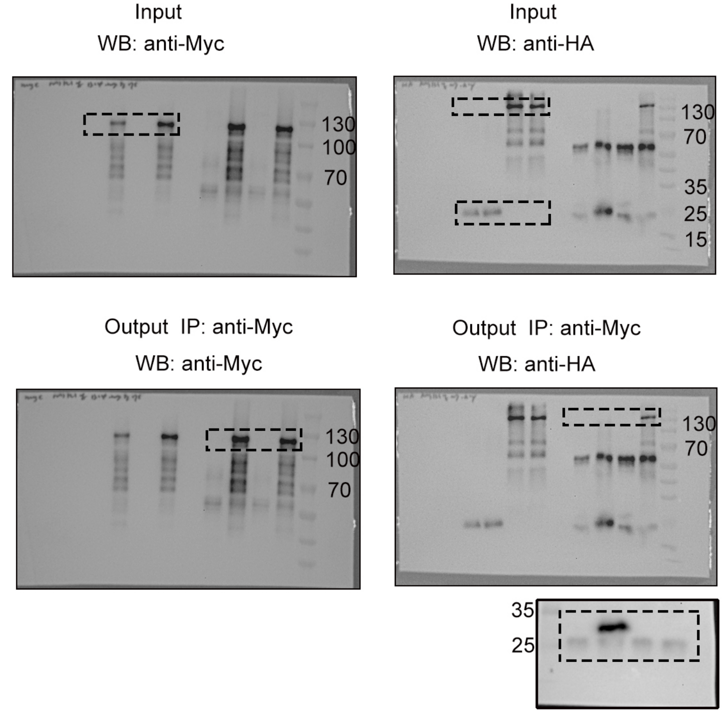

Figure 4i

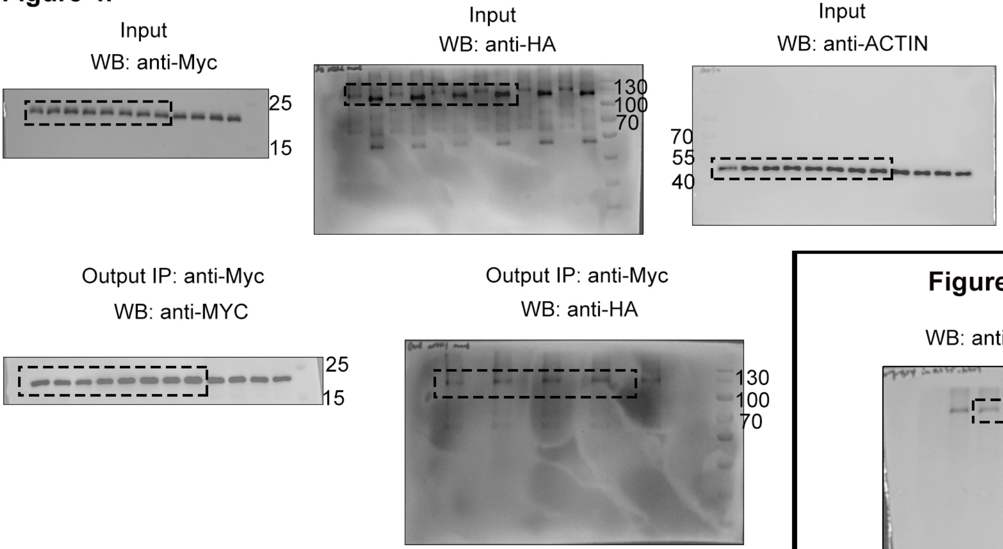

Figure 4j

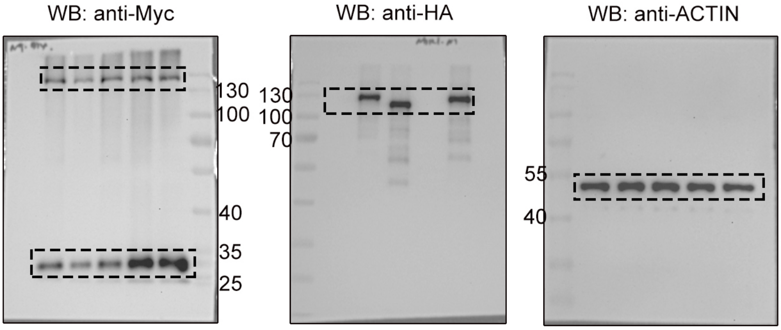

Figure 4k

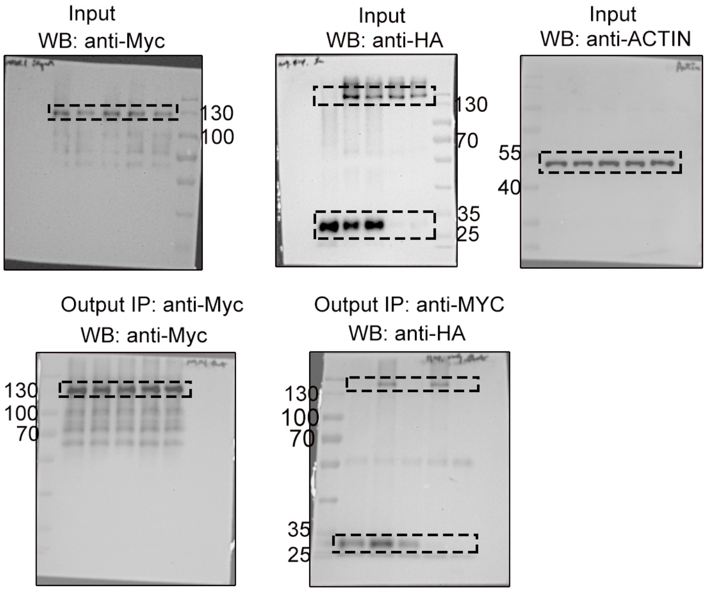

Figure 4l

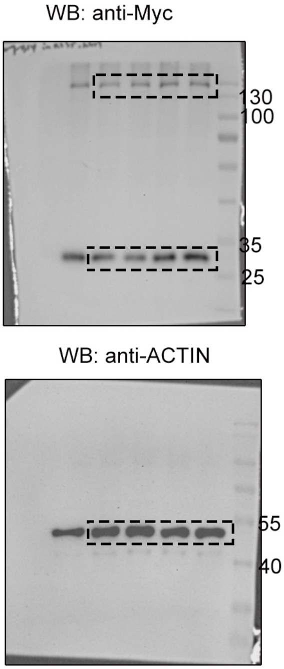

Figure 4m

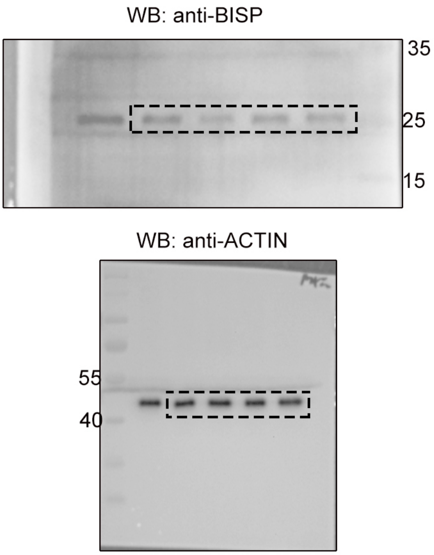

Figure 5a

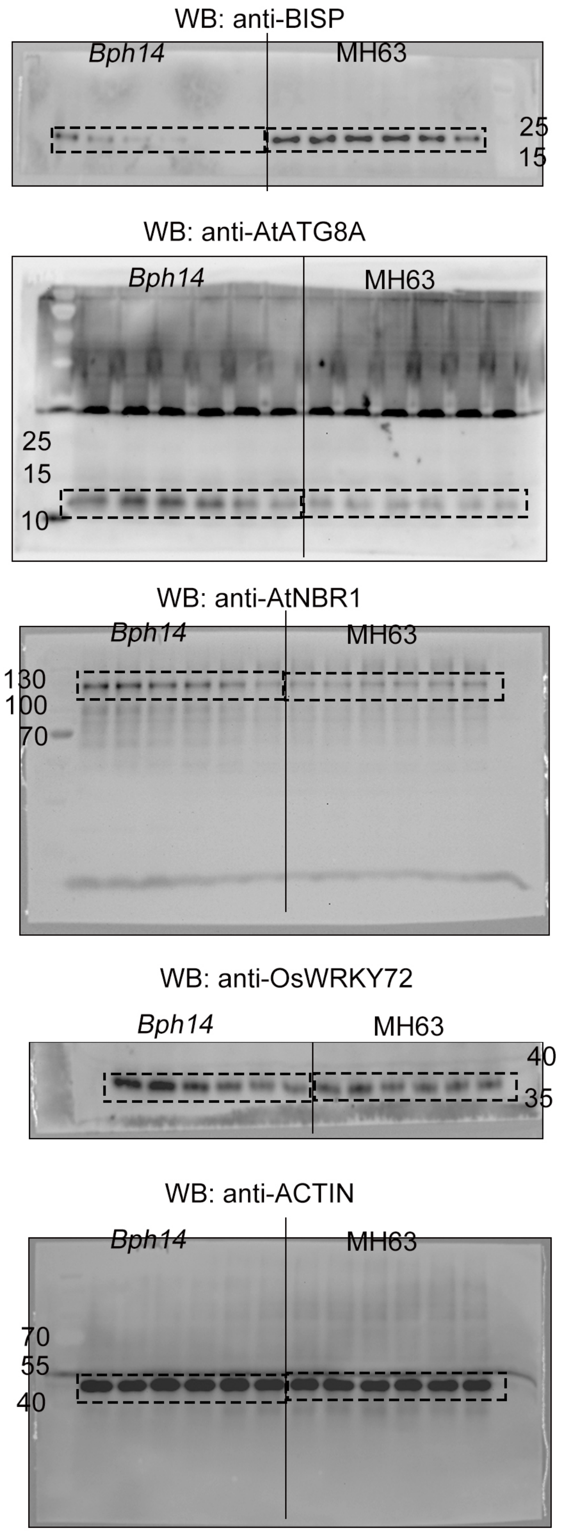

Figure 5c

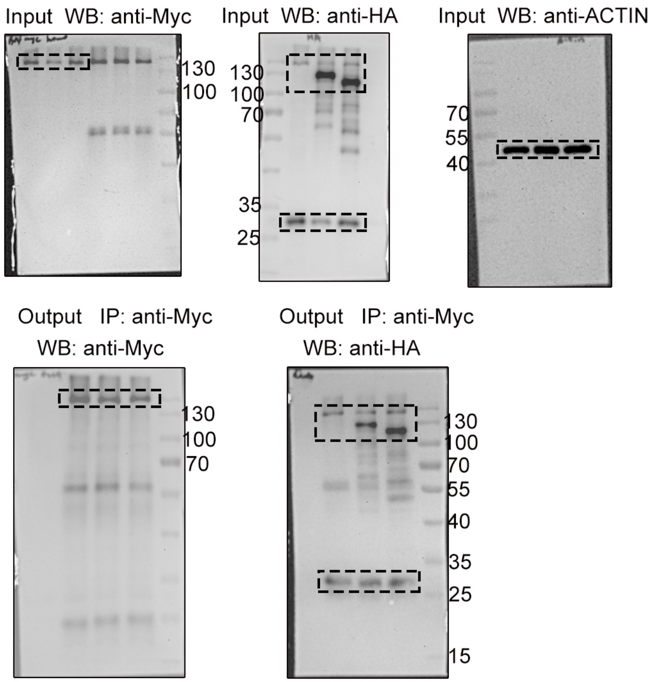

Figure 5d

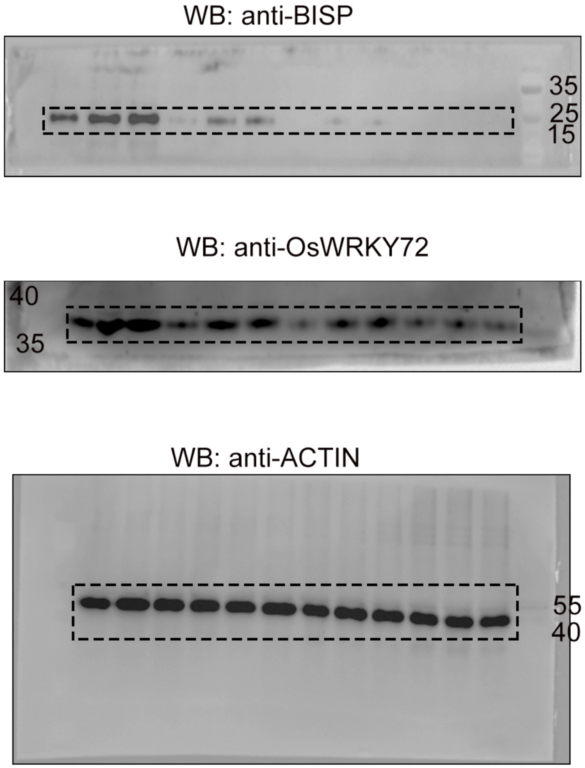

Extended Data Figure 1d

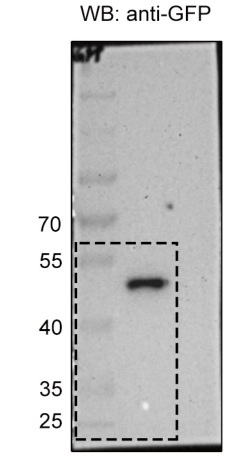

Extended Data Figure 1e

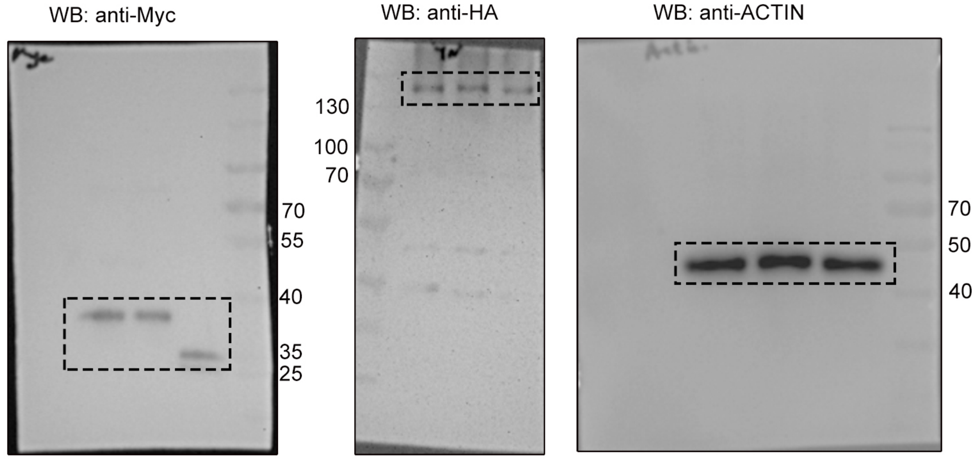

Extended Data Figure 1h

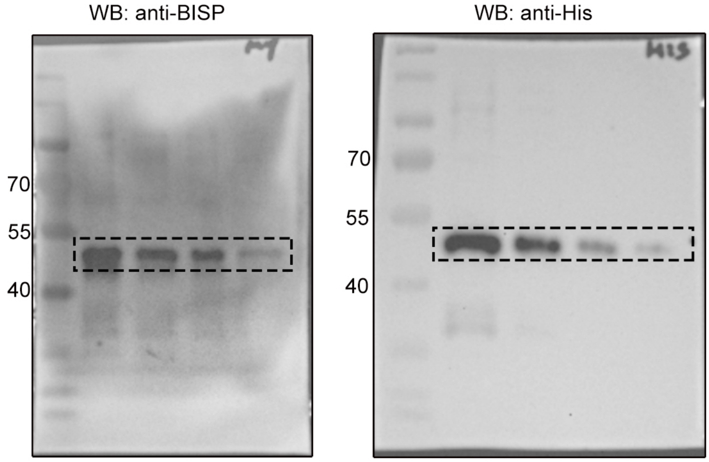

Extended Data Fig.ure 1i

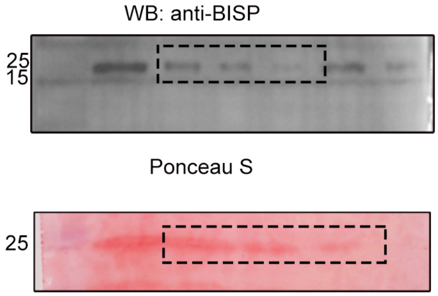

Extended Data Figure 2a

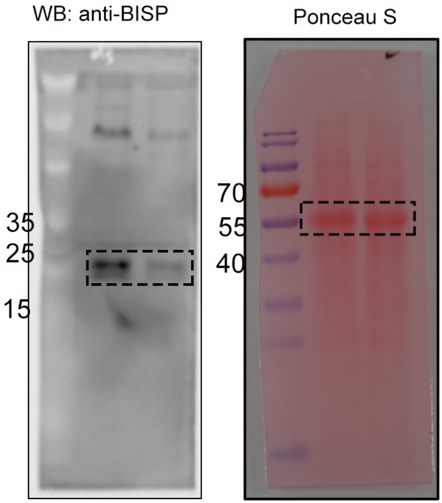

Extended Data Figure 4b

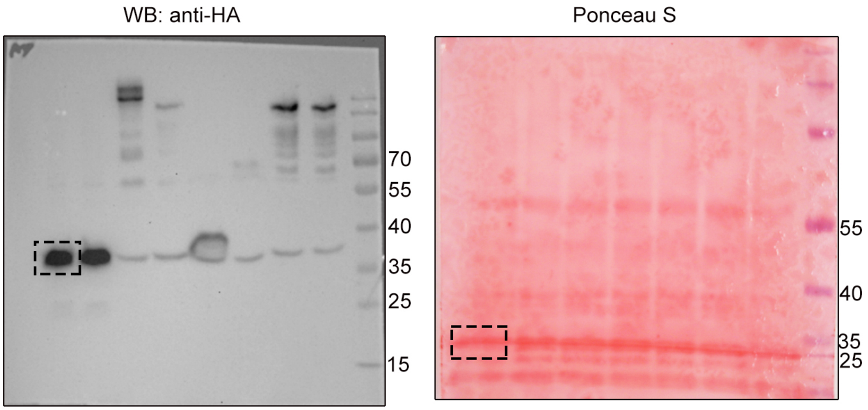

Extended Data Figure 4b

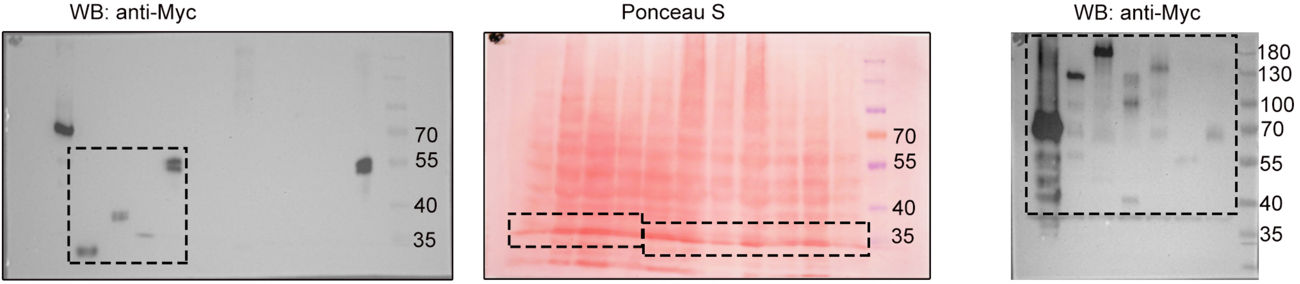

Extended Data Figure 4d

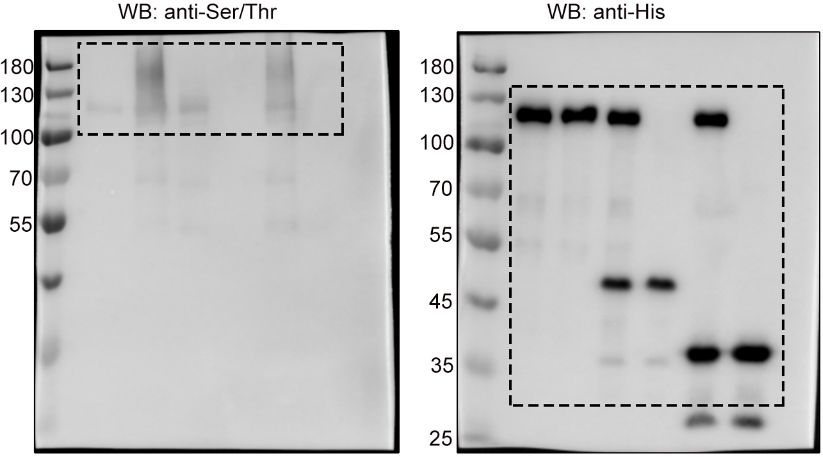

Extended Data Figure 7a

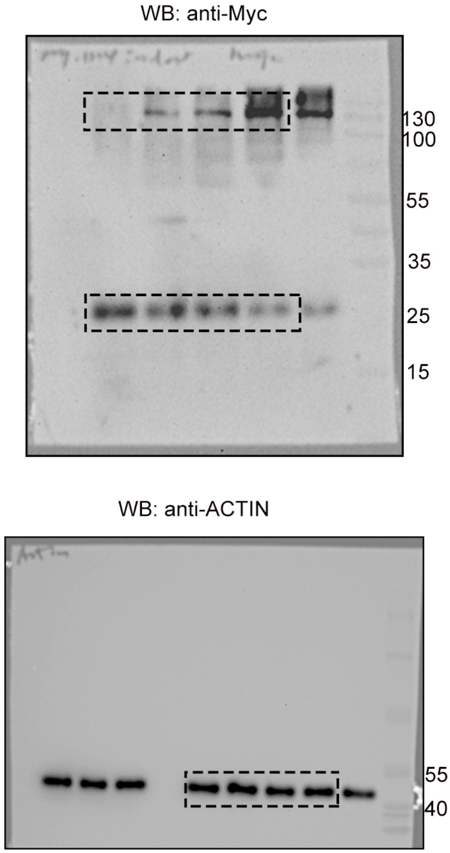

Extended Data Figure 5g

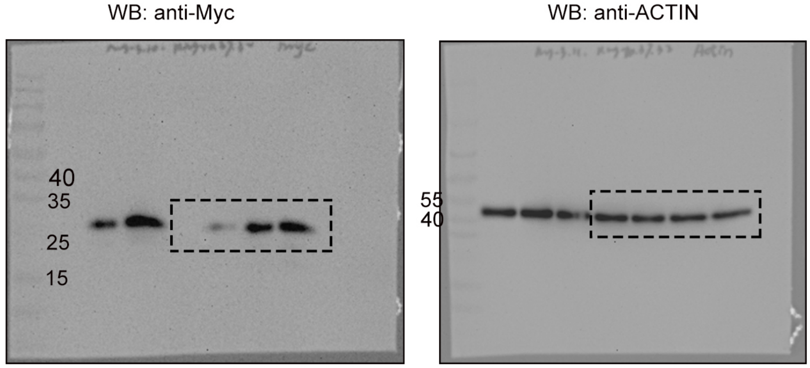

Extended Data Figure 7b

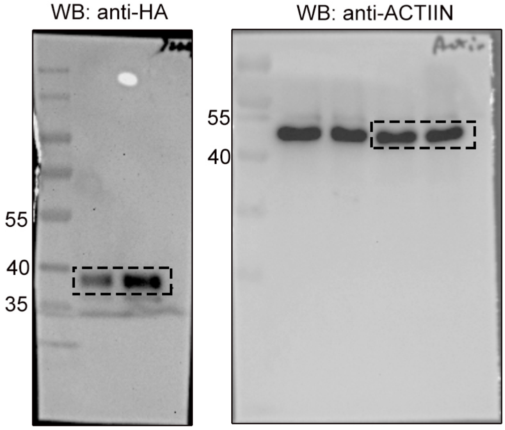

Extended Data Figure 7c

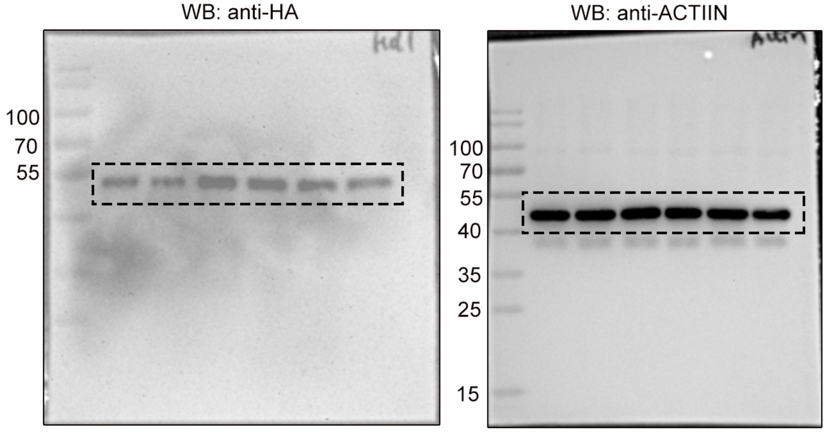

Extended Data Figure 7g

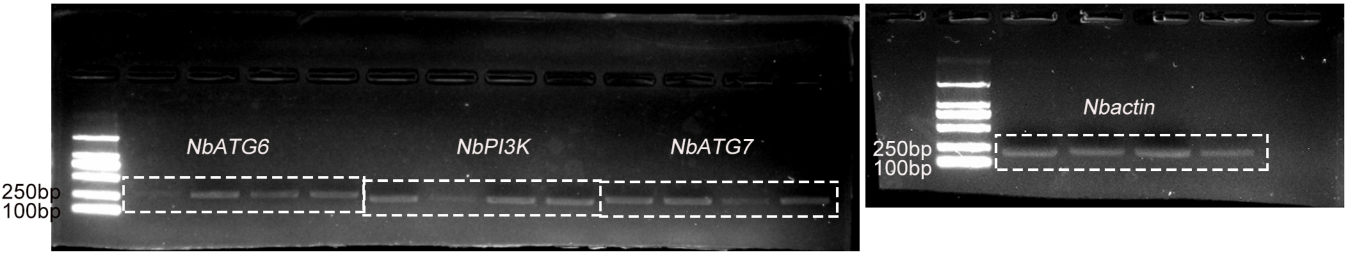

Extended Data Figure 7i

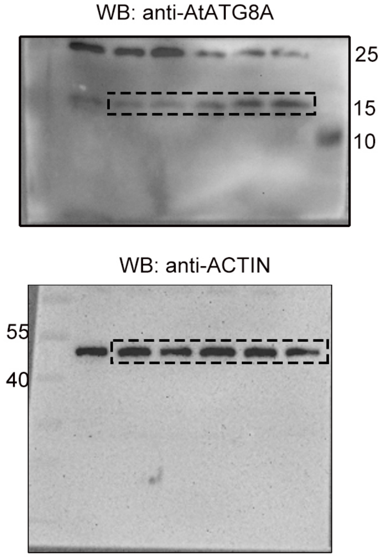

Extended Data Figure 7l

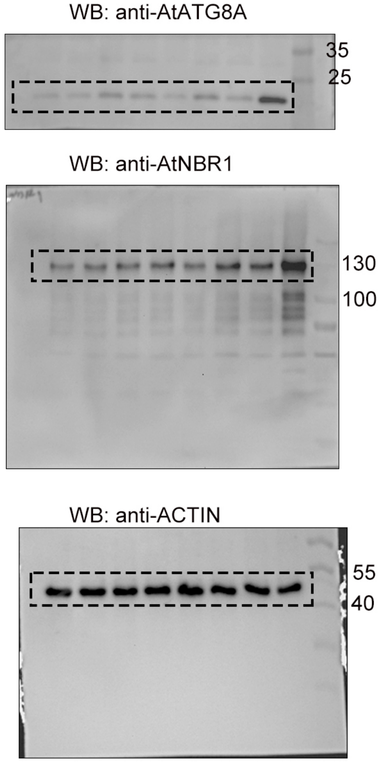

Extended Data Figure 8a

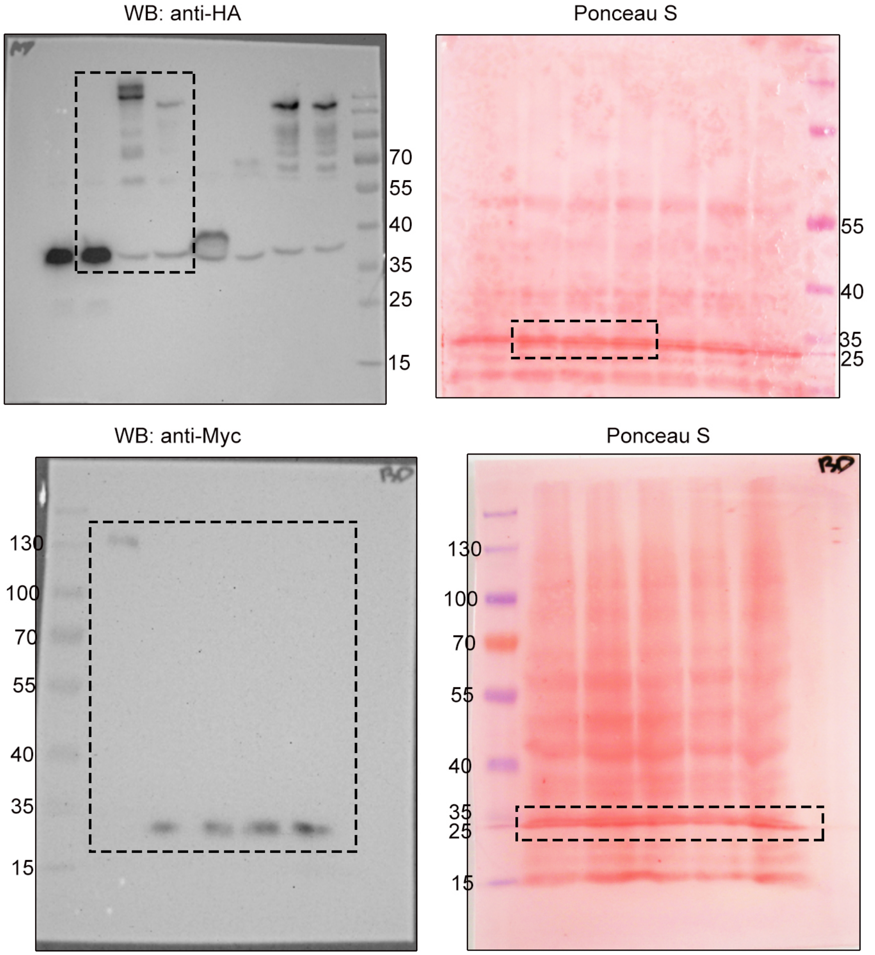

Extended Data Figure 8d

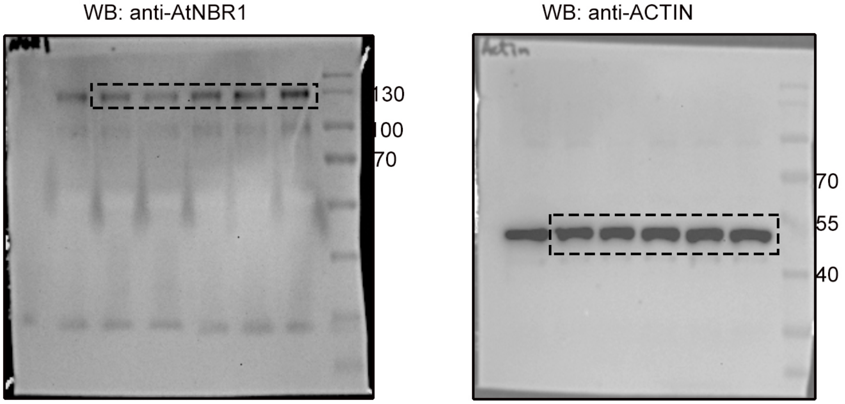

Extended Data Figure 9b

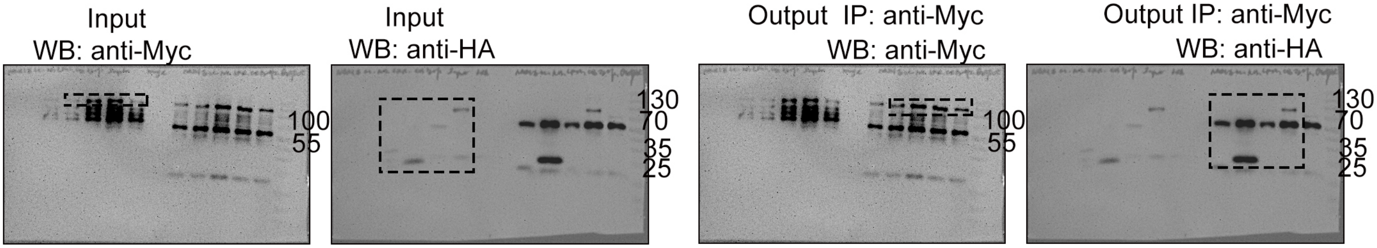

Extended Data Figure 9d

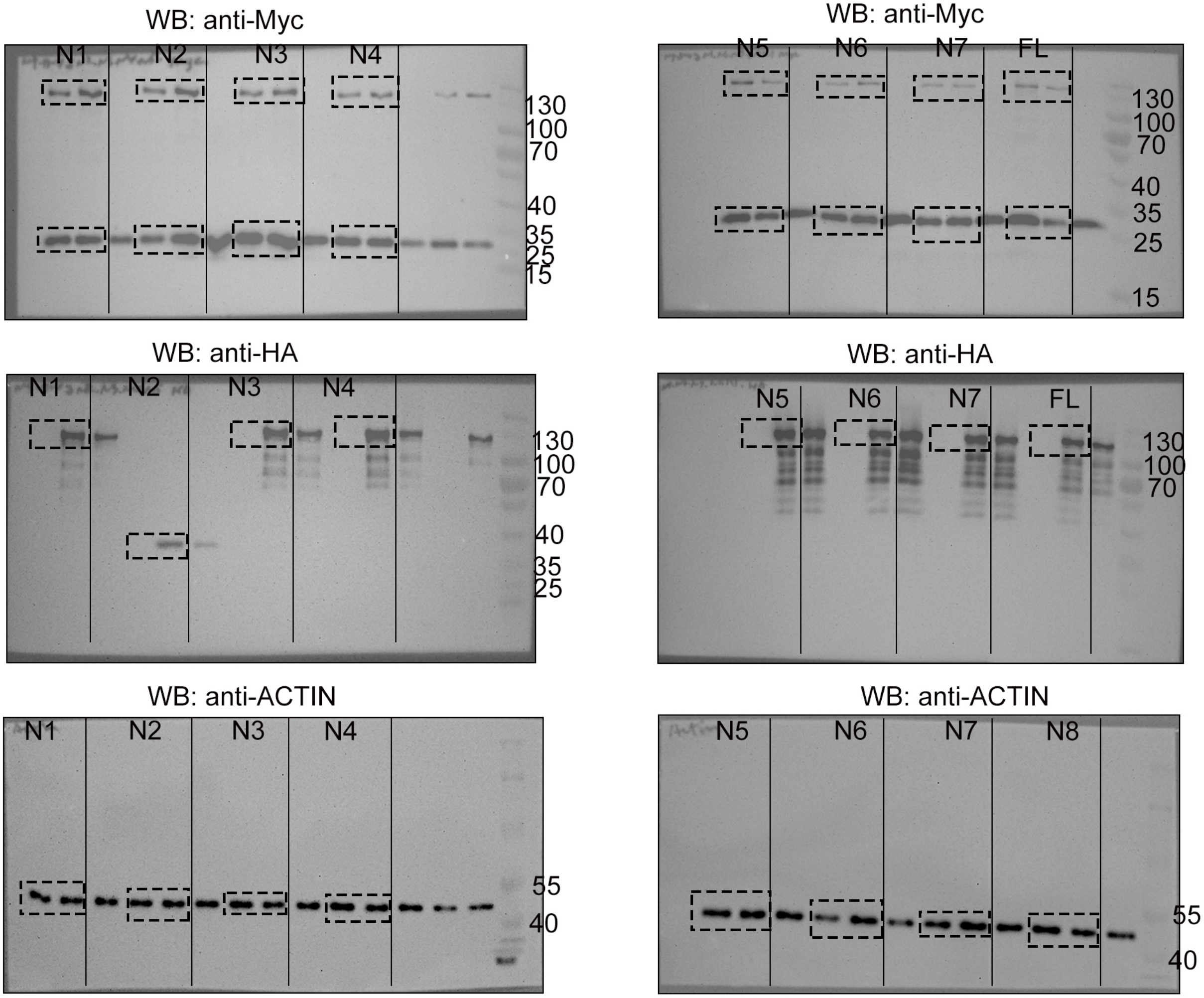

Extended Data Figure 9e

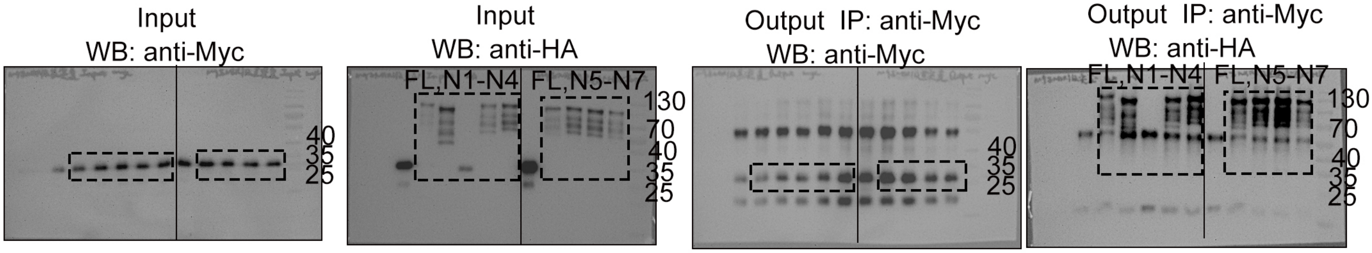

Extended Data Figure 9f

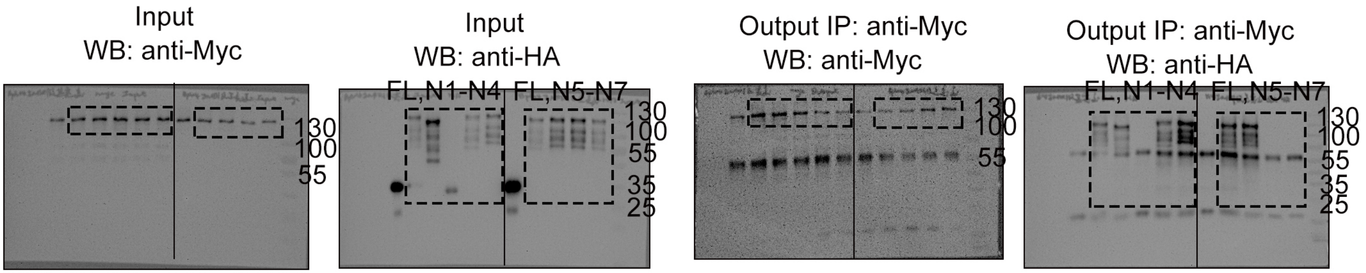

Extended Data Figure 9g

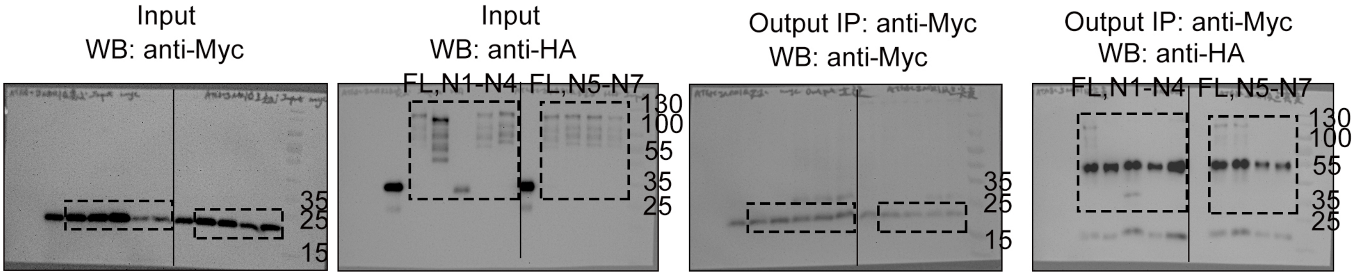

Extended Data Figure 9h

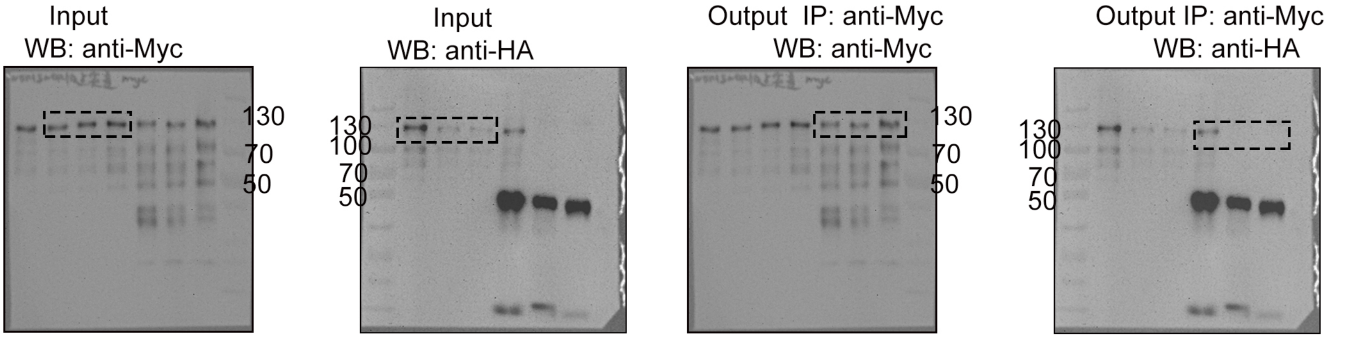

Extended Data Figure 9k

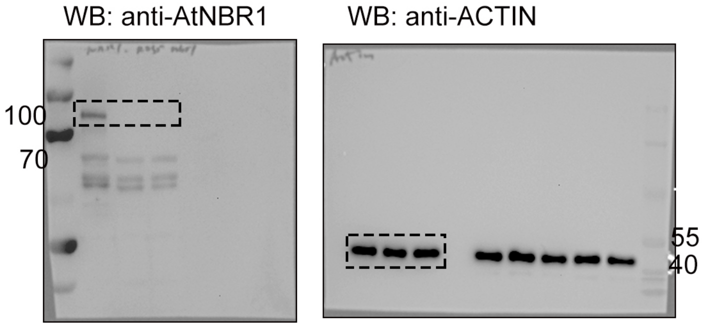

Supplement: Supplementary file 1 — Uncropped blots and gel images. [file 41586_2023_6197_MOESM1_ESM.pdf]
