# Supplementary material for: A tripartite rheostat controls self-regulated host plant resistance to insects
Source: Nature. 2023 Jun 14;618(7966):799–807. doi: 10.1038/s41586-023-06197-z (PMC10284691; doi:10.1038/s41586-023-06197-z)
Supplement: Supplementary file 2 — Reporting Summary [file 41586_2023_6197_MOESM2_ESM.pdf]

nature portfolio

Corresponding author(s): Guangcun He

Last updated by author(s): Apr 6, 2023

Reporting Summary

Nature Portfolio wishes to improve the reproducibility of the work that we publish. This form provides structure for consistency and transparency in reporting. For further information on Nature Portfolio policies, see our Editorial Policies and the Editorial Policy Checklist.

Statistics

For all statistical analyses, confirm that the following items are present in the figure legend, table legend, main text, or Methods section.

n/a	Confirmed	
☐	☒	The exact sample size (*n*) for each experimental group/condition, given as a discrete number and unit of measurement
☐	☒	A statement on whether measurements were taken from distinct samples or whether the same sample was measured repeatedly
☐	☒	The statistical test(s) used AND whether they are one- or two-sided<br>*Only common tests should be described solely by name; describe more complex techniques in the Methods section.*
☒	☐	A description of all covariates tested
☐	☒	A description of any assumptions or corrections, such as tests of normality and adjustment for multiple comparisons
☐	☒	A full description of the statistical parameters including central tendency (e.g. means) or other basic estimates (e.g. regression coefficient) AND variation (e.g. standard deviation) or associated estimates of uncertainty (e.g. confidence intervals)
☐	☒	For null hypothesis testing, the test statistic (e.g. *F*, *t*, *r*) with confidence intervals, effect sizes, degrees of freedom and *P* value noted<br>*Give P values as exact values whenever suitable.*
☒	☐	For Bayesian analysis, information on the choice of priors and Markov chain Monte Carlo settings
☒	☐	For hierarchical and complex designs, identification of the appropriate level for tests and full reporting of outcomes
☒	☐	Estimates of effect sizes (e.g. Cohen's *d*, Pearson's *r*), indicating how they were calculated

Our web collection on statistics for biologists contains articles on many of the points above.

Software and code

Policy information about availability of computer code

Data collection	The fluorescence signal was detected using a confocal microscope (Leica, DMi8).<br>Images from immuno blotting were collected with Tanon MP (5500) or Bio-Rad ChemiDoc Imaging System.<br>BLI experiments were performed using the Octet RED96 system (FortéBio).<br>MST experiments were performed using Monolith NT.115 (Nanotemper Technologies).<br>The expression levels of genes were analyzed by a CFX96 Real-Time System (Bio-Rad) .<br>The BPHs dsRNA injection was used using a Nanoliter 2010 injector (World Precision Instruments).
Data analysis	RNA analysis: CFX Manager Software (version 2.1)<br>Image analysis: ImageJ (version 1.45)<br>Confocal microscope: LAS AF Software (2.6.0 build 7266)<br>Transmission electron microscopy: JEM-1230 electron microscope<br>BLI analysis: FortéBio data analysis software (v.1.1.0.16, FortéBio)<br>MST analysis:MO. Affinity Analysis software (v2.3)<br>Ki analysis: Ki Finder software (http://www.nanotemper technologies.com/get-it-all/tools/ki-finder/)<br>Statistical analysis: GraphPad Prism (version 8.01), Excel 2016 (Microsoft)

For manuscripts utilizing custom algorithms or software that are central to the research but not yet described in published literature, software must be made available to editors and reviewers. We strongly encourage code deposition in a community repository (e.g. GitHub). See the Nature Portfolio guidelines for submitting code & software for further information.

Data

Policy information about availability of data

All manuscripts must include a data availability statement. This statement should provide the following information, where applicable:

- Accession codes, unique identifiers, or web links for publicly available datasets
- A description of any restrictions on data availability
- For clinical datasets or third party data, please ensure that the statement adheres to our policy

All data are available within this Article and its Supplementary Information. Original gel blots are shown in Supplementary Figure 1. Original data points in graphs are shown in the Source Data files. Statistical analyses of this study are provided in Supplementary Table 4. The sequence of Bisp has been deposited and made publicly available in GenBank with accession number MH885414.

Human research participants

Policy information about studies involving human research participants and Sex and Gender in Research.

Reporting on sex and gender	*Use the terms sex (biological attribute) and gender (shaped by social and cultural circumstances) carefully in order to avoid confusing both terms. Indicate if findings apply to only one sex or gender; describe whether sex and gender were considered in study design whether sex and/or gender was determined based on self-reporting or assigned and methods used. Provide in the source data disaggregated sex and gender data where this information has been collected, and consent has been obtained for sharing of individual-level data; provide overall numbers in this Reporting Summary. Please state if this information has not been collected. Report sex- and gender-based analyses where performed, justify reasons for lack of sex- and gender-based analysis.*
Population characteristics	*Describe the covariate-relevant population characteristics of the human research participants (e.g. age, genotypic information, past and current diagnosis and treatment categories). If you filled out the behavioural & social sciences study design questions and have nothing to add here, write "See above."*
Recruitment	*Describe how participants were recruited. Outline any potential self-selection bias or other biases that may be present and how these are likely to impact results.*
Ethics oversight	*Identify the organization(s) that approved the study protocol.*

Note that full information on the approval of the study protocol must also be provided in the manuscript.

Field-specific reporting

Please select the one below that is the best fit for your research. If you are not sure, read the appropriate sections before making your selection.

☒ Life sciences ☐ Behavioural & social sciences ☐ Ecological, evolutionary & environmental sciences

For a reference copy of the document with all sections, see nature.com/documents/nr-reporting-summary-flat.pdf

Life sciences study design

All studies must disclose on these points even when the disclosure is negative.

Sample size	The sample size and the results of statistical analyses are described in the relevant figures or method section. Sample size was determined based on experimental trials and previous publications on similar experiments (Du et al., 2009,PNAS, 106, 22163–22168; Guo et al., 2018, Nature genetics, 50, 297–306; Han et al., 2015, Plant Cell 27, 1316–1331; Shangguan et al., 2018, Plant Physiol. 176, 552–65; Yuan et al., 2021, Nature, 592, 105-109). No statistical methods were used to predetermine sample size.
Data exclusions	No data were excluded.
Replication	All experiments were successfully repeated at least two or three times. Results were reproducible in all repeats with the same trend.
Randomization	Plants were allocated with different genotypes and were grown side by side to minimize unexpected environmental variations during growth and experimantation. Plants samples were collected randomly for all expreiments with no formal randomization techniques..
Blinding	Investigators were not blinded to the allocation during experiments as it does not include clinical trials. The research materials are plants so the blind design is not applicable in the field (partially because different plant genotypes may grow differently and show different morphology[illegible]making blinding impossible). Researchers were not blinded to plant genotypes during experiments. Experiments were conducted by different authors, whenever possible.

Reporting for specific materials, systems and methods

We require information from authors about some types of materials, experimental systems and methods used in many studies. Here, indicate whether each material, system or method listed is relevant to your study. If you are not sure if a list item applies to your research, read the appropriate section before selecting a response.

Materials & experimental systems

n/a	Involved in the study
☐	☒ Antibodies
☐	☒ Eukaryotic cell lines
☒	☐ Palaeontology and archaeology
☐	☒ Animals and other organisms
☒	☐ Clinical data
☒	☐ Dual use research of concern

Methods

n/a	Involved in the study
☒	☐ ChIP-seq
☒	☐ Flow cytometry
☒	☐ MRI-based neuroimaging

Antibodies

Antibodies used

anti-HA (MBL, catalog no. M180-3, 1:2000)
anti-HA mAb-HRP-DirecT (MBL, catalog no. M180-7, Clone: TANA2, 1:1000)
anti-Myc (MBL, Catalo no.: M192-3, 1:2000)
anti-Myc mAb-HRP-DirecT (MBL, catalog no. M192-7, 1:1000, Clone: My3, 1:1000)
anti-GFP (Roche, catalog no. 11814460001, 1:1000)
anti-ACTIN (Abbkine, catalog no. ABL1050, 1:3000)
anti-OsWRKY72 (Beijing Protein Innovation, catalog no. AbP80456-A-SE, 1:500)
anti-AtATG8A (Abcam, catalog no. ab77003, 1:1000)
anti-AtNBR1 (Agrisera, catalog no. AS194281, 1:1000)
anti-BISP (custom-developed by DIA·AA Biotech Corp, China, 1:500)
anti-NlSP1 (custom-developed by DIA·AA Biotech Corp, China, 1:500)
anti-pSer/Thr (Millipore, catalog no. 05-368, 1:1000)
anti-His (GenScript, catalog no. A00186, 1:2000)
Goat anti-Rabbit IgG (H+L) Secondary Antibody, HRP conjugate (GenScript, catalog no. A00098, 1:10000)
Goat anti-mouse IgG (H+L) Secondary Antibody, HRP conjugate (GenScript, catalog no. A00160, 1:10000)
Goat anti-rabbit IgG (H+L) Secondary Antibody, Cy3 conjugate (Jackson ImmunoResearch, catalog no. 111-165-003, 1:500)
anti-rabbit IgG gold-coupled secondary antibodies (Sigma-Aldrich, catalog no. G7277- .4ML, 1: 50)

Validation

Most of the antibodies used are commercially and can be obtained from the following websites and publications:
anti-HA (https://www.mblbio.com/bio/g/dtl/A/?pcd=M180-3)
anti-HA mAb-HRP-DirecT (https://www.mblbio.com/bio/g/dtl/A/index.html?pcd=M180-7)
anti-Myc ((https://www.mblbio.com/bio/g/dtl/A/?pcd=M192-3)
anti-Myc mAb-HRP-DirecT (https://www.mblbio.com/bio/g/dtl/A/index.html?pcd=M192-7)
anti-ACTIN (https://www.abbkine.com/product/anti-plant-actin-mouse-monoclonal-antibody-3t3-abl1050/)
anti-GFP (https://www.sigmaaldrich.cn/CN/zh/product/roche/11814460001)
anti-OsWRKY72 (http://www.proteomics.org.cn/product/1022.html)
anti-AtATG8A (https://www.abcam.cn/apg8aatg8a-antibody-ab77003.html)
anti-AtNBR1 (https://www.agrisera.com/en/artiklar/nbr1-2.html)
anti-BISP (custom-developed by DIA·AA Biotech Corp, China, Extended data Fig. 1h,i &2a in this study)
anti-NlSP1 (ustom-developed by DIA·AA Biotech Corp, China, Huang et al. 2020. Front Plant Sci. 11:571280)
anti-pSer/Thr (https://www.merckmillipore.com/CN/en/product/Anti-phospho-Ser-Thr-Pro-MPM-2-Antibody,MM_NF-05-368)
anti-His (https://www.genscript.com/antibody/A00186-THE_His_Tag_Antibody_mAb_Mouse.html)
Goat anti-Rabbit IgG (H+L) Secondary Antibody, HRP conjugate (https://www.genscript.com/antibody/A00098-Goat_Anti_Rabbit_IgG_Antibody_H_L_HRP_pAb.html)
Goat anti-mouse IgG (H+L) Secondary Antibody, HRP conjugate (https://www.genscript.com/antibody/A00160-Goat_Anti_Mouse_IgG_Antibody_H_L_HRP_pAb_.html)
Goat anti-rabbit IgG (H+L) Secondary Antibody, Cy3 conjugate (https://www.jacksonimmuno.com/catalog/products/111-165-003)
anti-rabbit IgG gold-coupled secondary antibodies (https://www.sigmaaldrich.cn/CN/zh/product/sigma/g7277)

Eukaryotic cell lines

Policy information about cell lines and Sex and Gender in Research

Cell line source(s)	Standard SF9 baculovirus cell lines (Thermo Fisher Scientific, catalog no. 11496015) were purchased.
Authentication	SF9 cells were not authenticated further after purchase.
Mycoplasma contamination	SF9 cells have not been tested for Mycoplasma aontamination.

Commonly misidentified lines (See ICLAC register)	No commonly misidentified lines were used.

Animals and other research organisms

Policy information about studies involving animals; ARRIVE guidelines recommended for reporting animal research, and Sex and Gender in Research

Laboratory animals	The brown planthopper (BPH) used in the experiments were maintained in greenhouse at Wuhan University, Hubei, China.
Wild animals	No wild animals were used in this study.
Reporting on sex	For BPH weight and honeydew excretion, RNA analysis, we used female or male BPH in the experiments..
Field-collected samples	No field-collected samples were used in this study.
Ethics oversight	No ethics oversight was required for this study.

Note that full information on the approval of the study protocol must also be provided in the manuscript.
