# Supplementary figures and images for: A tripartite rheostat controls self-regulated host plant resistance to insects
Source: Nature. 2023 Jun 14;618(7966):799–807. doi: 10.1038/s41586-023-06197-z (PMC10284691; doi:10.1038/s41586-023-06197-z)

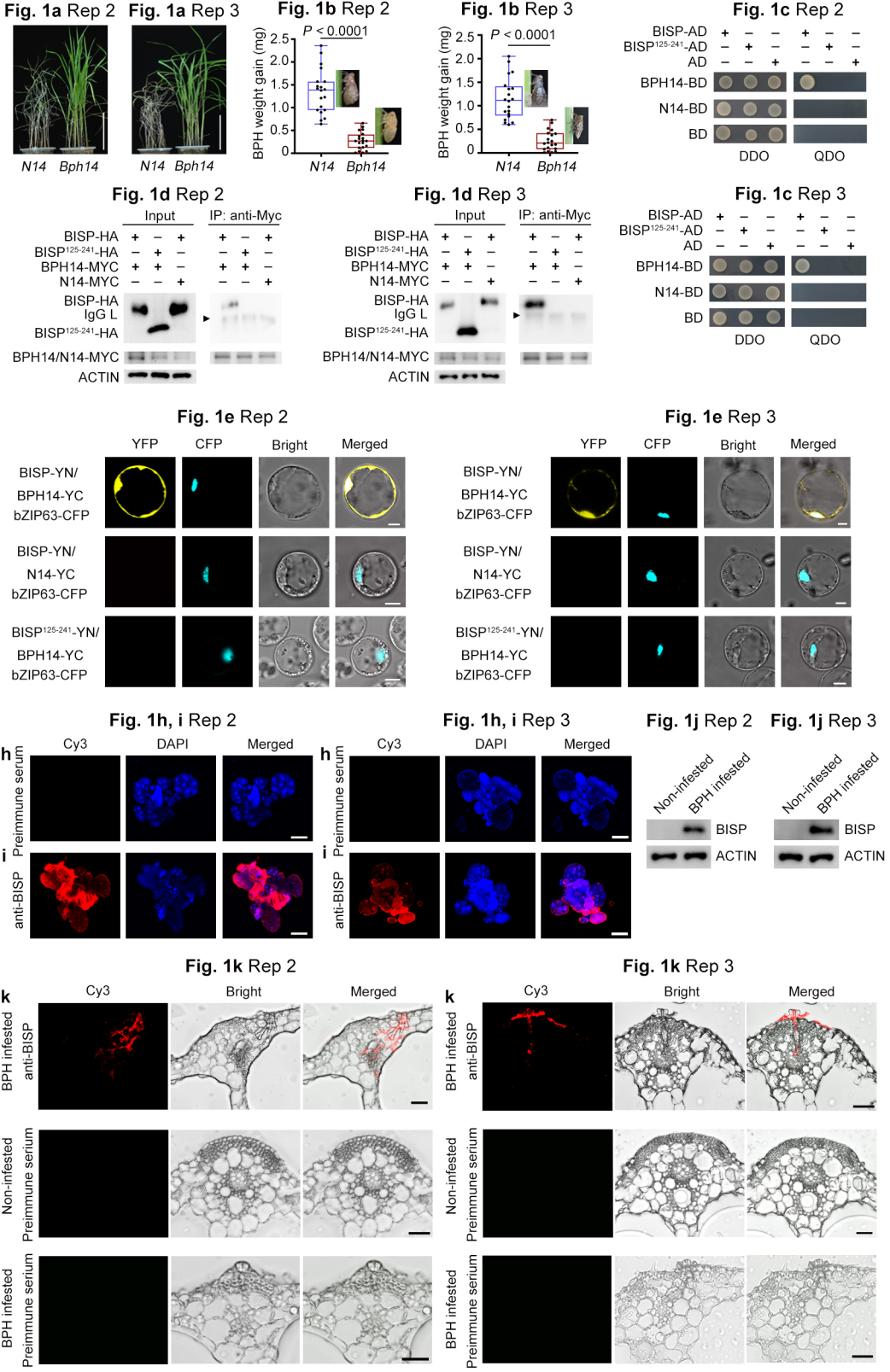

Supplement: Supplementary file 3 — The other two repeats of Fig. 1a–e,h–k. [file 41586_2023_6197_MOESM3_ESM.pdf]
